# Outcomes of an inpatient medical nutritional rehabilitation protocol in children and adolescents with eating disorders

**DOI:** 10.1186/s40337-017-0134-6

**Published:** 2017-03-01

**Authors:** Rebecka Peebles, Andrew Lesser, Courtney Cheek Park, Kerri Heckert, C. Alix Timko, Eleni Lantzouni, Ronald Liebman, Laurel Weaver

**Affiliations:** 1 The Children’s Hospital of Philadelphia, Department of Pediatrics, Division of Adolescent Medicine, Philadelphia, Pennsylvania USA; 2 The University of Pennsylvania, Perelman School of Medicine, Philadelphia, Pennsylvania USA; 3 The Children’s Hospital of Philadelphia, Department of Clinical Nutrition, Philadelphia, Pennsylvania USA; 4 The Children’s Hospital of Philadelphia, Department of Child and Adolescent Psychiatry and Behavioral Sciences, Philadelphia, Pennsylvania USA

**Keywords:** Anorexia nervosa, Bulimia nervosa, Eating disorders, Malnutrition, Refeeding syndrome, Adolescents, Nutritional rehabilitation

## Abstract

**Background:**

Medical stabilization through inpatient nutritional rehabilitation is often necessary for patients with eating disorders (EDs) but includes the inherent risk of refeeding syndrome. Here we describe our experience of implementing and sustaining an inpatient nutritional rehabilitation protocol designed to strategically prepare patients with EDs and their families for discharge to a home setting in an efficient and effective manner from a general adolescent medicine unit. We report outcomes at admission, discharge, and 4-weeks follow-up.

**Methods:**

Protocol development, implementation, and unique features of the protocol, are described. Data were collected retrospectively as part of a continuous quality improvement (QI) initiative. Safety outcomes were the clinical need for phosphorus, potassium, and magnesium supplementation, other evidence of refeeding syndrome, and unexpected readmissions within one month of discharge. The value outcome was length of stay (LOS). Treatment outcomes were the percentage median BMI (MBMI) change from admission to discharge, and from discharge to 4-weeks follow-up visit.

**Results:**

A total of 215 patients (88% F, 12% M) were included. Patients averaged 15.3 years old (5.8–23.2y); 64% had AN, 18% had atypical anorexia (AtAN), 6% bulimia nervosa (BN), 5% purging disorder (PD), 4% avoidant-restrictive food intake disorder (ARFID), and 3% had an unspecified food and eating disorder (UFED). Average LOS was 11 days. Initial mean calorie level for patients at admission was 1466 and at discharge 3800 kcals/day. Phosphorus supplementation for refeeding hypophosphatemia (RH) was needed in 14% of inpatients; full-threshold refeeding syndrome did not occur. Only 3.8% were rehospitalized in the thirty days after discharge. Patients averaged 86.1% of a median MBMI for age and gender, 91.4% MBMI at discharge, and 100.9% MBMI at 4-weeks follow-up. Mean percentage MBMI differences between time points were significantly different (admission-discharge: 5.3%, *p* <0.001; discharge-follow-up: 9.2%, *p* <0.001).

**Conclusions:**

Implementation of the CHOP inpatient nutritional rehabilitation protocol aimed at rapid, efficient, and safe weight gain and integration of caregivers in treatment of patients with diverse ED diagnoses led to excellent QI outcomes in percentage MBMI at discharge and 4-weeks follow-up, while maintaining a short LOS and low rates of RH phosphorus supplementation.

**Electronic supplementary material:**

The online version of this article (doi:10.1186/s40337-017-0134-6) contains supplementary material, which is available to authorized users.

## Plain English summary

Detailed reports on inpatient protocols for the management of adolescents with EDs are sparse; none involve patients with a variety of eating disorders undergoing refeeding in a hospital setting. Furthermore, no reports present workable ways to involve caregivers in care on an inpatient unit. Development, implementation, and short-term quality outcomes for an efficient and effective inpatient nutritional rehabilitation protocol for pediatric patients with eating disorders that strategically incorporates caregivers in treatment are reported. Patients were able to gain significant weight between admission, discharge, and follow-up, with low rates of phosphorus supplementation needed, and few patients requiring readmission. At our institution, a nutritional rehabilitation protocol achieved excellent short-term QI outcomes while involving families in direct care.

## Background

Medical stabilization through inpatient nutritional rehabilitation is often necessary for patients with eating disorders (EDs) but includes the inherent risk of refeeding syndrome [1–3]. Refeeding syndrome is a dangerous medical condition characterized by hypophosphatemia and other electrolyte abnormalities, which can lead to cardiac arrhythmia or even sudden death. Refeeding syndrome usually occurs within 72 – 84 h of initiating refeeding, and can develop when malnourished patients are rehabilitated too rapidly, so a “start low, advance slow” approach to calorie administration has been historically preferred for malnourished patients with anorexia nervosa (AN) and other EDs [4–10].

Recent studies have challenged this standard and have published analyses of their nutritional rehabilitation outcomes for adolescents with anorexia, comparing lower calorie levels at admission to higher ones [11–22]. These studies suggest that starting malnourished patients with AN on higher calorie diets can be carried out safely, without increased incidence of refeeding syndrome, and with shorter hospitalizations [1, 2]. Garber and colleagues (2013) demonstrated that it was important not only to start at a higher calorie amount, but also to advance calories more aggressively. Their study showed that slow caloric advancement of 200 calories every other day can delay nutritional repletion, increase lengths of hospitalization, and even lead to underfeeding in some cases [15]. Changing nutritional rehabilitation protocols to promote faster weight regain could particularly help malnourished patients with AN, as effective early weight gain is a positive predictor of future remission [23–26].

Family-based treatment (FBT) is an established, evidence-based standard for the treatment of EDs in pediatric patients, with improved long-term outcomes when compared with other treatments [27–29]. This treatment presents paradigm shifts from many other traditional ED treatments, empowering parents to be active members of the treatment team and re-nourish their children in ways that work in their family setting [30]. Weight gain in the first 4 weeks of FBT is predictive of future remission at 1-year post-treatment initiation [23, 24, 26]. However, FBT is an outpatient treatment and patients must be medically stable to receive care. Given that many patients are medically fragile when first diagnosed, there is a need to understand and develop inpatient protocols that can stabilize patients with EDs while also efficiently preparing them and their parents for outpatient care in FBT or other modalities.

Contributing to this lack of understanding is the dearth of detailed descriptions of nutritional rehabilitation protocols for pediatric patients in the literature. The absence of published descriptions is striking given that that standardized protocols and pathways often improve medical and psychiatric outcomes in other disease states [31]. Our protocol was designed to strategically prepare patients and families for discharge to a home setting in an efficient and effective manner and has led to successful outcomes for patients with malnutrition and EDs. Here we describe our experience of implementing and sustaining our inpatient nutritional rehabilitation protocol on a general adolescent medicine unit. We report outcomes at admission, discharge, and 4 week follow-up for pediatric patients with EDs, admitted to The Children’s Hospital of Philadelphia (CHOP) for the medical stabilization of complications of malnutrition.

## Methods

### Development and implementation

Before 2011, CHOP managed smaller volumes of patients with EDs in various pediatric treatment settings, but without optimal coordination between departments. Clinicians in the Department of Child and Adolescent Psychiatry and Behavioral Sciences, and its predecessor the Philadelphia Child Guidance Clinic, had a long history of treating patients with EDs by working with their families to refeed them [32, 33], but these techniques had not been well integrated in our hospital environment over the preceding decade. This often led to challenges when patients required inpatient medical stabilization. Prior inpatient protocols were safe but less compatible with an FBT focus in outpatient care. For example, inpatient protocols involved conservative calorie levels and slow weight gain, allowed for adolescents to self-select menus, and patients were often transferred directly to higher levels of psychiatric care, as defined by intensive outpatient, partial hospitalization, residential treatment, or inpatient psychiatric programs, rather than discharged to outpatient FBT.

In July 2011, The CHOP Eating Disorder Assessment and Treatment Program (EDATP) was initiated as a multi-department venture to incorporate evidence-based ED care and thereby improve inpatient and outpatient care for patients with EDs. In preparation for this launch, and with leadership support, a multidisciplinary taskforce of key stakeholders from the Departments of Pediatrics, Child Psychiatry and Behavioral Sciences, Nutrition, Social Work, and Nursing began to meet monthly in late 2010 to plan updates to our inpatient and outpatient protocols, incorporating and piloting the latest advances in evidence-based treatment. Subgroups from this taskforce met with the different Divisions and Departments involved to obtain feedback from physicians and staff to planned changes and input on areas still in need of revision.

Intensive focus was placed on adapting our previous inpatient nutritional rehabilitation and medical stabilization protocol to incorporate more efficient practices and higher calorie levels while avoiding increased rates of complications. Changes also aimed to involve caregivers in the treatment process and effectively prepare them for their role in caring for their children in outpatient FBT. Protocol revisions and development were guided by the following principles: (1) incorporate evidence-based treatment whenever possible and otherwise utilize consensus-based guidelines, (2) utilize electronic order sets to improve adherence, (3) involve an iterative feedback process from staff and faculty who are involved in treatment, (4) understand that the protocol is a guideline for structured nutritional rehabilitation, but not a mandate or a substitute for clinical judgment, and (5) incorporate ongoing quality improvement and review of outcomes in a longitudinal manner, to understand how these protocols impact care over time.

**Fig. 1:**
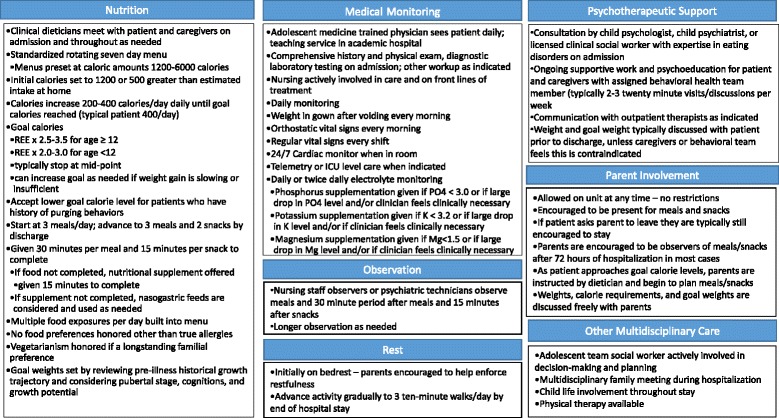
Protocol

Meetings with inpatient nursing staff occurred frequently during this first year, to gather feedback and further refine the protocol. These meetings also provided education and support around the challenges of introducing newer family-based philosophies for ED treatment on an inpatient adolescent medical unit as well as the effort involved in treating a higher volume of patients with these challenging disorders. A new standardized inpatient nutrition plan was introduced in January 2012 (see Additional file 1: Appendix A and Additional file 2: Appendix B). Our inpatient nutritional rehabilitation protocol underwent multiple revision cycles until a final version of our “Malnutrition Protocol” was introduced in July 2012 (Fig. 1).

### Protocol description

A description of all features of the CHOP Malnutrition Protocol is presented in Fig. 1. Some features are shared with many other nutritional rehabilitation programs, such as rest, electrolyte monitoring, gradual increases in nutrition and weight gain; more unique features will be described here.

Our standardized rotating menu has frequent intentional built-in food exposures (Additional file 1: Appendix A). Similar standardized prescriptions have been found to be helpful in accelerating weight gain in inpatients with AN [34]. The choice to introduce varied menus into the protocol was made after published studies suggested that lower variety diets may be associated with a higher risk of relapse [35, 36]. In most cases, initial caloric intake was selected based on the greater of a) 500 calories more than what the patient had been eating prior to admission or b) 1200 calories. The only exception to this rule was a five-year-old female who had eaten almost nothing for over a week prior to admission; she was started on 900 calories due to this extreme fasting and her young age. Unless there was a cogent medical reason to stop calorie advancement, the menus increased calories by 200–400 calories daily, a faster rate than what had been done prior to the implementation of the malnutrition protocol. To ensure that rates of refeeding syndrome did not increase, safety outcomes were followed carefully and reviewed frequently as part of an integrated quality improvement (QI) collection.

Patients with restrictive eating disorders are hypermetabolic during refeeding, and require higher-than-expected amounts of energy in order to maintain weight, let alone to achieve weight restoration [37, 38]. Thus, caloric goals to promote weight restoration at our institution were set based on the resting energy expenditure (REE) multiplied by a stress factor of 2.5 – 3.5, with the middle of the range selected for most patients. The REE was calculated utilizing equations published by the World Health Organization [39].

All patients received continuous cardiac monitoring on the adolescent medicine service. Patients were admitted for telemetry with constant provider monitoring of heart rhythm if they met any of the following criteria: 1) heart rate ≤ 35 beats per minute with no ventricular ectopic beats, 2) heart rate ≤ 40 beats per minute with simple ventricular ectopy, 3) complex ventricular ectopy including ventricular couplets or triplets, ventricular tachycardia, or atrioventricular block with any type of baseline heart rate, 4) QTc or QT interval >550 ms on baseline ECG if bradycardic, 5) QTc or QT interval >500 with normal or elevated heart rate or with known history of purging, 6) severe electrolyte abnormalities such as potassium <2.5, phosphorus <2.5, magnesium <1.5, or 7) recent unexplained syncope. If admitted to telemetry, the protocol was still initiated as otherwise described. For all admissions, electrolytes were monitored frequently (at least daily) upon admission and then less often after electrolytes stabilized. Hypokalemia was defined as potassium <3.2 mmol/L [40], hypophosphatemia was defined as phosphorus <3.0 mg/dL [1], and hypomagnesemia was defined as magnesium <1.5 mg/dL. The definition of hypomagnesemia was based on the laboratory reference range at our institution due to lack of a consensus definition in the literature.

Meals were served to patients at bedside, and nursing staff members were responsible for the implementation of the meal plan, as food was explained as ‘medicine’. Patients were given 30 minutes to complete meals and 15 for snacks; anything not eaten during that time was replaced with a nutritional supplement. In the early part of the inpatient stay, a member of our nursing staff (either a trained psychiatric technician or a sitter) was present for meals, snacks, and rest periods in the patient room, in addition to caregivers if present. As the stay progressed, parents were encouraged to take over responsibility for meal, snack, and rest period observations whenever possible. If patients refused nutrition by mouth, then a nasogastric (NG) tube was used as deemed necessary by the treatment team, based on medical severity and psychological considerations. NG feeds were not always implemented immediately, but if patients were repetitively refusing to eat, or very medically ill, they would be used. The nursing staff and parents were generally very effective in coaching patients to eat by mouth in this acute medical setting.

Our behavioral health team provides expert consultation to evaluate, diagnose, and give treatment recommendations for each patient, and helps support patients and families throughout the inpatient stay. In order to accomplish treatment goals on a busy adolescent medicine inpatient service, all providers – nurses, psychiatric technicians, physicians, therapists, dietitians, social workers, child life specialists, and more – were educated about FBT. In this manner, all members of the team were equipped to reinforce the basics of FBT theory to the family. Purposeful attempts were made by multiple team members to be clear to parents and caregivers that they were not to blame for the illness, and that they were their child or adolescent’s best allies in achieving recovery, irrespective of the type of treatment they elected at discharge. Parents and caregivers were welcomed at all meals, encouraged to stay even if there was conflict around eating and their child seemed upset, and had no limitations to their visitation hours. Parents were welcome to sleep in their child’s room and encouraged to read about EDs and their treatment. Parents were given regular updates about their child’s progress in weight and calories; all members of the multidisciplinary team worked together to reinforce psychoeducation around EDs and their management. The outpatient FBT program at our institution was explained to all families admitted for inpatient medical stabilization. Some families opted not to pursue FBT after discharge, citing reasons including, but not limited to, previously established outpatient team, desire to pursue a higher level of psychiatric care, distance from our institution, or family preference.

During the hospital stay, a focus was placed on helping parents to understand the importance of distress tolerance in treatment. The team worked to normalize the level of upset that patients experienced surrounding food exposures, volume, caloric density, and the necessity of weight gain during this phase. The fact that many children and adolescents with EDs do not ‘want’ to recover was explained and ways of supporting them toward health regardless of their motivation level were discussed.

Closer to discharge, parents received individualized nutrition education from a Registered Dietitian and were taught how to plan structured, calorie-dense meals that meet the recommended caloric prescription. Parents began to plan meals and order food for their children for at least 2 days prior to discharge. Caregivers were encouraged to offer a variety of different foods and to minimize categorizing certain foods as “good” or “bad.” Food from home or outside the hospital was permitted during this period and parents were encouraged to reintroduce foods their child used to eat pre-illness. Including parents in this way provides them with an opportunity for practice in successful exposures prior to discharge [30]. If patients did not gain appropriate weight with parental meal choices, meal choices and appropriate weight gain goals were discussed with the family. In addition, dietitians evaluated parental meal choices and provided feedback to the family regarding caloric needs.

Our protocol includes parents as part of our interdisciplinary team and uses the inpatient setting as an opportunity to educate families regarding appropriate food choices and meal supervision. When challenges such as families colluding with the eating disorder arose, the team addressed the problem directly with the family in the same way we would approach any learner. Appropriate redirection and education was provided to the family. In some cases, if clinically indicated, increased supervision of meals or selection of meals and snacks by medical personnel was instituted for a limited time to provide examples of appropriate supervision and food choices for the parents.

Behavioral health team members also spent time with both patient and caregivers reviewing expectations for time at home in the weeks immediately following discharge. During this time, which we named ‘home hospitalization,’ parents continued to oversee all food decisions and patients’ main priority is eating and resting. Gradual return to activity is determined collaboratively by families and behavioral health providers and is based on parental guidance and patient progress. Typical home hospitalization recommendations are in Additional file 2: Appendix B. Weight progress and treatment goal weights (TGWs, based on historical growth curves) were explained and shared with parents prior to discharge; in most cases this information was shared with patients as well. Caregivers were told to increase inpatient caloric prescriptions by 400 calories once home, as patients were typically slightly more active at home than on our inpatient unit.

Discharge criteria included resolution of medical instability, with heart rate >45 at night and >50 during the daytime, systolic blood pressure >90, temperature >36 at night and >36.3 during the daytime, resolution of orthostatic hypotension (change in BP <10 mm Hg from lying to standing), QTc <450 ms, and resolution of electrolyte abnormalities with no requirements for supplementation at discharge. In the majority of cases, daily intake was advanced to goal calorie level prior to discharge. For families participating in FBT, parents had an opportunity to choose and supervise meals in the hospital setting.

### Data collection

Continuous QI initiatives were built into the EDATP at program inception. An ongoing QI data collection focused on quality and safety outcomes was initiated in 2011. The QI project utilized retrospective chart review of the electronical medical record and is stored in REDCap, a HIPAA-secure web-based application designed for safe data repositories at larger institutions. Data from this QI database were used in this report.

### Population and target outcomes

All patients with EDs admitted to CHOP for a first-time stay for inpatient nutritional rehabilitation between October 2012 and October 2014 were included for review. Patients were admitted for medical criteria outlined in multiple pediatric position papers for the medical treatment of patients with EDs [1–3]. Common reasons for admission included bradycardia, hypotension, orthostasis, significant malnutrition (<75% median body mass index or MBMI), acute food refusal, failure to thrive, syncope, and electrolyte abnormalities. Patients were only included if they started the malnutrition protocol within 24 h of admission and stayed for at least 3 days. ED diagnoses were determined clinically and were DSM-IV based. After the publication of the DSM-5 in May of 2013 [41], all cases were reviewed and retrospectively reassigned DSM-5 diagnoses by members of the EDATP QI group. Prescribed calorie levels were recorded and compared with predicted REE as calculated by equations published by the World Health Organization [39]. Degree of malnutrition (mild, moderate, or severe) was defined based on the 2015 Consensus Statement of the Academy of Nutrition and Dietetics/American Society for Parenteral and Enteral Nutrition: Indicators Recommended for the Identification and Documentation of Pediatric Malnutrition (Undernutrition) and the 2015 Position Paper of Society for Adolescent Health and Medicine: Medical Management of Restrictive Eating Disorders in Adolescents and Young Adults classification of malnutrition [1, 42]. If the degree of malnutrition differed based on the two distinct sets of criteria, the higher degree of malnutrition was chosen.

**Fig. 2:**
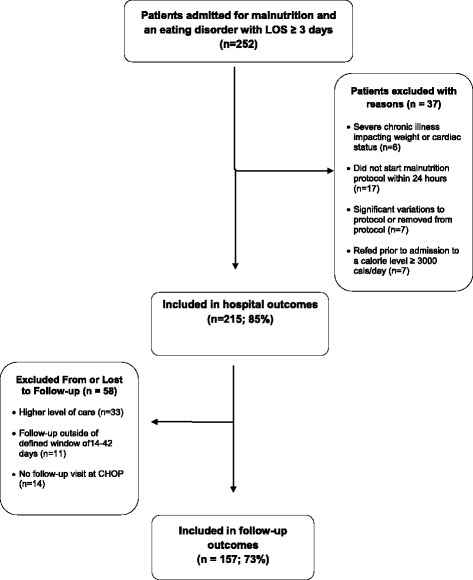
Inclusion/Exclusion chart

Four-week follow up data were included for patients seen in our outpatient EDATP between 14 and 42 days after hospital discharge. Figure 2 outlines all inclusion and exclusion criteria. Our safety outcomes were the clinical need for phosphorus, potassium, and magnesium supplementation, other evidence of refeeding syndrome, and unexpected readmissions within 1 month of discharge. Full threshold refeeding syndrome was defined as electrolyte derangements (phosphorus, potassium, and magnesium) with clinically observed acute circulatory fluid overload and organ dysfunction [17, 43]. Our value outcome was the length of stay (LOS). Treatment outcomes of interest were the percentage MBMI change from admission to discharge, and from discharge to 4-weeks follow-up visit. The CHOP Institutional Review Board determined this outcomes project was not research on human subjects and thus did not require IRB approval.

### Statistical analyses

Standard descriptive testing was used for reporting. Paired *t*-tests, ANOVA and repeated measures ANOVA testing were used to analyze target treatment outcomes.

## Results

### Clinical characteristics and medical severity

Clinical characteristics of patients on admission are described in Table 1. A total of 215 patients were included. Patients were mostly female (88%) and ranged in age from 5.8 years to 23.2 years of age (mean 15.3 years); 64% had AN, while 18% had atypical anorexia (AtAN), 6% bulimia nervosa (BN), 5% purging disorder (PD), 4% avoidant-restrictive food intake disorder (ARFID), and 3% had an unspecified food and eating disorder (UFED). Our average LOS was 11 days. A third of patients (35%) were taking psychotropic medication during their stay, with benzodiazepines and serotonin-specific reuptake inhibitors the most common; atypical antipsychotics were used in fewer than 10% of patients. The average initial calorie (kcal) level for patients who initiated the nutritional rehabilitation protocol was 1466. Average calories at discharge were approximately 3800 kcals/day; this was an average increase of 2288 calories per stay. Only 10% of inpatients received any NG feeds during their admission. ARFID patients were more likely to require NG feeds than patients with other DSM-5 diagnoses (23 vs 8%, *p* <0.001), and patients requiring NG feeds were younger than those who did not require NG supplementation (12 years vs 16 years, *p* <0.001).

**Table 1:** Clinical characteristics of patients

	*N*	Percent	Min	Max	*M*	*SD*
Admission and hospitalization						
Gender						
*Male*	25	12				
*Female*	190	88				
Ethnicity						
*Hispanic or Latino*	5	2				
*Not Hispanic or Latino*	210	98				
Race						
*White*	185	86				
*Black or African American*	10	5				
*Asian*	5	2				
*Other*	15	7				
Diagnosis (DSM-5)						
*Anorexia Nervosa (AN)*	138	64				
*Bulimia Nervosa (BN)*	12	6				
*Avoidant-Restrictive Food Intake Disorder (ARFID)*	9	4				
*Atypical anorexia*	38	18				
*Purging disorder*	10	5				
*Unspecified Food and Eating Disorder (UFED)*	8	3				
Age, Years	215		5.8	23.2	15.3	2.8
Duration illness, months	215		0.7	115.4	18.8	21.4
Height, cm	215		112.3	179.0	159.3	11.5
Weight, kg	215		17.2	107.2	44.1	11.5
BMI, kg/m^2^	215		11.2	35.4	17.1	3.1
BMI Z-Score	215		−10.1	2.2	−1.6	1.5
Degree of malnutrition						
*Mild malnutrition*	11	5				
*Moderate malnutrition*	23	11				
*Severe malnutrition*	181	84				
Percentage Median BMI (%MBMI)	215		52.5	153.7	86.1	13.9
Treatment goal weight (TGW: Projected growth curves)	215		19.0	85.6	54.0	11.2
Percentage TGW	215		47.9	131.2	81.2	9.6
Length of stay, days	215		3	40	11	5
Length on telemetry/ICU, days	18		1	9	3	3
Resting energy expenditure	215		872	2235	1279	174
Admission calories	215		900	2800	1466	399
Admission calories/REE	215		0.54	2.39	1.16	0.34
Discharge calories	215		1800	5600	3754	672
Discharge calories/REE	215		1.07	4.37	2.96	0.50
Rate of calorie change, kcals/day	215		25	520	223	75
Admission - discharge calorie change	215		200	4400	2288	712
Admission- discharge calorie change/REE	215		0.14	3.28	1.79	0.51
Required nasogastric feeds	22	10				
On psychotropic meds during stay	75	35				
*Benzodiazepines*	25	11.6				
*SSRI’s*	26	12.1				
*Atypical antipsychotiics*	20	9.3				
*SNRI*	6	2.7				
*Mood stabilizers (anticonvulsants)*	5	2.3				
*Alpha agonists*	4	1.8				
*Tricyclic antidepressants*	3	1.4				
*Stimulants*	1	0.4				
*Other (Mirtazapine, Buspirone, Trazodone)*	7	3.2				
Supplementation required						
*Phosphorus*	31	14				
*Potassium*	9	4				
*Magnesium*	6	3				
Percentage transferred to a higher level of care	32	14.9				
Discharge						
Discharge BMI	215		12.8	33.8	18.2	2.9
Discharge %MBMI	215		60.1	146.8	91.4	13.6
Discharge %TGW	215		58.1	125.3	86.1	8.9
Percentage MBMI Gain from Admission	215		−7.1	21.9	5.3	3.7
Percentage TGW Gain from Admission	215		−5.9	22.1	5.0	3.5
Weight Gain, kg, from Admission	215		−4.8	6.7	2.5	1.7
Average daily weight gain, kg	215		−0.96	0.57	0.22	0.16
Follow Up						
Percentage rehospitalized						
*Within 30 days*	6	3.8				
*Within 1 year*	37	17.2				
4 Weeks Follow-up BMI	157		13.6	34.2	20.0	2.8
4 Weeks Follow-up %MBMI	157		66.1	148.6	100.9	12.9
4 Weeks Follow-up %TGW	157		65.4	126.0	94.9	9.4
Percentage MBMI Gain from Discharge	157		−12.8	25.4	9.2	6.0
Percentage TGW Gain from Discharge	157		−13.5	23.9	8.8	5.7
Weight Gain, kg, from Discharge	157		−6.7	11.9	4.6	3.0

Medical severity criteria met by this group of inpatients on admission is presented in Table 2. Patients met these criteria for admission as expected, with 20% being malnourished below 75% MBMI, 35% bradycardic, 15% hypotensive, and nearly 53% orthostatic on admission. The majority of patients (84.2%) met criteria for severe malnutrition.

**Table 2:** Medical severity at admission

	*N*	Percent	Mean	SD	Min	Max
MBMI <75%	215	20.0				
Heart Rate (HR), lying, beats per minute	215		61	17	32	112
Percentage bradycardia (HR <50 beats per minute)	75	34.9				
Percentage Orthostasis by Heart rate (increase of >20 beats per minute when standing)	95	52.6				
Systolic Blood Pressure, lying, mm Hg	215		101	12	65	137
Systolic Blood Pressure <90, mm Hg	32	14.9				
Percentage Orthostasis by Blood Pressure (drop in SBP by >10 mm Hg when standing)	22	12.1				
Temperature, Celsius	205		36.8	0.3	36.1	38.6
Percentage Hypothermia (temp <36.3 C)	205	2.3				
EKG QTc Interval, ms	189		409.6	26.3	337	485
Percentage QTc Prolongation >440 ms	189	8.4				
Potassium, mmol/L	215		4.2	0.9	2.7	12.6
Percentage hypokalemia <3.2 mmol/L	4	1.9				
Phosphorus, mg/dL	215		4.2	0.7	1.7	6.6
Percentage hypophosphatemia <3.0 mg/dL	9	4.2				
Magnesium, mg/dL	214		2.1	0.4	1.5	6.0
Percentage hypomagnesemia <1.5 mg/dL	0	0				
ALT, U/L	169		36	31	6	297
Percentage high ALT >44 U/L	29	17.1				

### Safety outcomes

A small number (8%) of patients needed a telemetry or intensive care level of cardiac monitoring initially in their stay due to severe bradycardia or electrolyte instability on admission. During the clinical stay, phosphorus supplementation was prescribed for refeeding hypophosphatemia (RH) for 14% of patients, potassium supplementation for 4% of patients, and magnesium supplementation for 3% of patients. No patients experienced full-threshold refeeding syndrome. Fewer than 15% of patients were transferred to a higher level of psychiatric care at the end of their inpatient stay. Only 3.8% of patients were readmitted within 30 days of discharge.

### Weight outcomes

Patients averaged 86% MBMI for age and gender, and 81% of a TGW determined as a clinical goal weight based on historical growth curves. Patients gained an average of 2.5 kg during their stay, achieving 91% MBMI at discharge. Nearly three-quarters of included inpatients followed up 4 weeks after discharge (mean 28.5 days, range 15–41 days); they had achieved 101% MBMI by that time point.

**Fig. 3:**
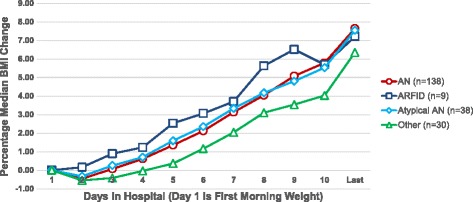
Daily weight chart

Cumulative percentage MBMI (%MBMI) gained during the hospital stay, stratified by DSM-5 diagnostic category, is presented in Fig. 3. Daily %MBMI change is presented daily through Day 10, and then the last day of %MBMI change is given for those patients who stayed 11 days or more. Patients with AN had gained statistically significant %MBMI above baseline by Day 3 of the hospitalization. Patients with ARFID, atypical AN, and other eating disorder diagnoses did not demonstrate a statistically significant %MBMI gain from baseline until later in their hospital stay, however these groups also had small sample sizes. All groups gained 6–8% MBMI cumulatively over the course of their hospitalization, with an average LOS of 11 days. Patients with AN, AtAN, and ARFID gained more %MBMI during their hospital stay than those with other DSM-5 diagnoses, despite similar lengths of stay. As patients are typically admitted to CHOP later in the day or overnight, Day 1 on this graph represents the weight obtained the first morning of the hospitalization. We noted that patients ‘lost’ on average 0.7 kg (SD 0.9) by this morning, after just hours in the emergency room or on the hospital floor; the largest loss recorded was 4.3 kg. Losses were greatest in patients with AN or AtAN. Clinically we believed this due to intravenous fluid administration in the emergency room setting or procedures patients with EDs often employ to artificially increase their weight in the outpatient setting, such as using actual hidden weights or drinking excessive amounts of fluids prior to getting weighed on the scale. Because of this finding, we started our graph with the baseline of the first morning weight, still within 24 h of admission.

**Fig. 4:**
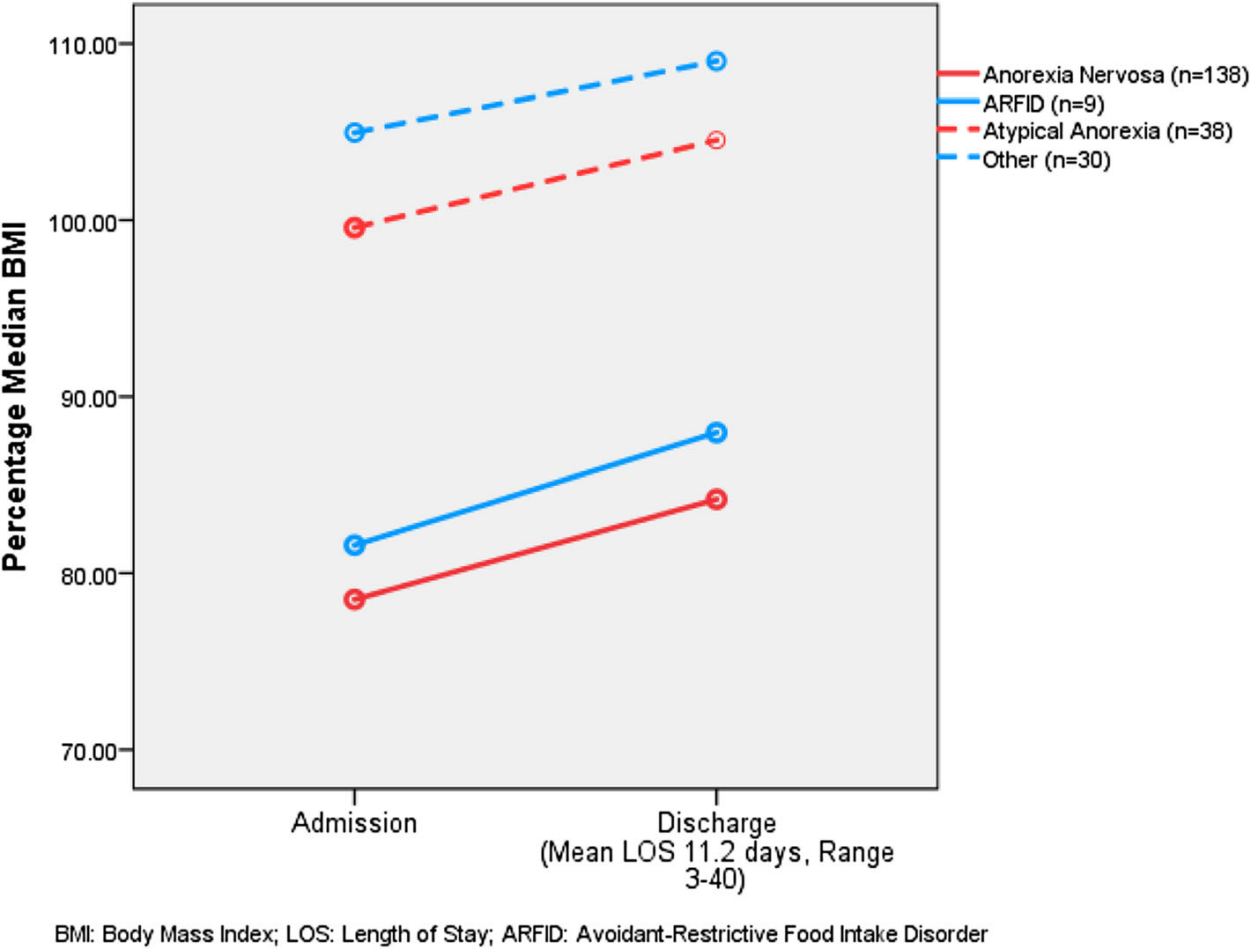
Percentage MBMI change during hospitalization

**Fig. 5:**
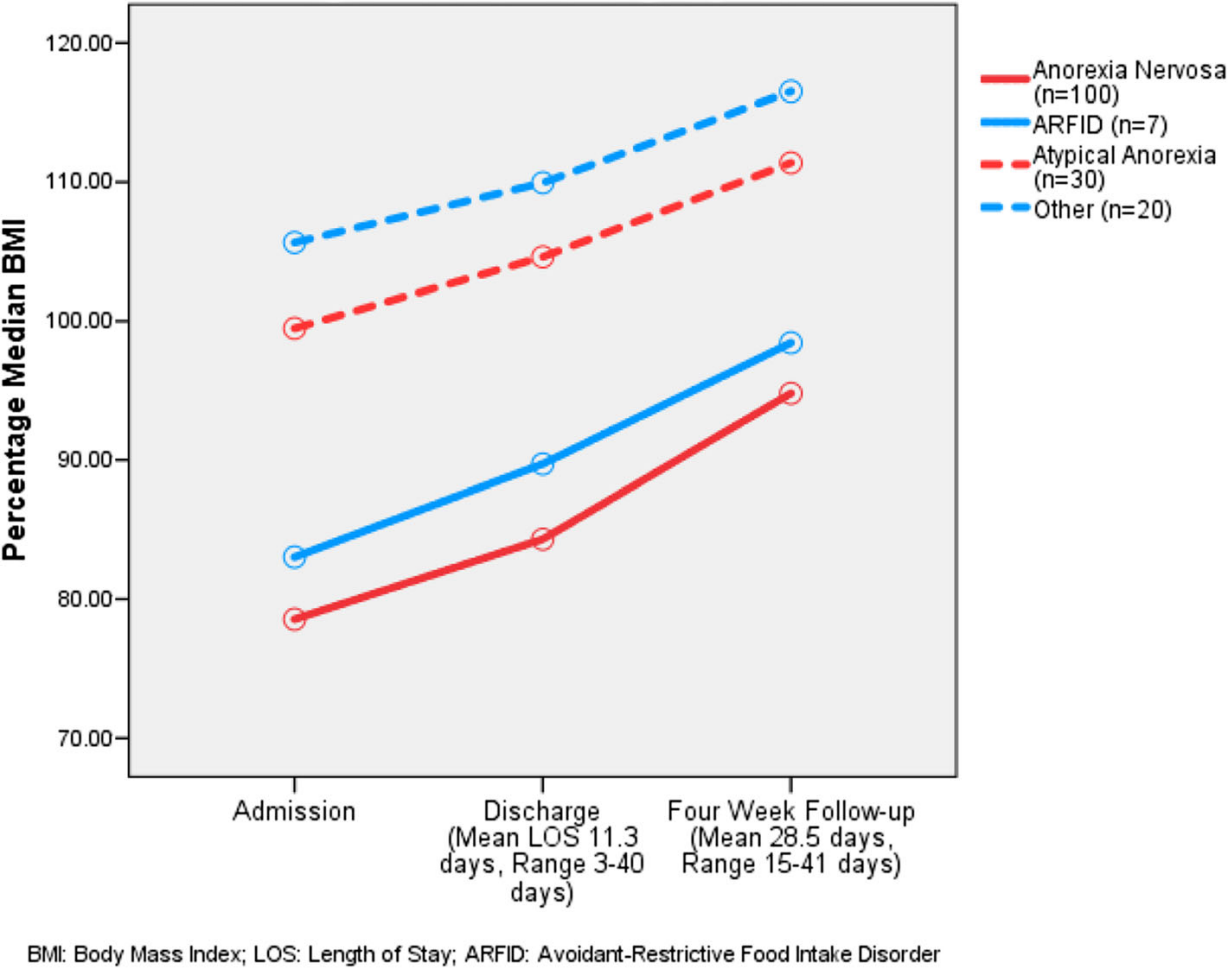
Percentage MBMI change by follow-up

Figures 4 and 5 represent the average %MBMI on admission, discharge (Fig. 4), and then these same time-points and 4-weeks follow-up for the 73% that had follow-up at the EDATP in the designated time window of 14–42 days (Fig. 5). Mean time to follow up was 28 days (range 15 – 51 days). Note that patients with AN and ARFID started at lower %MBMI than those with AtAN or other diagnoses (AN: 78.6 ± 7.0; ARFID 81.6 ± 6.5; AtAN 99.6 ± 6.3; Other 105.0 ± 15.5; *p* <0.001), and these relative differences persisted at discharge (AN: 84.2 ± 7.4; ARFID 88.0 ± 7.6; AtAN 104.5 ± 7.1; Other 109.0 ± 14.9; *p* <0.001) and follow-up (AN 94.4 ± 8.8; ARFID 98.4 ± 9.5; AtAN 110.6 ± 8.2; Other 114.9 ± 13.9; *p* <0.001). Mean percentage MBMI differences between time points were significantly different (admission-discharge: 5.3%, *p* <0.001; discharge-follow-up: 9.2%, *p* <0.001). 58.5% of patients seen at follow-up were engaged in FBT outpatient; the rest were either not in psychotherapeutic care or seeing non-FBT providers. No significant differences were noted in %MBMI outcomes for patients with AN who were or were not in FBT-based care; comparisons were not performed in other diagnostic categories due to limited sample sizes.

## Discussion

The nutritional rehabilitation protocol introduced at CHOP in 2012 was effective in achieving excellent %MBMI gains while inpatient and at 4-weeks follow-up. Our program had a short mean LOS, low rates of RH phosphorus supplementation, and few readmissions within 30 days. Outcomes were achieved with relatively low rates of NG feedings and psychotropic medication use. Most patients did not require a higher level of psychiatric care at discharge or follow-up. On admission, patients were similarly medically compromised as in other larger studies of adolescent inpatients with EDs, with 84% severely malnourished [44]. Our LOS was shorter and %MBMI increase was greater for patients with AN than reported in most prior studies [22]. Results achieved inpatient were sustained outpatient; on average, patients actually gained more %MBMI after discharge than during the hospital stay. This is the first detailed report on a nutritional rehabilitation protocol treating all types of EDs for medical stabilization in a general medical inpatient setting, while integrating and preparing parents for FBT after discharge.

Achieving an average increase of 2388 calories over a mean LOS of 11 days was well tolerated and safe using a standardized rotating menu with built-in food exposures. Pre-implementation concerns regarding acceptance of standardized meal plans by families were quickly alleviated; the menu was typically well tolerated by patients and much appreciated by caregivers. Eliminating patient involvement in food selection allowed for a smoother transition to home where parents are responsible for decisions around activities and food. Our safety outcomes demonstrated low rates of RH phosphorus supplementation than most rates previously reported [20]. There were no incidences of full-threshold refeeding syndrome. This is consistent with results from multiple other studies showing no increases in refeeding syndrome when more aggressive refeeding regimens are introduced. It is also noteworthy that most other studies that achieved the degree of weight gain reported here utilized enteral feeding regimens more frequently [11–22]. Consistent with prior studies, patients requiring enteral feeds at CHOP were younger and more likely to have ARFID than patients who did not require supplemental NG feeds [45].

In addition to sustained weight gain in the outpatient setting, patients demonstrated success after discharge, with only a small proportion of patients requiring rehospitalization within the first 30 days after discharge. Rates of rehospitalization at our institution within 1 year after discharge are similar or less than those reported in other studies [45, 46]. Readmission rates must be cautiously interpreted as patients can drop in and out of care, and may have (without our knowledge) been hospitalized or required a higher level of care outside of our institution.

One unique component to our protocol is that it provided a structured way to integrate caregivers into routine care on a busy inpatient medical ward. This helped allow parents to work on distress tolerance organically throughout the inpatient stay, better preparing both caregivers and patients for challenges that will occur at home. All members of the team were equipped to reinforce common FBT messaging in small ‘sound bites’ throughout the stay in lieu of a more programmed psychiatric treatment milieu. It is noteworthy that our protocol does not involve intensive family or individual therapy sessions, ED-focused therapy groups, or other ED-specific ward programming, aside from the behavioral health consultation, support, and psychoeducation described. While nearly 60% of patients and their families were in FBT at follow-up, it is important to note that those who had AN but were not in FBT at follow-up did equally well. While this report cannot state causality, it is plausible to infer that the practice of involving parents in all levels of care, but particularly during the hospital stay and outlining a home hospitalization method after discharge translates well to success in early outpatient follow-up. Studies have shown that gaining 2.88% MBMI in the first 4 weeks of FBT is a strong predictor of future success in FBT; most of our patients were able to far exceed this threshold by 4 weeks. Clearly patients with EDs continue to need care far beyond a period of weight restoration, typically for at least a year, and the necessity of high-quality outpatient care teams remains for our outpatients. However, the benefits of rapid, early weight gain while in hospital cannot be denied. Future research needs to elucidate whether achieving earlier and more rapid weight restoration using a brief but integrated inpatient stay is beneficial to long-term outcomes and/or helps avoid more costly levels of care.

Our protocol was designed to serve all patients with malnutrition due to EDs, irrespective of weight status or diagnosis by DSM. Recent literature on weight suppression and AtAN has informed our program’s stance that even patients at or near 100% MBMI will likely need to gain weight for their ED cognitions to resolve [25, 47–52]. We promote a non weight-biased approach to treatment, in which we aim for patients to achieve previous growth trajectories as determined by historical growth charts unless they were extremely overweight or underweight pre-illness. In the case of extreme premorbid weights, we are clear that a median BMI is likely not an appropriate goal, but it is also unlikely that we will need to return to prior ends of the growth curve to achieve psychological remission. Instead, while we base initial TGWs on previous growth trajectories, we closely follow and base final weight endpoints on tangible goals of wellness in physical, pubertal, and cognitive domains. Our protocol is designed to achieve early weight gain as necessary for patients of all weights and diagnoses, and then to normalize to a TGW over time as an outpatient. Recovery is achieved when a person has achieved a total picture of health – a “state” rather than a specific weight [53].

There were certainly some hurdles in implementing the Malnutrition Protocol at CHOP. First and foremost, the protocol and the overall care of patients with EDs demands a significant amount of bedside care from nursing staff on a busy medical unit. We rely on our nurses, psychiatric technicians, and sitters to help patients get the critical nutrition they need to recover, and to help integrate parents into the meal process. While our nurses are excellent at providing adolescent-specific care, they are primarily medically trained, and helping patients and parents behaviorally during an initial hospitalization can be draining at times. It has been particularly meaningful to our inpatient team, as they do not get to see outpatient progress after discharge, to have patients come back to the ward when healthy, email or send notes after they recover, or have their parents do the same. We had regularly scheduled process groups available for nurses to express frustrations and ask questions during the first year of the protocol, and have resumed these again now in 2016. Our physicians make a particular effort to involve nursing providers in their daily treatment rounds, so that they both contribute to the plan and are adequately informed to be at the bedside throughout the day.

This approach can be counterintuitive and challenging for parents and providers, who often mistakenly believe a more traditional setting would be preferable and hope that if a provider can talk to their child in the right way at the right time they will agree to eat well and will no longer be in distress. While we endeavor to be therapeutic, we do have to be boundaried about the amount of time that inpatient staff can dedicate to long conversations – particularly because the likelihood of such conversations reducing distress in such an acute setting is low. In addition, such discussions can even undermine future outpatient behavioral paradigms. Instead, our focus is on helping parents and staff alike to normalize and tolerate this distress.

Finally, goal weights, calorie levels, and recommended breaks in schooling may be changed if patients are discharged to non-FBT teams. Reasons for this vary, but in our experience outpatient providers can be tempted to reverse treatment recommendations in order to reduce anxiety in their patients. In addition, other behavioral approaches may not focus on early weight gain, and may advocate for lower caloric prescriptions and slower increases over time. Weight biases of our own staff at CHOP, parents, and of community providers are encountered at times, and need to be addressed when discussing TGWs and what is ‘normal’ for an individual patient. Keeping open lines of communication is critical to success when we collaborate in care systems that may be different than our own.

## Conclusions

Implementation of the CHOP inpatient nutritional rehabilitation protocol aimed at rapid, efficient, and safe weight gain and integration of caregivers in treatment of patients with diverse ED diagnoses led to excellent outcomes in %MBMI, while maintaining a short LOS and low rates of RH supplementation. These outcomes were achieved on a busy inpatient adolescent medicine unit rather than an inpatient psychiatric treatment setting. Short-term outpatient outcomes were similarly positive. Future research and QI initiatives should focus on how best to sustain and build on these gains to achieve long-term recovery in treatment of adolescents and children faced with these serious but treatable illnesses.

## Additional files

Additional file 1: Appendix A. Malnutrition Pathway meal plans and menu modifications. (PDF 1006 kb)
Additional file 2: Appendix B. Nutrition education on inpatient meal plan and home hospitalization. (PDF 197 kb)

## Abbreviations

%MBMI: Percentage median body mass index
AN: Anorexia nervosa
ARFID: Avoidant restrictive food intake disorder
AtAN: Atypical anorexia
BMI: Body mass index
BN: Bulimia nervosa
CHOP: The children’s hospital of Philadelphia
DSM: Diagnostic and statistical manual
ED: Eating disorder
EDATP: Eating disorder assessment and treatment program
FBT: Family-based treatment
LOS: Length of stay
MBMI: Median body mass index
NG: Nasogastric
PD: Purging disorder
QI: Quality improvement
REE: Resting energy expenditure
RH: Refeeding hypophosphatemia
TGW: Treatment goal weight
UFED: Unspecified feeding and eating disorder
