# Supplementary material for: Outcomes of an inpatient medical nutritional rehabilitation protocol in children and adolescents with eating disorders
Source: J Eat Disord. 2017 Mar 1;5:7. doi: 10.1186/s40337-017-0134-6 (PMC5331684; doi:10.1186/s40337-017-0134-6)
Supplement: Additional file 1: Appendix A. — Malnutrition Pathway meal plans and menu modifications. (PDF 1006 kb) [file 40337_2017_134_MOESM1_ESM.pdf]

1200 kcal Malnutrition Pathway Meal Plan

MONDAY	TUESDAY	WEDNESDAY	THURSDAY	FRIDAY	SATURDAY	SUNDAY
**Breakfast**						
8oz 2% milk (130)	8oz 2% milk (130)	8oz 2% milk (130)	8oz 2% milk (130)	8oz 2% milk (130)	8oz 2% milk (130)	8oz 2% milk (130)
3/4 C oatmeal (110)	breakfast sandwich (1 oz egg on english muffin) (165)	half bagel (120)	3 French toast sticks (195) with syrup (110)	2 oz scrambled eggs (90)	1 pancake (100) with half packet syrup (55)	2oz egg omelet (90)
2 oz raisins (130)	banana (110)	cream cheese (100)	1 link turkey sausage (70)	1 cup strawberries (60)		1/2 cup honeydew (30)
light strawberry yogurt (80)		blueberry yogurt (120)				
**450**	**405**	**470**	**505**	**280**	**285**	**250**
**Lunch**						
8oz apple juice (120)	8oz fruit punch (120)	8oz ginger ale (80)	8oz orange juice (120)	8oz grape juice (170)	8oz lemonade (120)	8oz cranberry juice (120)
Grilled Tilapia with Butter and Herbs, 3.5 oz (130)	4 oz spiral pasta (210) with 1/4 cup marinara sauce (25)	half hamburger (190) offered with ketchup, lettuce, tomato, pickle	Macaroni and Cheese, 4 oz (230)	half grilled cheese sandwich (250)	tuna salad sandwich on wheat (340)	turkey (3 oz) sandwich on wheat with mayo (360)
1/2 cup pineapple (70)			1/2 cup green beans (35)	garden salad (15) with ranch dressing (140)		half apple, sliced (40)
1/4 cup steamed rice (70)						
**390**	**355**	**270**	**385**	**575**	**460**	**520**
**Dinner**						
8oz 2% milk (130)	8oz 2% milk (130)	8oz 2% milk (130)	8oz 2% milk (130)	8 oz Wawa iced tea (90)	8oz 2% milk (130)	8oz 2% milk (130)
grilled chicken breast, sliced (140)	3 oz roast turkey (185)	chicken alfredo with 3 oz chicken, 3 oz pasta, 2 oz sauce (320)	beef fajitas (255)	half pepperoni pizza (280)	Chicken LoMein with Veggies (370)	2 chicken tenders (200) offered with 1 packet ketchup
1/2 cup cantaloupe (40)	1/2 cup mashed potatoes (130)	1/2 cup broccoli (20)				1/2 cup french fries (190) offered with 1 packet ketchup
1/2 cup raw carrot sticks (35)	1/2 cup broccoli (20)					
**345**	**465**	**470**	**385**	**370**	**500**	**520**
**1185**	**1225**	**1210**	**1275**	**1225**	**1245**	**1290**

Meals = 350-450 calories (1.75 cans Boost)

1200 kcal Malnutrition Pathway Meal Plan

**720**	**720**	**720**	**720**	**720**	**720**	**720**

Meals = 350-450 calories (1.75 cans Boost)

1400 kcal Malnutrition Pathway Meal Plan

MONDAY	TUESDAY	WEDNESDAY	THURSDAY	FRIDAY	SATURDAY	SUNDAY
**Breakfast**						
8oz 2% milk (130)	8oz 2% milk (130)	8oz 2% milk (130)	8oz 2% milk (130)	8oz 2% milk (130)	8oz 2% milk (130)	8oz 2% milk (130)
3/4 cup oatmeal (110)	breakfast sandwich (1 oz egg on english muffin) (165)	half bagel (120)	4 French toast sticks (260) with syrup (110)	3 oz scrambled eggs (135)	2 pancakes (200) with syrup (110) and pat butter (35)	2oz egg omelet (90)
2 oz raisins (130)	banana (110)	cream cheese (100)	1 link turkey sausage (70)	1 cup strawberries (60)		1/2 cup honeydew (30)
light strawberry yogurt (80)		blueberry yogurt (150)				1 slice wheat toast (70) with 1 pat butter (35)
**450**	**405**	**500**	**570**	**325**	**475**	**355**
**Lunch**						
8oz apple juice (120)	8oz fruit punch (120)	8oz ginger ale (80)	8oz orange juice (120)	8oz grape juice (170)	8oz lemonade (120)	8oz cranberry juice (120)
Grilled Tilapia with Butter and Herbs, 3.5 oz (130)	6 oz spiral pasta (315) with 1/3 cup marinara sauce (35)	hamburger (375) offered with ketchup, lettuce, tomato, pickle	Macaroni and Cheese, 6 oz (340)	half grilled cheese (250)	tuna salad sandwich on wheat (340)	turkey (3 oz) sandwich on wheat with mayo (360)
1/2 cup pineapple (70)	1 meatball (100)		1/2 cup green beans (35)	garden salad (15) with ranch dressing (140)		half apple, sliced (40)
1/4 cup steamed rice with 1 pat butter (105)				fresh fruit cup (50)		
side caesar salad with 1/2 pack ceasar dressing (185)						
**610**	**570**	**455**	**495**	**625**	**460**	**520**

Meals = 450-550 calories (2 cans Boost)

1400 kcal Malnutrition Pathway Meal Plan

Dinner						
8oz 2% milk (130)	8oz 2% milk (130)	8oz 2% milk (130)	8oz 2% milk (130)	8 oz Wawa iced tea (90)	8oz 2% milk (130)	8oz 2% milk (130)
grilled chicken breast, sliced (140)	3 oz roast turkey (185)	chicken alfredo with 3 oz chicken, 3 oz pasta, 2 oz sauce (320)	beef fajitas (255)	1/2 pepperoni pizza (280)	Chicken LoMein with Veggies (370)	2 chicken tenders (200) offered with 1 packet ketchup
1/2 cup cantaloupe (40)	1/2 cup mashed potatoes (130)	1/2 cup broccoli (20)		1 cup grapes (60)		1/2 cup french fries (190)
1/2 cup raw carrot sticks (35)	1/2 cup broccoli (20)			1/2 cup broccoli (20)		
**345**	**465**	**470**	**385**	**450**	**500**	**520**
**Total Calories: 1405**	**1440**	**1425**	**1450**	**1400**	**1435**	**1395**
**Total Fluid: 720 mL**	**720**	**720**	**720**	**720**	**720**	**720**

Meals = 450-550 calories (2 cans Boost)

1600 kcal Malnutrition Pathway Meal Plan

MONDAY	TUESDAY	WEDNESDAY	THURSDAY	FRIDAY	SATURDAY	SUNDAY
**Breakfast**						
8oz 2% milk (130)	8oz 2% milk (130)	8oz 2% milk (130)	8oz 2% milk (130)	8oz 2% milk (130)	8oz 2% milk (130)	8oz 2% milk (130)
3/4 cup oatmeal (110)	breakfast sandwich (1 oz egg on english muffin with 1 oz American cheese) (240)	bagel (240)	4 French toast sticks (260) with syrup (110) and pat butter (35)	3 oz scrambled eggs (135) with 1 oz cheese (75)	2 pancakes (200) with syrup (110) and 2 pats butter (70)	2oz egg omelet with 0.5 oz American cheese (130)
2 oz raisins (130)	banana (110)	cream cheese (100)	1 link turkey sausage (70)	1 cup strawberries (60)	turkey bacon, 2 oz (70)	1/2 cup honeydew (30)
light strawberry yogurt (80)		blueberry yogurt (150)	1/2 cup pineapple (70)			2 slices wheat toast (140) with 2 pats butter (70)
**450**	**480**	**620**	**675**	**400**	**580**	**500**
**Lunch**						
8oz apple juice (120)	8oz fruit punch (120)	8oz ginger ale (80)	8oz orange juice (120)	8oz grape juice (170)	8oz lemonade (120)	8oz cranberry juice (120)
Grilled Tilapia with Butter and Herbs, 3.5 oz (130)	8 oz spiral pasta (420) with 1/2 cup marinara sauce (50)	cheeseburger, 1 oz cheese (450) offered with ketchup, lettuce, tomato, pickle	Macaroni and Cheese, 6 oz (340)	half grilled cheese (250)	tuna salad sandwich on wheat (340)	turkey (3 oz) sandwich on wheat with mayo (360)
1/2 cup pineapple (70)	1 meatball (100)		1/2 cup green beans (35)	garden salad (15) with ranch dressing (140)	garden side salad (15)	whole apple, sliced (80)
potato stars, 2 oz (180)				fresh fruit cup (50)	Italian dressing (110)	1/2 cup broccoli (20)
side caesar salad with ceasar dressing (305)						
**805**	**690**	**530**	**495**	**625**	**585**	**580**
**Dinner**						

Meals = 500-600 calories (2.25 cans Boost)

1600 kcal Malnutrition Pathway Meal Plan

8oz 2% milk (130)	8oz 2% milk (130)	8oz 2% milk (130)	8oz 2% milk (130)	8 oz Wawa iced tea (90)	8oz 2% milk (130)	8oz 2% milk (130)
grilled chicken breast, sliced (140)	3 oz roast turkey (185)	chicken alfredo with 3 oz chicken, 3 oz pasta, 2 oz sauce (320)	beef fajitas (255)	3/4 pepperoni pizza (420)	Chicken LoMein with Veggies (370)	3 chicken tenders (300) offered with 1 packet ketchup
1/2 cup cantaloupe (40)	1/2 cup mashed potatoes (130)	1/2 cup broccoli (20)	1/2 cup cooked carrots (35)	1 cup grapes (60)		1/2 cup french fries (190)
1/2 cup raw carrot sticks (35)	1/2 cup broccoli (20)		1/2 cup honeydew (30)	1/2 cup broccoli (20)		
**345**	**465**	**470**	**450**	**590**	**500**	**550**
**Total Calories: 1600**	**1635**	**1620**	**1620**	**1615**	**1665**	**1630**
**Total Fluid: 720 mL**	**720**	**720**	**720**	**720**	**720**	**720**

Meals = 500-600 calories (2.25 cans Boost)

1800 kcal Malnutrition Pathway Meal Plan

MONDAY	TUESDAY	WEDNESDAY	THURSDAY	FRIDAY	SATURDAY	SUNDAY
**Breakfast**						
8oz 2% milk (130)	8oz 2% milk (130)	8oz 2% milk (130)	8oz 2% milk (130)	8oz 2% milk (130)	8oz 2% milk (130)	8oz 2% milk (130)
1c oatmeal (145)	breakfast sandwich (1 oz egg on english muffin with 1 oz American cheese) (240)	bagel (240)	4 French toast sticks (260) with syrup (110) and pat butter (35)	3 oz scrambled eggs (135) with 1 oz cheese (75)	2 pancakes (200) with syrup (110) and 2 pats butter (70)	2oz egg omelet with 0.5 oz American cheese (130)
2 oz raisins (130)	banana (110)	cream cheese (100)	1 link turkey sausage (70)	1 cup strawberries (60)	turkey bacon, 2 oz (70)	1/2 cup honeydew (30)
light strawberry yogurt (80)		blueberry yogurt (150)	1/2 cup pineapple (70)			2 slices wheat toast (140) with 2 pats butter (70)
**485**	**480**	**620**	**675**	**400**	**580**	**500**
**Lunch**						
8oz apple juice (120)	8oz fruit punch (120)	8oz ginger ale (80)	8oz orange juice (120)	8oz grape juice (170)	8oz lemonade (120)	8oz cranberry juice (120)
Grilled Tilapia with Butter and Herbs, 3.5 oz (130)	8 oz spiral pasta (420) with 1/2 cup marinara sauce (50)	cheeseburger, 1 oz cheese (450) offered with ketchup, lettuce, tomato, pickle	Macaroni and Cheese, 8 oz (460)	half grilled cheese (250)	tuna salad sandwich on wheat (340)	turkey (3 oz) sandwich on wheat with mayo (360)
1/2 cup pineapple (70)	2 meatball (200)		1/2 cup green beans (35)	garden salad (15) with ranch dressing (140)	garden side salad (15)	whole apple, sliced (80)
potato stars, 3 oz (270)				fresh fruit cup (50)	Italian dressing (110)	1/2 cup broccoli (20)
side caesar salad with ceasar dressing (305)						
**895**	**790**	**530**	**615**	**625**	**585**	**580**
**Dinner**						
8oz 2% milk (130)	8oz 2% milk (130)	8oz 2% milk (130)	8oz 2% milk (130)	8 oz Wawa iced tea (90)	8oz 2% milk (130)	8oz 2% milk (130)

Meals = 550-650 calories (2.5 cans Boost)

1800 kcal Malnutrition Pathway Meal Plan

grilled chicken breast, sliced (140)	3 oz roast turkey with 3 oz gravy (225)	chicken alfredo with 3 oz chicken, 3 oz pasta, 2 oz sauce (320)	beef fajitas (255) with 0.5 oz shredded cheddar cheese (60)	pepperoni pizza (560)	Chicken LoMein with Veggies (370)	3 chicken tenders (300) offered with 1 packet ketchup
1/2 cup cantaloupe (40)	1/2 cup mashed potatoes (130)	1/2 cup broccoli (20)	1/2 cup cooked carrots (35) with pat butter (35)	1 cup grapes (60)	Brownie cupcake (185)	3/4 cup french fries (285)
1/2 cup raw carrot sticks (35)	1/2 cup broccoli with pat of butter (55)	6 oz Cookies & Cream Hershey© ice cream cup (250)	1/2 cup honeydew (30)	1/2 cup broccoli (20) with pat butter (35)		1/2 cup green beans (35) with pat butter (35)
1/4 cup steamed rice (70)	1/2 cup peaches (50)					
**415**	**590**	**750**	**545**	**765**	**685**	**785**
**Total Calories: 1795**	**1860**	**1900**	**1800**	**1790**	**1850**	**1865**
**Total Fluid: 720 mL**	**720**	**720**	**720**	**720**	**720**	**720**

Meals = 550-650 calories (2.5 cans Boost)

2000 kcal Malnutrition Pathway Meal Plan

MONDAY	TUESDAY	WEDNESDAY	THURSDAY	FRIDAY	SATURDAY	SUNDAY
**Breakfast**						
8oz whole milk (150)	8oz whole milk (150)	8oz whole milk (150)	8oz 2% milk (130)	8oz 2% milk (130)	8oz whole milk (150)	8oz whole milk (150)
1c oatmeal (145)	breakfast sandwich (1 oz egg on english muffin with 1 oz American cheese) (240)	bagel (240)	4 French toast sticks (260) with syrup (110) and pat butter (35)	3 oz scrambled eggs (135) with 1 oz cheese (75)	2 pancakes (200) with syrup (110) and 2 pats butter (70)	2oz egg omelet with 0.5 oz American cheese (130)
2 oz raisins (130)	banana (110)	cream cheese (100)	1 link turkey sausage (70)	1 cup strawberries (60)		1/2 cup honeydew (30)
light strawberry yogurt (80)		blueberry yogurt (120)	1/2 cup pineapple (70)			2 slices wheat toast (140) with 2 pats butter (70)
**505**	**500**	**610**	**675**	**400**	**530**	**520**
**Lunch**						
8oz apple juice (120)	8oz fruit punch (120)	8oz ginger ale (80)	8oz orange juice (120)	8oz grape juice (170)	8oz lemonade (120)	8oz cranberry juice (120)
Grilled Tilapia with Butter and Herbs, 3.5 oz (130)	8 oz spiral pasta (420) with 1/2 cup marinara sauce (50)	cheeseburger, 1 oz cheese (450) offered with ketchup, lettuce, tomato, pickle	Macaroni and Cheese, 8 oz (460)	grilled cheese (500)	tuna salad sandwich on wheat (340)	turkey (3 oz) sandwich on wheat with mayo (360)
1/2 cup pineapple (70)	2 meatball (200)	1/2 cup raw carrots (35)	1/2 cup green beans (35)	garden salad (15) with ranch dressing packet (140)	garden side salad (15)	whole apple, sliced (80)
potato stars, 3 oz (270)				fresh fruit cup (50)	Italian dressing (110)	1/2 cup broccoli (20)
side caesar salad with ceasar dressing (305)						
**895**	**790**	**565**	**615**	**875**	**585**	**580**
**Dinner**						
8oz whole milk (150)	8oz whole milk (150)	8oz whole milk (150)	8oz whole milk (150)	8 oz Wawa iced tea (90)	8oz whole milk (150)	8oz 2% milk (130)

Meals = 500-600 calories (2.5 cans Boost)
Snacks = 300-400 calories (1.5 cans Boost)

2000 kcal Malnutrition Pathway Meal Plan

grilled chicken breast, sliced (140)	3 oz roast turkey with 3 oz gravy (225)	chicken alfredo with 3 oz chicken, 3 oz pasta, 2 oz sauce (320)	beef fajitas (255) with 0.5 oz shredded cheddar cheese (60)	pepperoni pizza (560)	Chicken LoMein with Veggies (370)	3 chicken tenders (300) offered with 1 packet ketchup
1/2 cup cantaloupe (40)	3/4 cup mashed potatoes (195)	1/2 cup broccoli (20)	1/2 cup cooked carrots (35)	1 cup grapes (60)	Brownie cupcake (185), vanilla ice cream cup (140), 2T Hershey's chocolate syrup (100)	1/2 cup french fries (190) offered with 1 packet ketchup
1/2 cup raw carrot sticks (35)	1/2 cup broccoli with pat of butter (55)	6 oz Cookies & Cream Hershey© ice cream cup (250)	1/2 cup honeydew (30)	1/2 cup broccoli (20)		1/2 cup green beans (35)
Baked Potato (160) with 2 pats butter (70)	chocolate chip cookie (125)	orange (60)	1 brownie cupcake (185)			ice cream sandwich (300)
**595**	**750**	**800**	**715**	**730**	**945**	**955**
**Total Calories: 1995**	**2040**	**1975**	**2005**	**2005**	**2060**	**2055**
**Total Fluid: 720 mL**	**720**	**720**	**720**	**720**	**720**	**720**

Meals = 500-600 calories (2.5 cans Boost)
Snacks = 300-400 calories (1.5 cans Boost)

2400 kcal Malnutrition Pathway Meal Plan

MONDAY	TUESDAY	WEDNESDAY	THURSDAY	FRIDAY	SATURDAY	SUNDAY
**Breakfast**						
8oz whole milk (150)	8oz whole milk (150)	8oz whole milk (150)	8oz whole milk (150)	8oz 2% milk (130)	8oz whole milk (150)	8oz whole milk (150)
1 C oatmeal (145) with 2 oz raisins (130)	breakfast sandwich (1 oz egg on english muffin with 1 oz American cheese) (240)	bagel (240) with cream cheese (100)	4 French toast sticks (260) with syrup (110) and pat butter (35)	3 oz scrambled eggs (135) with 1 oz cheese (75)	2 pancakes (200) with syrup (110) and 2 pats butter (70)	2oz egg omelet with 0.5 oz American cheese (130)
light strawberry yogurt (80)	banana (110)	blueberry yogurt (150)	1 link turkey sausage (70)	1 cup strawberries (60)	turkey bacon, 2 oz (70)	1/2 cup honeydew (30)
			1/2 cup pineapple (70)	wheat toast (70) with 1 pat butter (35)		2 slices wheat toast (140) with 2 pats butter (70)
**505**	**500**	**640**	**695**	**505**	**600**	**520**
**Lunch**						
8oz apple juice (120)	8oz fruit punch (120)	8oz ginger ale (80)	8oz orange juice (120)	8oz grape juice (170)	8oz lemonade (120)	8oz cranberry juice (120)
Grilled Tilapia with Butter and Herbs, 3.5 oz (130)	8 oz spiral pasta (420) with 1/2 cup marinara sauce (50)	cheeseburger, 1 oz cheese (450) offered with ketchup, lettuce, tomato, pickle	Macaroni and Cheese, 8 oz (460)	grilled cheese (500)	tuna salad sandwich on wheat (340)	turkey (3 oz) sandwich on wheat with mayo (360)
1/2 cup pineapple (70)	2 meatball (200)	1/2 cup raw carrots (35)	1/2 cup green beans (35)	garden salad (15) with ranch dressing packet (140)	garden side salad (15)	whole apple, sliced (80)
potato stars, 3 oz (270)	1/2 cup green beans (35)	Sun Chips, 1.5 oz (210)		fresh fruit cup (50)	Italian dressing (110)	1/2 cup broccoli (20)
side caesar salad with ceasar dressing (305)				tomato soup (100)		
**895**	**825**	**775**	**615**	**975**	**585**	**580**
**Snack**						
8oz apple juice (120)	8oz orange juice (120)	8oz grape juice (170)	8oz cranberry juice (120)	8 oz whole milk (150)	8 oz apple juice (120)	8oz fruit punch (120)
1 cup strawberries (60)	hard pretzels (110)	1/2 cup pears (50)	4 peanut butter crackers (130)	apple, sliced (80)	2 oz baked tortilla chips (180)	2 peanut butter crackers (65)
					3.8 oz salsa cup (30)	

Meals = 550-650 calories (2.5 cans Boost)
Snacks = 300-400 calories (1.5 cans Boost)

2400 kcal Malnutrition Pathway Meal Plan

180	230	220	250	230	330	185
**Dinner**						
8 oz whole milk (150)	8 oz whole milk (150)	8 oz whole milk (150)	8 oz whole milk (150)	8 oz Wawa Iced tea (90)	8 oz whole milk (150)	8 oz 2% milk (130)
grilled chicken breast, sliced (140)	3 oz roast turkey with 3 oz gravy (225)	chicken alfredo with 3 oz chicken, 3 oz pasta, 2 oz sauce (320)	beef fajitas (255) with 0.5 oz shredded cheddar cheese (60)	pepperoni pizza (560)	Chicken LoMein with Veggies (370)	4 chicken tenders (400) offered with 2 packets ketchup
1/2 cup cantaloupe (40)	1 cup mashed potatoes (260)	1/2 cup broccoli (20)	1/2 cup cooked carrots (35)	1 cup grapes (60)	Brownie cupcake (185), vanilla ice cream cup (140), 2T Hershey's chocolate syrup (100)	3/4 cup french fries (285) offered with 1 packet ketchup
1/2 cup raw carrot sticks (35)	1/2 cup broccoli with pat of butter (55)	6 oz Cookies & Cream Hershey (c) ice cream cup (250)	1/2 cup honeydew (30)	1/2 cup broccoli (20)		1/2 cup green beans (35) with 1 pat butter (35)
Baked Potato (160) with 2 pats butter (70)	chocolate chip cookie (125)	orange (60)	Milkshake with 4 oz chocolate ice cream, 4 oz whole milk, 3T chocolate syrup (365)			Ice cream sandwich (300)
Sweet Street © Caramel Apple bar (280)						
**875**	**815**	**800**	**895**	**730**	**945**	**1185**
**Total Calories: 2455**	**2370**	**2435**	**2455**	**2440**	**2460**	**2470**
**Total Fluid: 960 mL**	**960**	**960**	**960**	**960**	**960**	**960**

Meals = 550-650 calories (2.5 cans Boost)
Snacks = 300-400 calories (1.5 cans Boost)

2800 kcal Malnutrition Pathway Meal Plan

MONDAY	TUESDAY	WEDNESDAY	THURSDAY	FRIDAY	SATURDAY	SUNDAY
**Breakfast**						
8oz whole milk (150)	8oz whole milk (150)	8oz whole milk (150)	8oz whole milk (150)	8oz whole milk (150)	8oz whole milk (150)	8oz whole milk (150)
1 C oatmeal (145) with 2 oz raisins (130)	breakfast sandwich (1 oz egg on biscuit with 2 oz American cheese) (415)	bagel (240)	5 French toast sticks (325) with syrup (110) and pat butter (35)	3 oz scrambled eggs (135) with 1 oz cheese (75)	2 pancakes (200) with syrup (110) and 2 pats butter (70)	2oz egg omelet with 1 oz American cheese (165)
light strawberry yogurt (80)	banana (110)	cream cheese (100)	2 links turkey sausage (140)	1 cup strawberries (60)	turkey bacon, 3 oz (105)	1/2 cup honeydew (30)
hard boiled egg (75)		blueberry yogurt (150)	1/2 cup pineapple (70)	biscuit (220)	1 cup watermelon (50)	2 slices wheat toast (140) with 2 pats butter (70)
**580**	**675**	**640**	**830**	**640**	**685**	**555**
**Lunch**						
8oz apple juice (120)	8oz fruit punch (120)	8oz ginger ale (80)	8oz orange juice (120)	8oz grape juice (170)	8oz lemonade (120)	8oz cranberry juice (120)
Grilled Tilapia with Butter and Herbs, 3.5 oz (130)	8 oz spiral pasta (420) with 1/2 cup marinara sauce (50)	cheeseburger, 1 oz cheese (450) offered with ketchup, lettuce, tomato, pickle	Macaroni and Cheese, 8 oz (460)	grilled cheese (500)	tuna salad sandwich on wheat with 1 oz Swiss cheese (450)	turkey (3 oz) sandwich on wheat with mayo and 1 oz Swiss cheese (470)
1/2 cup pineapple (70)	2 meatballs (200)	1/2 cup raw carrots (35)	1/2 cup green beans with 1 pat butter (70)	garden salad (15) with ranch dressing packet (140)	garden side salad (15)	whole apple, sliced (80)
potato stars, 3 oz (270)	1/2 cup green beans (35) with 1 pat butter (35)	Sun Chips, 1.5 oz (210)	apple (80)	fresh fruit cup (50)	Italian dressing (110)	1/2 cup broccoli (20)
side caesar salad with ceasar dressing (305)				tomato soup (100)	1/2 cup pineapple (70)	
**895**	**860**	**775**	**730**	**975**	**765**	**690**

Meals = 600-700 calories (3 cans Boost)
Snacks = 350-450 calories (1.5 cans Boost)

2800 kcal Malnutrition Pathway Meal Plan

**Snack**						
8oz apple juice (120)	8oz orange juice (120)	8oz grape juice (170)	8oz cranberry juice (120)	8 oz whole milk (150)	8 oz apple juice (120)	8oz fruit punch (120)
1 cup strawberries (60)	soft pretzel (180)	1/2 cup pears (50)	2 oz hummus (150)	apple, sliced (80)	2 oz baked tortilla chips (180)	1/2 cup pineapple (70)
vanilla pudding (130)	1 oz cheese sauce (70)	half peanut butter and jelly sandwich on wheat (160)	Ritz crackers, 7 (110)		3.8 oz salsa cup (30)	Sweet Street © Pecan Chocolate Chunk Bar (300)
					1 oz cheese sauce (70)	
**310**	**370**	**380**	**380**	**230**	**400**	**490**
**Dinner**						
8oz whole milk (150)	8oz whole milk (150)	8oz whole milk (150)	8oz whole milk (150)	8 oz Wawa iced tea (90)	8oz whole milk (150)	8oz 2% milk (130)
1.5 grilled chicken breasts, sliced (210)	3 oz roast turkey with 3 oz gravy (225)	chicken alfredo with 3 oz chicken, 6 oz pasta, 4 oz sauce (560)	beef fajitas (255) with 0.5 oz shredded cheddar cheese (60)	pepperoni pizza (560)	Chicken LoMein with Veggies (370)	3 chicken tenders (300) offered with 1 packet ketchup
1/2 cup cantaloupe (40)	1 cup mashed potatoes (260)	1/2 cup broccoli (20)	1/2 cup cooked carrots (35)	1 cup grapes (60)	Brownie cupcake (185), vanilla ice cream cup (140), 2T chocolate syrup (100)	3/4 cup french fries (285) offered with 1 packet ketchup
1/2 cup raw carrot sticks (35) with Ranch dressing packet (140)	1/2 cup broccoli with pat of butter (55)	6 oz Cookies & Cream Hershey © ice cream cup (250)	1/2 cup honeydew (30)	1/2 cup broccoli (20) with 1 pat butter (35)		1/2 cup green beans (35) with 1 pat butter (35)
Baked Potato (160) with 2 pats butter (70)	2 chocolate chip cookies (250)	orange (60)	Milkshake with 4 oz chocolate ice cream, 4 oz whole milk, 3T chocolate syrup (365)	Tastykake© Oatmeal Raisin Cookie Bar (200)		Ice cream sandwich (300)
Sweet Street © Caramel Apple bar (280)						
**1085**	**940**	**1040**	**895**	**965**	**945**	**1085**
**Total Calories: 2870**	**2845**	**2835**	**2835**	**2810**	**2795**	**2820**

Meals = 600-700 calories (3 cans Boost)

Snacks = 350-450 calories (1.5 cans Boost)

2800 kcal Malnutrition Pathway Meal Plan

**Total Fluid: 960 mL**	**960**	**960**	**960**	**960**	**960**	**960**

Meals = 600-700 calories (3 cans Boost)
Snacks = 350-450 calories (1.5 cans Boost)

3000 kcal Malnutrition Pathway Meal Plan

MONDAY	TUESDAY	WEDNESDAY	THURSDAY	FRIDAY	SATURDAY	SUNDAY
**Breakfast**						
8oz whole milk (150)	8oz whole milk (150)	8oz whole milk (150)	8oz whole milk (150)	8oz whole milk (150)	8oz whole milk (150)	8oz whole milk (150)
1 C oatmeal (145)	breakfast sandwich (1 oz egg on biscuit with 2 oz American cheese) (415)	bagel (240)	5 French toast sticks (325) with syrup (110) and pat butter (35)	3 oz scrambled eggs (135) with 1 oz cheese (75)	2 pancakes (200) with syrup (110) and 2 pats butter (70)	2oz egg omelet with 1 oz American cheese (165)
2 oz raisins (130)	banana (110)	cream cheese (100)	2 links turkey sausage (140)	1 cup strawberries (60)	turkey bacon, 3 oz (105)	1/2 cup honeydew (30)
light strawberry yogurt (80)		blueberry yogurt (150)	1/2 cup pineapple (70)	biscuit (220)	1 cup watermelon (50)	2 slices wheat toast (140) with 2 pats butter (70)
hard boiled egg (75)						
**580**	**675**	**640**	**830**	**640**	**685**	**555**
**Morning Snack**						
8oz whole milk (150)	8oz whole milk (150)	8oz whole milk (150)	8oz whole milk (150)	8oz whole milk (150)	8oz whole milk (150)	8oz whole milk (150)
1/2 cup peaches (50)	light strawberry yogurt (80)	fruit loops (80)	1 cup grapes (60)	nature valley granola bar (90)	strawberry yogurt (120)	Cheerios (75)
**200**	**230**	**230**	**210**	**240**	**270**	**225**
**Lunch**						
8oz apple juice (120)	8oz fruit punch (120)	8oz ginger ale (80)	8oz orange juice (120)	8oz grape juice (170)	8oz lemonade (120)	8oz cranberry juice (120)
Grilled Tilapia with Butter and Herbs, 3.5 oz (130)	8 oz spiral pasta (420) with 1/2 cup marinara sauce (50)	cheeseburger, 1 oz cheese (450) offered with ketchup, lettuce, tomato, pickle	Macaroni and Cheese, 8 oz (460)	grilled cheese (500)	tuna salad sandwich on wheat with 1 oz Swiss cheese (450)	turkey (3 oz) sandwich on wheat with mayo and 1 oz Swiss cheese (470)
1/2 cup pineapple (70)	2 meatball (200)	1/2 cup raw carrots (35)	1/2 cup green beans with 1 pat butter (70)	garden salad (15) with ranch dressing packet (140)	garden side salad (15)	whole apple, sliced (80)
potato stars, 3 oz (270)	1/2 cup green beans (35) with 1 pat butter (35)	Sun Chips, 1.5 oz (210)	apple (80)	fresh fruit cup (50)	Italian dressing (110)	1/2 cup broccoli (20)

Meals = 650-750 calories (3 cans Boost)
Snacks = 400-500 calories (2 cans Boost)

3000 kcal Malnutrition Pathway Meal Plan

side caesar salad with ceasar dressing (305)				tomato soup (100)	1/2 cup pineapple (70)	
**895**	**860**	**775**	**730**	**975**	**765**	**690**
**Afternoon Snack**						
8oz apple juice (120)	8oz orange juice (120)	8oz grape juice (170)	8oz cranberry juice (120)	8 oz whole milk (150)	8 oz apple juice (120)	8oz fruit punch (120)
1 cup strawberries (60)	soft pretzel (180)	1/2 cup pears (50)	2 oz hummus (150)	apple, sliced (80)	2 oz baked tortilla chips (180)	1/2 cup pineapple (70)
vanilla pudding (130)	1 oz cheese sauce (70)	half peanut butter and jelly sandwich on wheat (160)	Ritz crackers, 7 (110)		3.8 oz salsa cup (30)	Sweet Street © Pecan Chocolate Chunk Bar (300)
					1 oz cheese sauce (70)	
**310**	**370**	**380**	**380**	**230**	**400**	**490**
**Dinner**						
8oz whole milk (150)	8oz whole milk (150)	8oz whole milk (150)	8oz whole milk (150)	8 oz Wawa iced tea (90)	8oz whole milk (150)	8oz 2% milk (130)
1.5 grilled chicken breasts, sliced (210)	3 oz roast turkey with 3 oz gravy (225)	chicken alfredo with 3 oz chicken, 6 oz pasta, 4 oz sauce (560)	beef fajitas (255)	pepperoni pizza (560)	Chicken LoMein with Veggies (370)	3 chicken tenders (300) offered with 1 packet ketchup
1/2 cup cantaloupe (40)	1 cup mashed potatoes (260)	1/2 cup broccoli (20)	1/2 cup cooked carrots (35)	1 cup grapes (60)	Brownie cupcake (185), vanilla ice cream cup (140), 2T chocolate syrup (100)	3/4 cup french fries (285) offered with 1 packet ketchup
1/2 cup raw carrot sticks (35) with ranch dressing packet (140)	1/2 cup broccoli with pat of butter (55)	6 oz Cookies & Cream Hershey © ice cream cup (250)	1/2 cup honeydew (30)	1/2 cup broccoli with 1 pat butter (55)		1/2 cup green beans (35) with pat butter (35)
Baked Potato (160) with 2 pats butter (70)	2 chocolate chip cookies (250)	orange (60)	0.5 oz shredded cheddar cheese (60)	Tastykake © Oatmeal Raisin Cookie Bar (200)		Ice cream sandwich (300)

Meals = 650-750 calories (3 cans Boost)
Snacks = 400-500 calories (2 cans Boost)

3000 kcal Malnutrition Pathway Meal Plan

Sweet Street © Caramel Apple bar (280)			Milkshake with 4 oz chocolate ice cream, 4 oz whole milk, 3T chocolate syrup (365)			
**1085**	**940**	**1040**	**895**	**965**	**945**	**1085**
**3070**	**3075**	**3065**	**3045**	**3050**	**3065**	**3045**
**1200**	**1200**	**1200**	**1200**	**1200**	**1200**	**1200**

Meals = 650-750 calories (3 cans Boost)
Snacks = 400-500 calories (2 cans Boost)

3200 kcal Malnutrition Pathway Meal Plan

MONDAY	TUESDAY	WEDNESDAY	THURSDAY	FRIDAY	SATURDAY	SUNDAY
**Breakfast**						
8oz whole milk (150)	8oz whole milk (150)	8oz whole milk (150)	8oz whole milk (150)	8oz whole milk (150)	8oz whole milk (150)	8oz whole milk (150)
1 C oatmeal (145)	breakfast sandwich (1 oz egg on biscuit with 2 oz American cheese) (415)	bagel (240)	5 French toast sticks (325) with syrup (110) and pat butter (35)	3 oz scrambled eggs (135) with 1 oz cheese (75)	2 pancakes (200) with syrup (110) and 2 pats butter (70)	4oz egg omelet with 1 oz American cheese (255)
2 oz raisins (130)	banana (110)	cream cheese (100)	2 links turkey sausage (140)	1 cup strawberries (60)	turkey bacon, 3 oz (105)	1/2 cup honeydew (30)
light strawberry yogurt (80)		blueberry yogurt (150)	1/2 cup pineapple (70)	biscuit (220)	1 cup watermelon (50)	2 slices wheat toast (140) with 2 pats butter (70)
hard boiled egg (75)						
**580**	**675**	**640**	**830**	**640**	**685**	**645**
**Morning Snack**						
8oz whole milk (150)	8oz whole milk (150)	8oz whole milk (150)	8oz whole milk (150)	8oz whole milk (150)	8oz whole milk (150)	8oz whole milk (150)
1/2 cup peaches (50)	Lemon yogurt, regular (150)	fruit loops (80)	1 cup grapes (60)	nature valley granola bar (90)	strawberry yogurt (120)	Cheerios (75)
corn muffin (220)	2 oz granola (120)		2 oz cubed cheddar cheese (220)	banana (110)	1/2 cinnamon raisin bagel (125) with pat of butter (35)	
**420**	**420**	**230**	**430**	**350**	**430**	**225**
**Lunch**						
8oz apple juice (120)	8oz fruit punch (120)	8oz ginger ale (80)	8oz orange juice (120)	8oz grape juice (170)	8oz lemonade (120)	8oz cranberry juice (120)
Grilled Tilapia with Butter and Herbs, 3.5 oz (130)	8 oz spiral pasta (420) with 1/2 cup marinara sauce (50)	cheeseburger, 1 oz cheese (450) offered with ketchup, lettuce, tomato, pickle	Macaroni and Cheese, 8 oz (460)	grilled cheese (500)	tuna salad sandwich on wheat with 1 oz Swiss cheese (450)	turkey (3 oz) sandwich on wheat with mayo and 1 oz Swiss cheese (470)
1/2 cup pineapple (70)	2 meatballs (200)	1/2 cup raw carrots (35)	1/2 cup green beans with 1 pat butter (70)	garden salad (15) with ranch dressing packet (140)	garden side salad (15)	whole apple, sliced (80)

Meals = 700-800 calories (3 cans Boost)
Snacks = 400-500 calories (2 cans Boost)

3200 kcal Malnutrition Pathway Meal Plan

potato stars, 3 oz (270)	1/2 cup green beans (35) with 1 pat butter (35)	Sun Chips, 1.5 oz (210)	apple (80)	fresh fruit cup (50)	Italian dressing (110)	1/2 cup broccoli (20)
side caesar salad with ceasar dressing (305)				tomato soup (100)	1/2 cup pineapple (70)	
**895**	**860**	**775**	**730**	**975**	**765**	**690**
**Afternoon Snack**						
8oz apple juice (120)	8oz orange juice (120)	8oz grape juice (170)	8oz cranberry juice (120)	8 oz whole milk (150)	8 oz apple juice (120)	8oz fruit punch (120)
1 cup strawberries (60)	soft pretzel (180)	1/2 cup pears (50)	2 oz hummus (150)	apple, sliced (80)	2 oz baked tortilla chips (180)	1/2 cup pineapple (70)
vanilla pudding (130)	1 oz cheese sauce (70)	peanut butter and jelly sandiwch on wheat (320)	Ritz crackers, 7 (110)	4 Nilla wafers (70)	3.8 oz salsa cup (30)	Sweet Street © Pecan Chocolate Chunk Bar (300)
					1 oz cheese sauce (70)	
**310**	**370**	**540**	**380**	**300**	**400**	**490**
**Dinner**						
8oz whole milk (150)	8oz whole milk (150)	8oz whole milk (150)	8oz whole milk (150)	8 oz Wawa iced tea (90)	8oz whole milk (150)	8oz whole milk (150)
1.5 grilled chicken breasts, sliced (210)	3 oz roast turkey with 3 oz gravy (225)	chicken alfredo with 3 oz chicken, 6 oz pasta, 4 oz sauce (560)	beef fajitas (255)	pepperoni pizza (560)	Chicken LoMein with Veggies (370)	4 chicken tenders (400) offered with 2 packets ketchup
1/2 cup cantaloupe (40)	1 cup mashed potatoes (260)	1/2 cup broccoli (20)	1/2 cup cooked carrots (35)	1 cup grapes (60)	Brownie cupcake (185), vanilla ice cream cup (140), 2T chocolate syrup (200)	3/4 cup french fries (285) offered with 1 packet ketchup
1/2 cup raw carrot sticks (35) with Ranch dressing packet (140)	1/2 cup broccoli with pat of butter (55)	6 oz Cookies & Cream Hershey © ice cream cup (250)	1/2 cup honeydew (30)	1/2 cup broccoli (20) with pat butter (35)		1/2 cup green beans (35)
Baked Potato (160) with 2 pats butter (70)	2 chocolate chip cookies (250)	orange (60)	0.5 oz shredded cheddar cheese (60)	Tastykake © Oatmeal Raisin Cookie Bar (200)		Ice cream sandwich (300)

Meals = 700-800 calories (3 cans Boost)
Snacks = 400-500 calories (2 cans Boost)

3200 kcal Malnutrition Pathway Meal Plan

Sweet Street © Caramel Apple bar (280)			Milkshake with 4 oz chocolate ice cream, 4 oz whole milk, 3T chocolate syrup (365)			
**1085**	**940**	**1040**	**895**	**965**	**945**	**1170**
**3290**	**3265**	**3225**	**3265**	**3230**	**3225**	**3220**
**1200**	**1200**	**1200**	**1200**	**1200**	**1200**	**1200**

Meals = 700-800 calories (3 cans Boost)
Snacks = 400-500 calories (2 cans Boost)

3400 kcal Malnutrition Pathway Meal Plan

MONDAY	TUESDAY	WEDNESDAY	THURSDAY	FRIDAY	SATURDAY	SUNDAY
**Breakfast**						
8oz whole milk (150)	8oz whole milk (150)	8oz whole milk (150)	8oz whole milk (150)	8oz whole milk (150)	8oz whole milk (150)	8oz whole milk (150)
1 C oatmeal (145) with 2 oz raisins (130)	breakfast sandwich (2 oz egg on biscuit with 2.5 oz American cheese (495)	bagel (240)	5 French toast sticks (325) with syrup (110) and pat butter (35)	3 oz scrambled eggs (135) with 1 oz cheese (75)	3 pancakes (300) with 2 syrups (220) and 2 pats butter (70)	4oz egg omelet with 2 oz American cheese (330)
light strawberry yogurt (80)	banana (110)	cream cheese (100)	sausage patty (180)	1 cup strawberries (60)	turkey bacon, 3 oz (105)	1/2 cup honeydew (30)
hard boiled egg (75)		blueberry yogurt (150)	1/2 cup pineapple (70)	biscuit (220)	1 cup watermelon (50)	2 slices wheat toast (140) with 2 pats butter (70)
**580**	**755**	**690**	**870**	**640**	**895**	**720**
**Morning Snack**						
8oz whole milk (150)	8oz whole milk (150)	8oz whole milk (150)	8oz whole milk (150)	8oz whole milk (150)	8oz whole milk (150)	8oz whole milk (150)
1/2 cup peaches (50)	Lemon yogurt, regular (150)	fruit loops (80)	1 cup grapes (60)	nature valley granola bar (90)	strawberry yogurt (120)	Cheerios (75)
corn muffin (220) with pat butter (35)	2 oz granola (120)	1 cup grapes (60)	2 oz cubed cheddar cheese (220)	banana (110)	1/2 cinnamon raisin bagel (125) with pat of butter (35)	banana (110)
**455**	**420**	**290**	**430**	**350**	**430**	**335**
**Lunch**						
8oz apple juice (120)	8oz fruit punch (120)	8oz ginger ale (80)	8oz orange juice (120)	8oz grape juice (170)	8oz lemonade (120)	8oz cranberry juice (120)
Grilled Tilapia with Butter and Herbs, 3.5 oz (130)	10 oz spiral pasta (525) with 3/4 cup marinara sauce (75)	cheeseburger, 2 oz cheese (525) offered with ketchup, lettuce, tomato, pickle	Macaroni and Cheese, 8 oz (460)	grilled cheese (500)	tuna salad sandwich on wheat with 1 oz Swiss cheese (450)	turkey (3 oz) sandwich on wheat with mayo and 2 oz Swiss cheese (580)
1/2 cup pineapple (70)	2 meatballs (200)	1/2 cup raw carrots (35)	1/2 cup green beans with 1 pat butter (70)	garden salad (15) with ranch dressing packet (140)	garden side salad (15)	whole apple, sliced (80)
potato stars, 3 oz (270)	1/2 cup green beans (35) with 1 pat butter (35)	Sun Chips, 1.5 oz (210)	apple (80)	fresh fruit cup (50)	Italian dressing (110)	1/2 cup broccoli (20) with pat butter (35)

Meals = 750-850 calories (3.5 cans Boost)

Snacks = 450-550 calories (2 cans Boost)

3400 kcal Malnutrition Pathway Meal Plan

side caesar salad with ceasar dressing (305)				tomato soup (100)	1/2 cup pineapple (70)	
**895**	**990**	**850**	**730**	**975**	**765**	**835**
**Afternoon Snack**						
8oz apple juice (120)	8oz orange juice (120)	8oz grape juice (170)	8oz cranberry juice (120)	8 oz whole milk (150)	8 oz apple juice (120)	8oz fruit punch (120)
1 cup strawberries (60)	soft pretzel (180)	1/2 cup pears (50)	2 oz hummus (150)	apple, sliced (80)	2 oz baked tortilla chips (180)	1/2 cup pineapple (70)
vanilla pudding (130)	1 oz cheese sauce (70)	peanut butter and jelly sandiwch on wheat (320)	Ritz crackers, 7 (110)	6 Nilla wafers (105)	3.8 oz salsa cup (30)	Sweet Street © Pecan Chocolate Chunk Bar (300)
			1/2 cup sliced cucumbers (25)		1 oz cheese sauce (70)	
**310**	**370**	**540**	**405**	**335**	**400**	**490**
**Dinner**						
8oz whole milk (150)	8oz whole milk (150)	8oz whole milk (150)	8oz whole milk (150)	8 oz Wawa iced tea (90)	8oz whole milk (150)	8oz whole milk (150)
1.5 grilled chicken breasts, sliced (210)	3 oz roast turkey with 3 oz gravy (225)	chicken alfredo with 3 oz chicken, 6 oz pasta, 4 oz sauce (560)	beef fajitas (255) with sour cream (50) and 0.5 oz shredded cheddar (60)	pepperoni pizza (560)	Chicken LoMein with veggies, add extra half chicken breast (420)	3 chicken tenders (300) offered with 1 packet ketchup
1/2 cup cantaloupe (40)	1 cup mashed potatoes (260)	1/2 cup broccoli (20)	1/2 cup cooked carrots (35)	1 cup grapes (60)	Brownie cupcake (185), vanilla ice cream cup (140), 2T chocolate syrup (100)	3/4 cup french fries (285) offered with 1 packet ketchup
1/2 cup raw carrot sticks (35)	1/2 cup broccoli with pat of butter (55)	6 oz Cookies & Cream Hershey ice cream cup (250)	1/2 cup honeydew (30)	1/2 cup broccoli w/1 pat butter (55)		1/2 cup green beans (35)
Baked Potato (160) with 2 pats butter (70)	2 chocolate chip cookies (250)	orange (60)	Milkshake with 4 oz chocolate ice cream, 4 oz whole milk, 3T chocolate syrup (365)	Tastykake © Butter Kreme Snack (3 cupcakes) (400)		Ice cream sandwich (300)

Meals = 750-850 calories (3.5 cans Boost)
Snacks = 450-550 calories (2 cans Boost)

3400 kcal Malnutrition Pathway Meal Plan

2 Street Sweet © Caramel Apple bars (560)						
**1225**	**940**	**1040**	**945**	**1165**	**995**	**1070**
**Total calories: 3465**	**3475**	**3410**	**3380**	**3465**	**3485**	**3450**
**Total Fluid: 1200 mL**	**1200**	**1200**	**1200**	**1200**	**1200**	**1200**

Meals = 750-850 calories (3.5 cans Boost)
Snacks = 450-550 calories (2 cans Boost)

3600 kcal Malnutrition Pathway Meal Plan

MONDAY	TUESDAY	WEDNESDAY	THURSDAY	FRIDAY	SATURDAY	SUNDAY
**Breakfast**						
12oz whole milk (225)	12oz whole milk (225)	12oz whole milk (225)	12oz whole milk (225)	12oz whole milk (225)	12oz whole milk (225)	12oz whole milk (225)
1 C oatmeal (145) with 2 oz raisins (130)	breakfast sandwich (2 oz egg on biscuit with 2.5 oz American cheese (495)	bagel (240) with 2 cream cheese packets (200)	5 French toast sticks (325) with syrup (110) and pat butter (35)	3 oz scrambled eggs (135) with 2 oz cheese (150)	3 pancakes (300) with 2 syrups (220) and 3 pats butter (105)	4oz egg omelet with 2 oz American cheese (330)
peach yogurt, regular (150)	banana (110)	blueberry yogurt (150)	sausage patty (180)	1 cup strawberries (60)	turkey bacon, 3 oz (105)	1/2 cup honeydew (30)
hard boiled egg (75)		watermelon (50)	1/2 cup pineapple (70)	biscuit (220) with 1 pat butter (35)	1 cup watermelon (50)	2 slices wheat toast (140) with 2 pats butter (70)
**725**	**830**	**865**	**945**	**825**	**1005**	**795**
**Snack**						
8 oz whole milk (150)	8 oz whole milk (150)	8 oz whole milk (150)	8 oz whole milk (150)	8 oz whole milk (150)	8 oz whole milk (150)	8 oz whole milk (150)
1/2 cup peaches (50)	Lemon yogurt, regular (150)	fruit loops (80)	1 cup grapes (60)	Nature Valley granola bar (90)	strawberry yogurt (120) with 1 cup strawberries (60)	Cheerios (75)
corn muffin (220) with pat butter (35)	2 oz granola (120)	1 cup grapes (60)	2 oz cubed cheddar cheese (220)	banana (110)	1/2 cinnamon raisin bagel (125)	banana (110)
**455**	**420**	**290**	**430**	**350**	**455**	**335**
**Lunch**						
8oz apple juice (120)	8oz fruit punch (120)	8oz ginger ale (80)	8oz orange juice (120)	8oz grape juice (170)	8oz lemonade (120)	8oz cranberry juice (120)
Grilled Tilapia with Butter and Herbs, 3.5 oz (130)	10 oz spiral pasta (525) with 3/4 cup marinara sauce (75)	cheeseburger, 2 oz cheese (525) offered with ketchup, lettuce, tomato, pickle	Macaroni and Cheese, 8 oz (460)	grilled cheese (500)	tuna salad sandwich on wheat with 1 oz Swiss cheese (450)	turkey (3 oz) sandwich on wheat with mayo and 2 oz Swiss cheese (580)
1/2 cup pineapple (70)	2 meatball (200)	1/2 cup raw carrots (35)	1/2 cup green beans with 1 pat butter (70)	garden salad (15) with ranch dressing packet (140)	garden side salad (15)	whole apple, sliced (80)
potato stars, 3 oz (270)	1/2 cup green beans (35) with 1 pat butter (35)	Sun Chips, 1.5 oz (210)	apple (80)	fresh fruit cup (50)	Italian dressing (110)	1/2 cup broccoli (20) with pat butter (35)

Meals = 800-900 calories (3.5 cans Boost)
Snacks = 450-550 calories (2 cans Boost)

3600 kcal Malnutrition Pathway Meal Plan

side caesar salad with ceasar dressing (305)	1/2 cup peaches (50)	ranch dressing packet (140)		tomato soup (100)	1/2 cup pineapple (70)	hard pretzels (110)
**895**	**1040**	**990**	**730**	**975**	**765**	**945**
**Snack**						
8oz apple juice (120)	8oz orange juice (120)	8oz grape juice (170)	8oz cranberry juice (120)	8 oz whole milk (150)	8 oz apple juice (120)	8oz fruit punch (120)
1 cup strawberries (60)	soft pretzel (180)	1/2 cup pears (50)	3 oz hummus (225)	apple, sliced (80)	2 oz baked tortilla chips (180)	1/2 cup pineapple (70)
vanilla pudding (130)	1 oz cheese sauce (70)	peanut butter and jelly sandwich on wheat (320)	Ritz crackers, 10 (160)	6 Nilla wafers (105)	3.8 oz salsa cup (30)	Sweet Street © Pecan Chocolate Chunk Bar (300)
			1/2 cup sliced cucumbers (25)		1 oz cheese sauce (70)	
**310**	**370**	**540**	**530**	**335**	**400**	**490**
**Dinner**						
8oz whole milk (150)	8oz whole milk (150)	8oz whole milk (150)	8oz whole milk (150)	8 oz Wawa iced tea (90)	8oz whole milk (150)	8oz whole milk (150)
1.5 grilled chicken breasts, sliced (210)	4 oz roast turkey with 3 oz gravy (290)	chicken alfredo with 3 oz chicken, 6 oz pasta, 4 oz sauce (560)	beef fajitas (255) with sour cream packet (50) and 0.5 oz shredded cheddar (60)	pepperoni pizza (560)	Chicken LoMein with veggies, add extra half chicken breast (420)	3 chicken tenders (300) offered with 1 packet ketchup
1/2 cup cantaloupe (40)	1 cup mashed potatoes (260)	1/2 cup broccoli (20)	1/2 cup cooked carrots (35)	1 cup grapes (60)	Brownie cupcake (185), vanilla ice cream cup (140), 2T chocolate syrup (100)	3/4 cup french fries (285) offered with 1 packet ketchup
1/2 cup raw carrot sticks (35)	1/2 cup broccoli with pat of butter (55)	6 oz Cookies & Cream Hershey © ice cream cup (250)	1/2 cup honeydew (30)	1/2 cup broccoli w/1 pat butter (55)		1/2 cup green beans (35)
Baked Potato (160) with 2 pats butter (70)	2 chocolate chip cookies (250)	orange (60)	Milkshake with 4oz chocolate ice cream, 4oz whole milk, 3T chocolate syrup (365)	Tastykake© Butter Kreme Snack (3 cupcakes) (400)		Ice cream sandwich (300)
2 Street Sweet © Caramel Apple bars (560)						
**1225**	**1005**	**1040**	**945**	**1165**	**995**	**1070**

Meals = 800-900 calories (3.5 cans Boost)

Snacks = 450-550 calories (2 cans Boost)

3600 kcal Malnutrition Pathway Meal Plan

**Total Calories: 3610**	**3665**	**3725**	**3580**	**3650**	**3620**	**3635**
**Total Fluid: 1320 mL**	**1320**	**1320**	**1320**	**1320**	**1320**	**1320**

Meals = 800-900 calories (3.5 cans Boost)
Snacks = 450-550 calories (2 cans Boost)

3800 kcal Malnutrition Pathway Meal Plan

MONDAY	TUESDAY	WEDNESDAY	THURSDAY	FRIDAY	SATURDAY	SUNDAY
**Breakfast**						
12oz whole milk (225)	12oz whole milk (225)	12oz whole milk (225)	12oz whole milk (225)	12oz whole milk (225)	12oz whole milk (225)	12oz whole milk (225)
1 C oatmeal (145) with 2 oz raisins (130)	breakfast sandwich (2 oz egg on biscuit with 2.5 oz American cheese (495)	bagel (240) with 2 cream cheese packets (200)	5 French toast sticks (325) with syrup (110) and pat butter (35)	3 oz scrambled eggs (135) with 2 oz cheese (150)	3 pancakes (300) with 2 syrups (220) and 3 pats butter (105)	4oz egg omelet with 2 oz American cheese (330)
peach yogurt, regular (150)	banana (110)	blueberry yogurt (150)	sausage patty (180)	1 cup strawberries (60)	turkey bacon, 3 oz (105)	1/2 cup honeydew (30)
hard boiled egg (75)		watermelon (50)	1/2 cup pineapple (70)	biscuit (220) with 1 pat butter (35)	1 cup watermelon (50)	2 slices wheat toast (140) with 2 pats butter (70)
**725**	**830**	**865**	**945**	**825**	**1005**	**795**
**Snack**						
12oz whole milk (225)	12oz whole milk (225)	12oz whole milk (225)	12 oz whole milk (225)	12oz whole milk (225)	12oz whole milk (225)	12oz whole milk (225)
1/2 cup peaches (50)	Lemon yogurt, regular (150)	fruit loops (80)	1 cup grapes (60)	2 nature valley granola bars (180)	strawberry yogurt (120) with 1 cup strawberries (60)	Cheerios, 2 boxes (150)
bagel (240) with peanut butter packet (125)	2 oz granola (120)	1 cup grapes (60)	2 oz cubed cheddar cheese (220)	banana (110)	1/2 cinnamon raisin bagel (125) with pat of butter (35)	banana (110)
**640**	**495**	**365**	**505**	**515**	**565**	**485**
**Lunch**						
8oz apple juice (120)	8oz fruit punch (120)	8oz ginger ale (80)	8oz orange juice	8oz grape juice (170)	8oz lemonade (120)	8oz cranberry juice
Grilled Tilapia with Butter and Herbs, 3.5 oz (130)	10 oz spiral pasta (525) with 3/4 cup marinara sauce (75)	cheeseburger, 2 oz cheese (525) offered with ketchup, lettuce, tomato, pickle	Macaroni and Cheese, 8 oz (460)	grilled cheese (500)	tuna salad sandwich on wheat with 1 oz Swiss cheese (450)	turkey (3 oz) sandwich on wheat with mayo and 2 oz Swiss cheese (580)
1/2 cup pineapple (70)	2 meatballs (200)	1/2 cup raw carrots (35)	1/2 cup green beans with 1 pat butter (70)	garden salad (15) with ranch dressing packet (140)	garden side salad (15)	whole apple, sliced (80)
potato stars, 3 oz (270)	1/2 cup green beans (35) with 1 pat butter (35)	Sun Chips, 1.5 oz (210)	apple (80)	fresh fruit cup (50)	Italian dressing (110)	1/2 cup broccoli (20) with pat butter (35)

Meals = 800-900 calories (3.5 cans Boost)
Snacks = 500-600 calories (2.5 cans Boost)

3800 kcal Malnutrition Pathway Meal Plan

side caesar salad with ceasar dressing (305)	1/2 cup peaches (50)	ranch dressing packet (140)	sugar cookie (125)	tomato soup (100)	1/2 cup pineapple (70)	hard pretzels (110)
	Rice Krispie Treat (150)					
**895**	**1190**	**990**	**855**	**975**	**765**	**945**
**Snack**						
8oz apple juice (120)	8oz orange juice (120)	8oz grape juice (170)	8oz cranberry juice (120)	8 oz whole milk (150)	8 oz apple juice (120)	8oz fruit punch (120)
1 cup strawberries (60)	soft pretzel (180)	1/2 cup pears (50)	3 oz hummus (225)	apple, sliced (80)	3 oz baked tortilla chips (270)	1/2 cup pineapple (70)
vanilla pudding (130)	1 oz cheese sauce (70)	peanut butter and jelly sandwich on wheat (320)	Stacy's © Naked Pita chips, 1.5 oz (200)	6 Nilla wafers (105)	3.8 oz salsa cup (30)	Sweet Street © Pecan Chocolate Chunk Bar (300)
			1/2 cup sliced cucmbers (25)		1 oz cheese sauce (70)	
**310**	**370**	**540**	**570**	**335**	**490**	**490**
**Dinner**						
8oz whole milk (150)	8oz whole milk (150)	8oz whole milk (150)	8oz whole milk (150)	8 oz Wawa iced tea (90)	8oz whole milk (150)	8oz whole milk (150)
2 grilled chicken breasts, sliced (280)	4 oz roast turkey with 3 oz gravy (290)	chicken alfredo with 3 oz chicken, 6 oz pasta, 4 oz sauce (560)	beef fajitas (255) with sour cream packet (50) and 0.5 oz shredded cheddar cheese (60)	pepperoni pizza (560)	Chicken LoMein with veggies, add extra half chicken breast (420)	3 chicken tenders (300) offered with 1 packet ketchup
1/2 cup cantaloupe (40)	1 cup mashed potatoes (260)	1/2 cup broccoli (20)	1/2 cup cooked carrots (35) with 1 pat butter (35)	1 cup grapes (60)	Brownie cupcake (185), vanilla ice cream cup (140), 2T chocolate syrup (100)	3/4 cup french fries (285) offered with 1 packet ketchup
1/2 cup raw carrot sticks (35)	1/2 cup broccoli with pat of butter (55)	6 oz Cookies & Cream Hershey © ice cream cup (250)	1/2 cup honeydew (30)	1/2 cup broccoli w/1 pat butter (55)		1/2 cup green beans (35) with 1 pat butter (35)
Baked Potato (160) with 2 pats butter (70)	2 chocolate chip cookies (250)	orange (60)	Milkshake with 4 oz chocolate ice cream, 4 oz whole milk, 3T chocolate syrup (365)	Tastykake© Butter Kreme Snack (3 cupcakes) (400)		Ice cream sandwich (300)

Meals = 800-900 calories (3.5 cans Boost)
Snacks = 500-600 calories (2.5 cans Boost)

3800 kcal Malnutrition Pathway Meal Plan

2 Sweet Street © Caramel Apple bars (560)						
**1295**	**1005**	**1040**	**980**	**1165**	**995**	**1105**
**Total Calories: 3865**	**3890**	**3800**	**3855**	**3815**	**3820**	**3820**
**Total Fluid: 1440 mL**	**1440**	**1440**	**1440**	**1440**	**1440**	**1440**

Meals = 800-900 calories (3.5 cans Boost)
Snacks = 500-600 calories (2.5 cans Boost)

4000 kcal Malnutrition Pathway Meal Plan

	MONDAY	TUESDAY	WEDNESDAY	THURSDAY	FRIDAY	SATURDAY	SUNDAY
**Breakfast**							
	12oz whole milk (225)	12oz whole milk (225)	12oz whole milk (225)	12oz whole milk (225)	12oz whole milk (225)	12oz whole milk (225)	12oz whole milk (225)
	1 C oatmeal with 2 pats of butter (215) and 2 oz raisins (130)	breakfast sandwich (2 oz egg on biscuit with 2.5 oz American cheese (495)	bagel (240) with 2 cream cheese packets (200)	5 French toast sticks (325) with syrup (110) and pat butter (35)	3 oz scrambled eggs (135) with 2 oz cheese (150)	3 pancakes (300) with 2 syrups (220) and 3 pats butter (105)	4oz egg omelet with 2 oz American cheese (330)
	peach yogurt, regular (150)	banana (110)	blueberry yogurt (150)	2 sausage patties (360)	1 cup strawberries (60)	turkey bacon, 3 oz (105)	1/2 cup honeydew (30)
	hard boiled egg (75)		watermelon (50)	1/2 cup pineapple (70)	biscuit (220) with 1 pat butter (35)	1 cup watermelon (50)	2 slices wheat toast (140) with 2 pats butter (70) and 2
					1 link turkey sausage (70)		
	**795**	**830**	**865**	**1125**	**895**	**1005**	**895**
**Snack**							
	12oz whole milk (225)	12oz whole milk (225)	12oz whole milk (225)	12oz whole milk (225)	12oz whole milk (225)	12oz whole milk (225)	12oz whole milk (225)
	1/2 cup peaches (50)	Lemon yogurt, regular (150)	blueberry muffin (180) with 1 pat butter (35)	1 cup grapes (60)	2 nature valley granola bars (180)	strawberry yogurt (120)	Cheerios, 2 boxes (150)
	bagel (240)	2 oz granola (120)	1 cup grapes (60)	2 oz cubed cheddar cheese (220)	banana (110)	1/2 cinnamon raisin bagel (125) with pat of butter (35)	banana (110)
	1 packet peanut butter (125)					1 cup strawberries (60)	
	**640**	**495**	**500**	**505**	**515**	**565**	**485**
**Lunch**							
	8oz apple juice (120)	8oz fruit punch (120)	8oz ginger ale (80)	8oz orange juice (120)	8oz grape juice (170)	8oz lemonade (120)	8oz cranberry juice (120)
	Grilled Tilapia with Butter and Herbs, 3.5 oz (130)	10 oz spiral pasta (525) with 3/4 cup marinara sauce (75)	cheeseburger, 2 oz cheese (525) offered with ketchup, lettuce, tomato, pickle	Macaroni and Cheese, 8 oz (460)	grilled cheese (500)	tuna salad sandwich on wheat with 1 oz Swiss cheese (450)	turkey (3 oz) sandwich on wheat with mayo and 2 oz Swiss cheese (580)

Meals = 850-950 calories (4 cans Boost)
Snacks = 500-600 calories (2.5 cans Boost)

4000 kcal Malnutrition Pathway Meal Plan

1/2 cup pineapple (70)	2 meatballs (200)	1/2 cup raw carrots (35)	1/2 cup green beans with 1 pat butter (70)	garden salad (15) with ranch dressing packet (140)	garden side salad (15)	whole apple, sliced (80)
potato stars, 2 oz (180)	1/2 cup green beans (35) with 1 pat butter (35)	Sun Chips, 1.5 oz (210)	apple (80)	fresh fruit cup (50)	Italian dressing (110)	1/2 cup broccoli (20) with pat butter (35)
side caesar salad with ceasar dressing (305)	1/2 cup peaches (50)	ranch dressing packet (140)	sugar cookie (125)	tomato soup (100)	1/2 cup pineapple (70)	hard pretzels (110)
	Rice Krispie Treat (150)	apple (80)				
**805**	**1190**	**1070**	**855**	**975**	**765**	**945**
**Snack**						
8oz apple juice (120)	8oz orange juice (120)	8oz grape juice (170)	8oz cranberry juice (120)	8 oz whole milk (150)	8 oz apple juice (120)	8oz fruit punch (120)
1 cup strawberries (60)	soft pretzel (180)	1/2 cup pears (50)	3 oz hummus (225)	apple, sliced (80)	3 oz baked tortilla chips (270)	1/2 cup pineapple (70)
Tastykake© 3oz Koffee Kake (360)	1 oz cheese sauce (70)	peanut butter and jelly sandwich on wheat (320)	Stacy's © Naked Pita chips, 1.5 oz (200)	6 Nilla wafers (105)	3.8 oz salsa cup (30)	Sweet Street © Pecan Chocolate Chunk bar (300)
			1/2 cup sliced cucmbers (25)	peanut butter packet (125)	1 oz cheese sauce (70)	
**540**	**370**	**540**	**570**	**460**	**490**	**490**
**Dinner**						
8oz whole milk (150)	8oz whole milk (150)	8oz whole milk (150)	8oz whole milk (150)	8 oz Wawa iced tea (90)	8oz whole milk (150)	8oz whole milk (150)
2 grilled chicken breasts, sliced (280)	6 oz roast turkey with 6 oz gravy (450)	chicken alfredo with 3 oz chicken, 6 oz pasta, 4 oz sauce (560)	beef fajitas (255) with sour cream packet (50) and 0.5 oz shredded cheddar cheese (60)	pepperoni pizza (560)	Chicken LoMein with veggies, add extra half chicken breast (420)	4 chicken tenders (400) offered with 2 packets ketchup
1/2 cup cantaloupe (40)	1 cup mashed potatoes (260)	1/2 cup broccoli (20)	1/2 cup cooked carrots (35) with 1 pat butter (35)	1 cup grapes (60)	Brownie cupcake (185), vanilla ice cream cup (140), 2T chocolate syrup (100)	3/4 cup french fries (285) offered with 1 packet ketchup
1/2 cup raw carrot sticks (35)	1/2 cup broccoli with pat of butter (55)	6 oz Cookies & Cream Hershey © ice cream cup (250)	1/2 cup honeydew (30)	1/2 cup broccoli w/1 pat butter (55)	spring roll (200)	1/2 cup green beans (35) with 1 pat butter (35)

Meals = 850-950 calories (4 cans Boost)
Snacks = 500-600 calories (2.5 cans Boost)

4000 kcal Malnutrition Pathway Meal Plan

Baked Potato (160) with 2 pats butter (70)	2 chocolate chip cookies (250)	orange (60)	Milkshake with 4 oz chocolate ice cream, 4 oz whole milk, 3T chocolate syrup (365)	Tastykake© Butter Kreme Snack (3 cupcakes) (400)		Ice cream sandwich (300)
2 Sweet Street © Caramel Apple bar (560)						
**1295**	**1165**	**1040**	**980**	**1165**	**1195**	**1205**
**Total Calories: 4075**	**4050**	**4015**	**4035**	**4010**	**3995**	**4020**
**Total Fluid: 1440 mL**	**1440**	**1440**	**1440**	**1440**	**1440**	**1440**

Meals = 850-950 calories (4 cans Boost)
Snacks = 500-600 calories (2.5 cans Boost)

4200 kcal Malnutrition Pathway Meal Plan

MONDAY	TUESDAY	WEDNESDAY	THURSDAY	FRIDAY	SATURDAY	SUNDAY
**Breakfast**						
12oz whole milk (225)	12oz whole milk (225)	12oz whole milk (225)	12oz whole milk (225)	12oz whole milk (225)	12oz whole milk (225)	12oz whole milk (225)
1 C oatmeal with 2 pats of butter (215) and 2 oz raisins (130)	breakfast sandwich (2 oz egg on biscuit with 2.5 oz American cheese (495)	bagel (240) with 2 cream cheese packets (200)	6 French toast sticks (390) with 2 syrups (220)	3 oz scrambled eggs (135) with 2 oz cheese (150)	3 pancakes (300) with 2 syrups (220) and 3 pats butter (105)	4oz egg omelet with 2 oz American cheese (330)
peach yogurt, regular (150)	banana (110)	blueberry yogurt (150)	2 sausage patties (360)	1 cup strawberries (60)	turkey bacon, 4 oz (140)	1/2 cup honeydew (30)
hard boiled egg (75)		watermelon (50)	1/2 cup pineapple (70)	biscuit (220) with 1 pat butter (35)	1 cup watermelon (50)	2 slices wheat toast (140) with 2 pats butter (70) and 2
				1 link turkey sausage (70)		
**795**	**830**	**865**	**1265**	**895**	**1040**	**895**
**Snack**						
12oz whole milk (225)	12oz whole milk (225)	12oz whole milk (225)	12oz whole milk (225)	12oz whole milk (225)	12oz whole milk (225)	12oz whole milk (225)
1/2 cup peaches (50)	Lemon yogurt, regular (150)	blueberry muffin (180) with 1 pat butter (35)	1 cup grapes (60)	2 nature valley granola bars (180)	strawberry yogurt (120)	Cheerios, 2 boxes (150)
bagel (240)	2 oz granola (120)	1 cup grapes (60)	2 oz cubed cheddar cheese (220)	banana (110)	1/2 cinnamon raisin bagel (125) with pat of butter (35)	banana (110)
1 packet peanut butter (125)					1 cup strawberries (60)	
**640**	**495**	**500**	**505**	**515**	**565**	**485**
**Lunch**						
12oz apple juice (180)	12 oz fruit punch (180)	12oz ginger ale (120)	12 oz orange juice (180)	12 oz grape juice (255)	12oz lemonade (180)	12oz cranberry juice (180)
Grilled Tilapia with Butter and Herbs, 3.5 oz (130)	10 oz spiral pasta (525) with 3/4 cup marinara sauce (75)	cheeseburger, 2 oz cheese (525) offered with ketchup, lettuce, tomato, pickle	Macaroni and Cheese, 8 oz (460)	grilled cheese (500)	tuna salad sandwich on wheat with 1 oz Swiss cheese (450)	turkey (3 oz) sandwich on wheat with mayo and 2 oz Swiss cheese (580)

Meals = 900-1000 calories (4 cans Boost)
Snacks = 550-650 calories (2.5 cans Boost)

4200 kcal Malnutrition Pathway Meal Plan

1/2 cup pineapple (70)	2 meatballs (200)	1/2 cup raw carrots (35)	1/2 cup green beans with 1 pat butter (70)	garden salad (15) with ranch dressing packet (140)	garden side salad (15) with diced hard-boiled egg (75)	whole apple, sliced (80)
potato stars, 3 oz (270)	1/2 cup green beans (35) with 1 pat butter (35)	Sun Chips, 1.5 oz (210)	apple (80)	fresh fruit cup (50)	Italian dressing (110)	1/2 cup broccoli (20) with pat butter (35)
side caesar salad with ceasar dressing (305)	1/2 cup peaches (50)	ranch dressing packet (140)	sugar cookie (125)	tomato soup (100)	1/2 cup pineapple (70)	hard pretzels (110)
	Rice Krispie Treat (150)	apple (80)		goldfish crackers (130)		
**955**	**1250**	**1110**	**915**	**1190**	**900**	**1005**
**Snack**						
8oz apple juice (120)	8oz orange juice (120)	8oz grape juice (170)	8oz cranberry juice (120)	8 oz whole milk (150)	8 oz apple juice (120)	8oz fruit punch (120)
1 cup strawberries (60)	soft pretzel (180)	1/2 cup pears (50)	3 oz hummus (225)	apple, sliced (80)	3 oz baked tortilla chips (270)	1/2 cup pineapple (70)
Tastykake© 3oz Koffee Kake (360)	1 oz cheese sauce (70)	peanut butter and jelly sandwich on wheat (320)	Stacy's © Naked Pita chips, 1.5 oz (200)	6 Nilla wafers (105)	3.8 oz salsa cup (30)	Sweet Street© Pecan Chocolate Chunk bar (300)
			1/2 cup sliced cucmbers (25)	peanut butter packet (125)	1 oz cheese sauce (70)	
**540**	**370**	**540**	**570**	**460**	**490**	**490**
**Dinner**						
8oz whole milk (150)	8oz whole milk (150)	8oz whole milk (150)	8oz whole milk (150)	8 oz Wawa iced tea (90)	8oz whole milk (150)	8oz whole milk (150)
2 grilled chicken breasts, sliced (280)	6 oz roast turkey with 6 oz gravy (450)	chicken alfredo with 4.5 oz chicken, 8 oz pasta, 4 oz sauce (735)	beef fajitas (255) with sour cream packet (50) and 0.5 oz shredded cheddar cheese (60)	pepperoni pizza (560)	Chicken LoMein with veggies, add extra half chicken breast (420)	5 chicken tenders (500) offered with 2 packets ketchup
1/2 cup cantaloupe (40)	1 cup mashed potatoes (260)	1/2 cup broccoli (20)	1/2 cup cooked carrots (35) with 1 pat butter (35)	1 cup grapes (60)	Brownie cupcake (185), vanilla ice cream cup (140), 2T chocolate syrup (100)	3/4 cup french fries (285) offered with 1 packet ketchup
1/2 cup raw carrot sticks (35)	1/2 cup broccoli with pat of butter (55)	6 oz Cookies & Cream Hershey © ice cream cup (250)	1/2 cup honeydew (30)	1/2 cup broccoli w/1 pat butter (55)	spring roll (200)	1/2 cup green beans (35) with 1 pat butter (35)

Meals = 900-1000 calories (4 cans Boost)
Snacks = 550-650 calories (2.5 cans Boost)

4200 kcal Malnutrition Pathway Meal Plan

Baked Potato (160) with 2 pats butter (70)	3 chocolate chip cookies (375)	orange (60)	Milkshake with 4oz chocolate ice cream, 4oz whole milk, 3T chocolate syrup (365)	Tastykake© Butter Kreme Snack (3 cupcakes) (400)		Ice cream sandwich (300)
2 Sweet Street © Caramel Apple bars (560)						
**1295**	**1290**	**1215**	**980**	**1165**	**1195**	**1305**
**Total Calories: 4225**	**4235**	**4230**	**4235**	**4225**	**4190**	**4180**
**Total Fluid: 1560 mL**	**1560**	**1560**	**1560**	**1560**	**1560**	**1560**

Meals = 900-1000 calories (4 cans Boost)
Snacks = 550-650 calories (2.5 cans Boost)

4400 kcal Malnutrition Pathway Meal Plan

MONDAY	TUESDAY	WEDNESDAY	THURSDAY	FRIDAY	SATURDAY	SUNDAY
**Breakfast**						
12oz whole milk (225)	12oz whole milk (225)	12oz whole milk (225)	12oz whole milk (225)	12oz whole milk (225)	12oz whole milk (225)	12oz whole milk (225)
1 C oatmeal with 2 pats of butter (215) with 2 oz raisins (130)	breakfast sandwich (2 oz egg on biscuit with 2.5 oz American cheese (495)	bagel (240) with 2 cream cheese packets (200)	6 French toast sticks (390) with 2 syrups (220)	3 oz scrambled eggs (135) with 2 oz cheese (150)	4 pancakes (400) with 2 syrups (220) and 4 pats butter (140)	4oz egg omelet with 2 oz American cheese (330)
peach yogurt, regular (150)	banana (110)	blueberry yogurt (150) with 2 oz granola (120)	2 sausage patties (360)	1 cup strawberries (60)	turkey bacon, 4 oz (140)	1/2 cup honeydew (30)
2 hard boiled egg (150)		watermelon (50)	1/2 cup pineapple (70)	biscuit (220) with 1 pat butter (35)	1 cup watermelon (50)	3 slices wheat toast (210) with 3 pats butter (105) and 3
				1 link turkey sausage (70)		
**870**	**830**	**985**	**1265**	**895**	**1175**	**1050**
**Snack**						
12 oz whole milk (225)	12 oz whole milk (225)	12 oz whole milk (225)	12 oz whole milk (225)	12 oz whole milk (225)	12 oz whole milk (225)	12 oz whole milk (225)
1/2 cup peaches (50)	Lemon yogurt, regular (150)	blueberry muffin (180) with 1 pat butter (35)	1 cup grapes (60)	2 nature valley granola bars (180)	strawberry yogurt (120)	Cheerios, 2 boxes (150)
bagel (240)	3 oz granola (180)	1 cup grapes (60)	2 oz cubed cheddar cheese (220)	banana (110)	1/2 cinnamon raisin bagel (125) with pat of butter (35)	banana (110)
2 packs cream cheese (200)	1/2 cup honeydew (30)		1 oz trail mix (160)	chocolate pudding (130)	1 cup strawberries (60)	
**715**	**585**	**500**	**665**	**645**	**565**	**485**
**Lunch**						
12oz apple juice (180)	12 oz fruit punch (180)	12oz ginger ale (120)	12 oz orange juice (180)	12 oz grape juice (255)	12oz lemonade (180)	12oz cranberry juice (180)
Grilled Tilapia with Butter and Herbs, 3.5 oz (130)	10 oz spiral pasta (525) with 3/4 cup marinara sauce (75)	cheeseburger, 2 oz cheese (525) offered with ketchup, lettuce, tomato, pickle	Macaroni and Cheese, 8 oz (460)	grilled cheese (500)	tuna salad sandwich on wheat with 1 oz Swiss cheese (450)	turkey (3 oz) sandwich on wheat with mayo and 2 oz Swiss cheese (580)

Meals = 950-1050 calories (4 cans Boost)
Snacks = 600-700 calories (2.5 cans Boost)

4400 kcal Malnutrition Pathway Meal Plan

1/2 cup pineapple (70)	2 meatballs (200)	1/2 cup raw carrots (35)	1/2 cup green beans with 1 pat butter (70)	garden salad (15) with ranch dressing packet (140)	garden side salad (15) with diced hard-boiled egg (75)	whole apple, sliced (80)
potato stars, 3 oz (270)	1/2 cup green beans (35) with 1 pat butter (35)	Sun Chips, 1.5 oz (210)	apple (80)	fresh fruit cup (50)	Italian dressing (110)	1/2 cup broccoli (20) with pat butter (35)
side caesar salad with ceasar dressing (305)	1/2 cup peaches (50)	ranch dressing packet (140)	sugar cookie (125)	tomato soup (100)	1/2 cup pineapple (70)	hard pretzels (110)
	Rice Krispie Treat (150)	apple (80)		goldfish crackers (130)		
**955**	**1250**	**1110**	**915**	**1190**	**900**	**1005**
**Snack**						
12oz apple juice (180)	12oz orange juice (180)	12oz grape juice (255)	12 oz cranberry juice (180)	12 oz whole milk (225)	12 oz apple juice (180)	12oz fruit punch (180)
1 cup strawberries (60)	soft pretzel (180)	1/2 cup pears (50)	3 oz hummus (225)	apple, sliced (80)	3 oz baked tortilla chips (270)	1/2 cup pineapple (70)
Tastykake© 3oz Koffee Kake (360)	1 oz cheese sauce (70)	peanut butter and jelly sandwich on wheat (320)	Stacy's © Naked Pita chips, 1.5 oz (200)	Hershey © Incredible Cone (300)	3.8 oz salsa cup (30)	Sweet Street© Pecan Chocolate Chunk bar (300)
			1/2 cup sliced cucmbers (25)		1 oz cheese sauce (70)	
**600**	**430**	**625**	**630**	**605**	**550**	**550**
**Dinner**						
8oz whole milk (150)	8oz whole milk (150)	8oz whole milk (150)	8oz whole milk (150)	8 oz Wawa iced tea (90)	8oz whole milk (150)	8oz whole milk (150)
2 grilled chicken breasts, sliced (280)	6 oz roast turkey with 6 oz gravy (450)	chicken alfredo with 4.5 oz chicken, 8 oz pasta, 4 oz sauce (735)	beef fajitas (255) with sour cream packet (50) and 0.5 oz shredded cheddar cheese (60)	pepperoni pizza (560)	Chicken LoMein with veggies, add extra half chicken breast (420)	5 chicken tenders (500) offered with 2 packets ketchup
1/2 cup cantaloupe (40)	1 cup mashed potatoes (260)	1/2 cup broccoli (20) with 1 pat butter (35)	1/2 cup cooked carrots (35) with 1 pat butter (35)	1/2 cup broccoli w/1 pat butter (55)	Brownie cupcake (185), vanilla ice cream cup (140), 2T chocolate syrup (100)	1 cup french fries (360) offered with 2 packets ketchup
1/2 cup raw carrot sticks (35)	1/2 cup broccoli with pat of butter (55)	6 oz Cookies & Cream Hershey © ice cream cup (250)	1/2 cup honeydew (30)	Tastykake© Butter Kreme Snack (3 cupcakes) (400)	spring roll (200)	1/2 cup green beans (35) with 1 pat butter (35)

Meals = 950-1050 calories (4 cans Boost)
Snacks = 600-700 calories (2.5 cans Boost)

4400 kcal Malnutrition Pathway Meal Plan

Baked Potato (160) with 2 pats butter (70)	3 chocolate chip cookies (375)	orange (60)	Milkshake with 4 oz chocolate ice cream, 4 oz whole milk, 3T chocolate syrup (365)			Ice cream sandwich (300)
2 Street Sweet © Caramel Apple bar (560)						
**1295**	**1290**	**1250**	**980**	**1105**	**1195**	**1380**
**Total Calories: 4435**	**4385**	**4470**	**4435**	**4440**	**4385**	**4470**
**Total Fluid: 1680 mL**	**1680**	**1680**	**1680**	**1680**	**1680**	**1680**

Meals = 950-1050 calories (4 cans Boost)
Snacks = 600-700 calories (2.5 cans Boost)

4600 kcal Malnutrition Pathway Meal Plan

MONDAY	TUESDAY	WEDNESDAY	THURSDAY	FRIDAY	SATURDAY	SUNDAY
**Breakfast**						
12oz whole milk (225)	12oz whole milk (225)	12oz whole milk (225)	12oz whole milk (225)	12oz whole milk (225)	12oz whole milk (225)	12oz whole milk (225)
1 C oatmeal with 2 pats of butter (215) and 2 oz raisins (130)	breakfast sandwich (2 oz egg on biscuit with 2.5 oz American cheese) (495)	bagel (240)	6 French toast sticks (390) with 2 syrups (220)	3 oz scrambled eggs (135) with 2 oz cheese (150)	4 pancakes (400) with 2 syrups (220) and 4 pats butter (140)	4oz egg omelet with 2 oz American cheese (330)
2 oz raisins (130)	banana (110)	2 cream cheese packets (200)	2 sausage patties (360)	1 cup strawberries (60)	turkey bacon, 4 oz (140)	1/2 cup honeydew (30)
peach yogurt, regular (150)	potato stars, 2 oz (180)	blueberry yogurt (150) with 2 oz granola (120)	1/2 cup pineapple (70)	biscuit (220) with 1 pat butter (35)	1 cup watermelon (50)	3 slices wheat toast (210) with 3 pats butter (105) and 3 packs jelly (150)
2 hard boiled egg (150)		watermelon (50)		1 link turkey sausage (70)		sausage patty (180)
**870**	**1010**	**985**	**1265**	**895**	**1175**	**1230**
**Snack**						
12oz whole milk (225)	12oz whole milk (225)	12oz whole milk (225)	12oz whole milk (225)	12oz whole milk (225)	12oz whole milk (225)	12oz whole milk (225)
1/2 cup peaches (50)	Lemon yogurt, regular (150)	blueberry muffin (180) with 1 pat butter (35)	1 cup grapes (60)	2 nature valley granola bars (180)	strawberry yogurt (120)	Cheerios, 2 boxes (150)
bagel (240)	4 oz granola (240)	1 cup grapes (60)	2 oz cubed cheddar cheese (220)	banana (110)	1/2 cinnamon raisin bagel (125) with pat of butter (35)	banana (110)
2 packs cream cheese (200)	1/2 cup honeydew (30)		1 oz trail mix (160)	chocolate pudding (130)	1 cup strawberries (60)	
**715**	**645**	**500**	**665**	**645**	**565**	**485**
**Lunch**						
12oz apple juice (180)	12 oz fruit punch (180)	12oz ginger ale (120)	12 oz orange juice (180)	12 oz grape juice (255)	12oz lemonade (180)	12oz cranberry juice (180)
Grilled Tilapia with Butter and Herbs, 3.5 oz (130)	10 oz spiral pasta (525) with 3/4 cup marinara sauce (75)	cheeseburger, 2 oz cheese (525) offered with ketchup, lettuce, tomato, pickle	Macaroni and Cheese, 8 oz (460)	grilled cheese (500)	tuna salad sandwich on wheat with 1 oz Swiss cheese (450)	turkey (3 oz) sandwich on wheat with mayo and 2 oz Swiss cheese (580)

Meals = 1000-1100 calories (4.5 cans Boost)
Snacks = 600-700 calories (3 cans Boost)

4600 kcal Malnutrition Pathway Meal Plan

1/2 cup pineapple (70)	2 meatballs (200)	1/2 cup raw carrots (35)	1/2 cup green beans with 1 pat butter (70)	garden salad (15) with ranch dressing packet (140)	garden side salad (15) with diced hard-boiled egg (75)	whole apple, sliced (80)
potato stars, 4 oz (360)	1/2 cup green beans (35) with 1 pat butter (35)	Sun Chips, 3 oz (420)	apple (80)	fresh fruit cup (50)	Italian dressing (110)	1/2 cup broccoli (20) with pat butter (35)
side caesar salad with ceasar dressing (305)	1/2 cup peaches (50)	ranch dressing packet (140)	2 sugar cookies (250)	tomato soup (100)	1/2 cup pineapple (70)	hard pretzels (110)
	Rice Krispie Treat (150)	apple (80)		goldfish crackers (130)	Snickers or Twix Ice Cream Bar (250)	
**1045**	**1250**	**1320**	**1040**	**1190**	**1150**	**1005**
**Snack**						
12oz apple juice (180)	12oz orange juice (180)	12oz grape juice (255)	12 oz cranberry juice (180)	12 oz whole milk (225)	12 oz apple juice (180)	12oz fruit punch (180)
1 cup strawberries (60)	soft pretzel (180)	1/2 cup pears (50)	4 oz hummus (300)	apple, sliced (80)	3 oz baked tortilla chips (270)	1/2 cup pineapple (70)
Tastykake© 3 oz Koffee Kake (360)	1 oz cheese sauce (70)	peanut butter and jelly sandwich on wheat (320)	Stacy's © Naked Pita chips, 1.5 oz (200)	Hershey © Incredible Cone (300)	3.8 oz salsa cup (30)	Sweet Street© Pecan Chocolate Chunk bar (300)
			1/2 cup sliced cucmbers (25)	peanut butter packet (125)	1 oz cheese sauce (70)	
**600**	**430**	**625**	**705**	**730**	**550**	**550**
**Dinner**						
8oz whole milk (150)	8oz whole milk (150)	8oz whole milk (150)	8oz whole milk (150)	8 oz Wawa iced tea (90)	8oz whole milk (150)	8oz whole milk (150)
2 grilled chicken breasts, sliced (280)	6 oz roast turkey with 6 oz gravy (450)	chicken alfredo with 4.5 oz chicken, 8 oz pasta, 4 oz sauce (735)	beef fajitas (255) with sour cream packet (50) and 0.5 oz shredded cheddar cheese (60)	pepperoni pizza (560)	Chicken LoMein with veggies, add extra half chicken breast (420)	5 chicken tenders (500) offered with 3 packets ketchup
1/2 cup cantaloupe (40)	1 cup mashed potatoes (260)	1/2 cup broccoli (20) with 1 pat butter (35)	1/2 cup cooked carrots (35) with 1 pat butter (35)	grapes (60)	Brownie cupcake (185), vanilla ice cream cup (140), 2T chocolate syrup (100)	1 cup french fries (360) offered with 2 packets ketchup
1/2 cup raw carrot sticks (35) with ranch dressing packet (140)	1/2 cup broccoli with pat of butter (55)	6 oz Cookies & Cream Hershey © ice cream cup (250)	1/2 cup honeydew (30)	1/2 cup broccoli w/1 pat butter (55)	spring roll (200)	1/2 cup green beans (35) with 1 pat butter (35)

Meals = 1000-1100 calories (4.5 cans Boost)

Snacks = 600-700 calories (3 cans Boost)

4600 kcal Malnutrition Pathway Meal Plan

Baked Potato (160) with 2 pats butter (70)	3 chocolate chip cookies (375)	orange (60)	Milkshake with 4oz chocolate ice cream, 4oz whole milk, 3T chocolate sauce (365)	Tastykake© Butter Kreme Snack (3 cupcakes) (400)		Ice cream sandwich (300)
2 Sweet Street © Caramel Apple bars (560)						
**1435**	**1290**	**1250**	**980**	**1165**	**1195**	**1380**
**Total Calories: 4665**	**4625**	**4680**	**4655**	**4625**	**4635**	**4650**
**Total Fluid: 1680 mL**	**1680**	**1680**	**1680**	**1680**	**1680**	**1680**

Meals = 1000-1100 calories (4.5 cans Boost)
Snacks = 600-700 calories (3 cans Boost)

4800 kcal Malnutrition Pathway Meal Plan

MONDAY	TUESDAY	WEDNESDAY	THURSDAY	FRIDAY	SATURDAY	SUNDAY
**Breakfast**						
12oz whole milk (225)	12oz whole milk (225)	12oz whole milk (225)	12oz whole milk (225)	12oz whole milk (225)	12oz whole milk (225)	12oz whole milk (225)
1 C oatmeal with 2 pats of butter (215) with 2 oz raisins (130)	breakfast sandwich (2 oz egg on biscuit with 2.5 oz American cheese (495)	bagel (240)	6 French toast sticks (390) with 2 syrups (220)	4 oz scrambled eggs (180) with 2 oz cheese (150)	4 pancakes (400) with 2 syrups (220) and 4 pats butter (140)	4oz egg omelet with 2 oz American cheese (330)
peach yogurt, regular (150) and 2 oz granola (120)	banana (110)	2 cream cheese packets (200)	2 sausage patties (360)	1 cup strawberries (60)	turkey bacon, 4 oz (140)	1/2 cup honeydew (30)
2 hard boiled egg (150)	potato stars, 3 oz (270)	blueberry yogurt (150) with 2 oz granola (120)	1/2 cup pineapple (70)	biscuit (220) with 2 pats butter (70)	1 cup watermelon (50)	3 slices wheat toast (210) with 3 pats butter (105) and 3
		watermelon (50)		2 links turkey sausage (140)		sausage patty (180)
**990**	**1100**	**985**	**1265**	**1045**	**1175**	**1230**
**Snack**						
12oz whole milk (225)	12oz whole milk (225)	12oz whole milk (225)	12oz whole milk (225)	12oz whole milk (225)	12oz whole milk (225)	12oz whole milk (225)
1/2 cup peaches (50)	Lemon yogurt, regular (150) with 4 oz granola (240)	blueberry muffin (180) with 1 pat butter (35)	1 cup grapes (60)	2 nature valley granola bars (180)	strawberry yogurt (120)	Cheerios, 2 boxes (150)
bagel (240) with 2 packs cream cheese (200)	1/2 cup honeydew (30)	1 cup grapes (60)	2 oz cubed cheddar cheese (220)	banana (110)	cinnamon raisin bagel (250) with 2 pats butter (70)	banana (110)
			1 oz trail mix (160)	chocolate pudding (130)	1 cup strawberries (60)	
**715**	**645**	**500**	**665**	**645**	**725**	**485**
**Lunch**						
12oz apple juice (180)	12 oz fruit punch (180)	12oz ginger ale (120)	12 oz orange juice (180)	12 oz grape juice (255)	12oz lemonade (180)	12oz cranberry juice (180)
Grilled Tilapia with Butter and Herbs, 3.5 oz (130)	10 oz spiral pasta (525) with 3/4 cup marinara sauce (75)	cheeseburger, 2 oz cheese (525) offered with ketchup, lettuce, tomato, pickle	Macaroni and Cheese, 8 oz (460)	grilled cheese (500)	tuna salad sandwich on wheat with 1 oz Swiss cheese (450)	turkey (3 oz) sandwich on wheat with mayo and 2 oz Swiss cheese (580)

Meals = 1050-1150 calories (5 cans Boost)
Snacks = 600-700 calories (3 cans Boost)

4800 kcal Malnutrition Pathway Meal Plan

1/2 cup pineapple (70)	3 meatballs (300)	1/2 cup raw carrots (35)	1/2 cup green beans with 1 pat butter (70)	garden salad (15) with ranch dressing packet (140)	garden side salad (15) with diced hard-boiled egg (75)	whole apple, sliced (80)
potato stars, 4 oz (360)	1/2 cup green beans (35) with 1 pat butter (35)	Sun Chips, 3 oz (420)	apple (80) with peanut butter packet (125)	fresh fruit cup (50)	Italian dressing (110)	1/2 cup broccoli (20) with pat butter (35)
side caesar salad with ceasar dressing (305)	1/2 cup peaches (50)	ranch dressing packet (140)	2 sugar cookies (250)	tomato soup (100)	1/2 cup pineapple (70)	soft pretzel (180) offered with mustard pack
	Rice Krispie Treat (150)	apple (80)		goldfish crackers (130)	Snickers or Twix Ice Cream Bar (250)	
**1045**	**1350**	**1320**	**1165**	**1190**	**1150**	**1075**
**Snack**						
12oz apple juice (180)	12oz orange juice (180)	12oz grape juice (255)	12 oz cranberry juice (180)	12 oz whole milk (225)	12 oz apple juice (180)	12oz fruit punch (180)
1 cup strawberries (60)	soft pretzel (180)	1/2 cup pears (50)	4 oz hummus (300)	apple, sliced (80)	3 oz baked tortilla chips (270)	1/2 cup pineapple (70)
Tastykake© 3oz Koffee Kake (360)	1 oz cheese sauce (70)	peanut butter and jelly sandiwch on wheat (320)	Stacy's © Naked Pita chips, 1.5 oz (200)	Hershey © Incredible Cone (300)	3.8 oz salsa cup (30)	Sweet Street© Pecan Chocolate Chunk bar (300)
			1/2 cup sliced cucmbers (25)	peanut butter packet (125)	1 oz cheese sauce (70)	
**600**	**430**	**625**	**705**	**730**	**550**	**550**
**Dinner**						
12 oz whole milk (225)	12oz whole milk (225)	12 oz whole milk (225)	12 oz whole milk (225)	12 oz Wawa iced tea (130)	12oz whole milk (225)	12oz whole milk (225)
2 grilled chicken breasts, sliced (280)	6 oz roast turkey with 6 oz gravy (450)	chicken alfredo with 4.5 oz chicken, 8 oz pasta, 4 oz sauce (735)	beef fajitas (255) with sour cream packet (50) and 0.5 oz shredded cheddar cheese (60)	pepperoni pizza (560)	Chicken LoMein with veggies, add extra half chicken breast (420)	5 chicken tenders (500) offered with 3 packets ketchup
1/2 cup cantaloupe (40)	1 cup mashed potatoes (260)	1/2 cup broccoli (20) with 1 pat butter (35)	1/2 cup cooked carrots (35) with 1 pat butter (35)	1 cup grapes (60)	Brownie cupcake (185), vanilla ice cream cup (140), 2T chocolate syrup (100)	1 cup french fries (360) offered with 2 packets ketchup
1/2 cup raw carrot sticks (35) with ranch dressing (140)	1/2 cup broccoli with pat of butter (55)	6 oz Cookies & Cream Hershey © ice cream cup (250)	1/2 cup honeydew (30)	1/2 cup broccoli w/1 pat butter (55)	spring roll (200)	1/2 cup green beans (35) with 1 pat butter (35)

Meals = 1050-1150 calories (5 cans Boost)
Snacks = 600-700 calories (3 cans Boost)

4800 kcal Malnutrition Pathway Meal Plan

Baked Potato (160) with 2 pats butter (70)	3 chocolate chip cookies (375)	orange (60)	Milkshake with 4 oz chocolate ice cream, 4 oz whole milk, 3T chocolate syrup (365)	Tastykake© Butter Kreme Snack (3 cupcakes) (400)		Ice cream sandwich (300)
2 Sweet Street © Caramel Apple bars (560)		breadstick (110)				
**1510**	**1365**	**1435**	**1055**	**1205**	**1270**	**1455**
**Total Calories: 4860**	**4890**	**4865**	**4855**	**4815**	**4870**	**4795**
**Total Fluid: 1800 mL**	**1800**	**1800**	**1800**	**1800**	**1800**	**1800**

Meals = 1050-1150 calories (5 cans Boost)
Snacks = 600-700 calories (3 cans Boost)

5000 kcal Malnutrition Pathway Meal Plan

MONDAY	TUESDAY	WEDNESDAY	THURSDAY	FRIDAY	SATURDAY	SUNDAY
**Breakfast**						
12oz whole milk (225)	12oz whole milk (225)	12oz whole milk (225)	12oz whole milk (225)	12oz whole milk (225)	12oz whole milk (225)	12oz whole milk (225)
1 C oatmeal with 2 pats of butter (215) with 3 oz raisins (195)	breakfast sandwich (2 oz egg on biscuit with 2.5 oz American cheese (495)	bagel (240) with 2 packets cream cheese (200)	6 French toast sticks (390) with 2 syrups (220)	6 oz scrambled eggs (270) with 3 oz cheese (225)	4 pancakes (400) with 2 syrups (220) and 4 pats butter (140)	4oz egg omelet with 3 oz American cheese (405)
peach yogurt, regular (150) with 1 oz granola (60)	banana (110)	blueberry yogurt (150) with 2 oz granola (120)	2 sausage patties (360)	1 cup strawberries (60)	turkey bacon, 4 oz (140)	1/2 cup honeydew (30)
2 hard boiled egg (150)	potato stars, 3 oz (270)	watermelon (50)	1/2 cup pineapple (70)	biscuit (220) with 2 pats butter (70)	1 cup watermelon (50)	3 slices wheat toast (210) with 3 pats butter (105) and 3
				2 links turkey sausage (140)		2 sausage patties (360)
**995**	**1100**	**985**	**1265**	**1210**	**1175**	**1485**
**Snack**						
12oz whole milk (225)	12oz whole milk (225)	12oz whole milk (225)	12oz whole milk (225)	12oz whole milk (225)	12oz whole milk (225)	12oz whole milk (225)
1/2 cup peaches (50)	Lemon yogurt, regular (150) with 4 oz granola (240)	2 blueberry muffins (360) with 2 pats butter (70)	1 cup grapes (60)	2 nature valley granola bars (180)	strawberry yogurt (120)	Cheerios, 2 boxes (150)
bagel (240) with 2 packs cream cheese (200)	1/2 cup honeydew (30)	1 cup grapes (60)	2 oz cubed cheddar cheese (220)	banana (110)	cinnamon raisin bagel (250) with 2 pats butter (70)	banana (110)
			1 oz trail mix (160)	chocolate pudding (130)	1 cup strawberries (60)	
**715**	**645**	**715**	**665**	**645**	**725**	**485**
**Lunch**						
12oz apple juice (180)	12 oz fruit punch (180)	12oz ginger ale (120)	12 oz orange juice (180)	12 oz grape juice (255)	12oz lemonade (180)	12oz cranberry juice (180)
Grilled Tilapia with Butter and Herbs, 3.5 oz (130)	12 oz spiral pasta (630) with 1 cup marinara sauce (100)	cheeseburger, 2 oz cheese (525) offered with ketchup, lettuce, tomato, pickle	Macaroni and Cheese, 10 oz (575)	grilled cheese (500)	tuna salad sandwich on wheat with 1 oz Swiss cheese (450)	turkey (3 oz) sandwich on wheat with mayo and 2 oz Swiss cheese (580)

Meals = 1050-1150 calories (5 cans Boost)
Snacks = 600-700 calories (3 cans Boost)

5000 kcal Malnutrition Pathway Meal Plan

1/2 cup pineapple (70)	3 meatballs (300)	1/2 cup raw carrots (35)	1/2 cup green beans with 1 pat butter (70)	garden salad (15) with 1.5 ranch dressing packets (210)	garden side salad (15) with diced hard-boiled egg (75)	whole apple, sliced (80)
potato stars, 6 oz (540)	1/2 cup green beans (35) with 1 pat butter (35)	Sun Chips, 3 oz (420)	apple (80) with peanut butter packet (125)	fresh fruit cup (50)	Italian dressing (110)	1/2 cup broccoli (20) with pat butter (35)
side caesar salad with ceasar dressing (305)	1/2 cup peaches (50)	ranch dressing packet (140)	2 sugar cookies (250)	tomato soup (100)	1/2 cup pineapple (70)	soft pretzel (180) offered with mustard pack
	Rice Krispie Treat (150)	apple (80)		goldfish crackers (130)	Snickers or Twix Ice Cream Bar (250)	
**1225**	**1480**	**1320**	**1280**	**1260**	**1150**	**1075**
**Snack**						
12oz apple juice (180)	12oz orange juice (180)	12oz grape juice (255)	12 oz cranberry juice (180)	12 oz whole milk (225)	12 oz apple juice (180)	12oz fruit punch (180)
1 cup strawberries (60)	soft pretzel (180)	1/2 cup pears (50)	4 oz hummus (300)	apple, sliced (80)	3 oz baked tortilla chips (270)	1/2 cup pineapple (70)
Tastykake© 3 oz Koffee Kake (360)	1 oz cheese sauce (70)	peanut butter and jelly sandiwch on wheat (320)	Stacy's © Naked Pita chips, 1.5 oz (200)	Hershey © Incredible Cone (300)	3.8 oz salsa cup (30)	Sweet Street © Pecan Chocolate Chunk bar (300)
			1/2 cup sliced cucmbers (25)	peanut butter packet (125)	1 oz cheese sauce (70)	
**600**	**430**	**625**	**705**	**730**	**550**	**550**
**Dinner**						
12 oz whole milk (225)	12oz whole milk (225)	12 oz whole milk (225)	12 oz whole milk (225)	12 oz Wawa iced tea (130)	12oz whole milk (225)	12oz whole milk (225)
2 grilled chicken breasts, sliced (280)	6 oz roast turkey with 6 oz gravy (450)	chicken alfredo with 4.5 oz chicken, 8 oz pasta, 4 oz sauce (735)	beef fajitas (255) with sour cream packet (50), 0.5 oz shredded cheddar cheese (60), 2 oz avocado (90)	pepperoni pizza (560)	Chicken LoMein with veggies, add extra half chicken breast (420)	5 chicken tenders (500) offered with 3 packets ketchup
1/2 cup cantaloupe (40)	1 cup mashed potatoes (260)	1/2 cup broccoli (20) with 1 pat butter (35)	1/2 cup cooked carrots (35) with 1 pat butter (35)	1 cup grapes (60)	Brownie cupcake (185), vanilla ice cream cup (140), 2T chocolate syrup (100)	1 cup french fries (360) offered with 2 packets ketchup

Meals = 1050-1150 calories (5 cans Boost)
Snacks = 600-700 calories (3 cans Boost)

5000 kcal Malnutrition Pathway Meal Plan

1/2 cup raw carrot sticks (35) with ranch dressing packet (140)	1/2 cup broccoli with pat of butter (55)	6 oz Cookies & Cream Hershey © ice cream cup (250)	1/2 cup honeydew (30)	1/2 cup broccoli w/1 pat butter (55)	spring rolls, 2 (400)	1/2 cup green beans (35) with 1 pat butter (35)
Baked Potato (160) with 2 pats butter (70)	3 chocolate chip cookies (375)	orange (60)	Milkshake with 4 oz chocolate ice cream, 4 oz whole milk, 3T chocolate syrup (365)	Tastykake© Butter Kreme Snack (3 cupcakes) (400)		Ice cream sandwich (300)
2 Sweet Street © Caramel Apple bars (560)		breadstick (110)				
**1510**	**1365**	**1435**	**1145**	**1205**	**1470**	**1455**
**Total Calories: 5045**	**5020**	**5080**	**5060**	**5050**	**5070**	**5050**
**Total Fluid: 1800 mL**	**1800**	**1800**	**1800**	**1800**	**1800**	**1800**

Meals = 1050-1150 calories (5 cans Boost)
Snacks = 600-700 calories (3 cans Boost)

5200 kcal Malnutrition Pathway Meal Plan

MONDAY	TUESDAY	WEDNESDAY	THURSDAY	FRIDAY	SATURDAY	SUNDAY
**Breakfast**						
12oz whole milk (225)	12oz whole milk (225)	12oz whole milk (225)	12oz whole milk (225)	12oz whole milk (225)	12oz whole milk (225)	12oz whole milk (225)
1 C oatmeal with 2 pats of butter (215) with 3 oz raisins (195)	breakfast sandwich (2 oz egg on biscuit with 2.5 oz American cheese (495)	bagel (240) with 2 packets cream cheese (200)	6 French toast sticks (390) with 2 syrups (220)	6 oz scrambled eggs (270) with 4 oz cheese (300)	4 pancakes (400) with 2 syrups (220) and 4 pats butter (140)	4oz egg omelet with 3 oz American cheese (405)
peach yogurt, regular (150) with 1 oz granola (60)	banana (110)	blueberry yogurt (150) with 2 oz granola (120)	2 sausage patties (360)	1 cup strawberries (60)	turkey bacon, 4 oz (140)	1/2 cup honeydew (30)
2 hard boiled egg (150)	potato stars, 5 oz (450)	watermelon (50)	1/2 cup pineapple (70)	biscuit (220) with 2 pats butter (70)	1 cup watermelon (50)	3 slices wheat toast (210) with 3 pats butter (105) and 3 packs jelly (150)
				4 links turkey sausage (280)		2 sausage patties (360)
**995**	**1280**	**985**	**1265**	**1425**	**1175**	**1485**
**Snack**						
12oz whole milk (225)	12oz whole milk (225)	12oz whole milk (225)	12oz whole milk (225)	12oz whole milk (225)	12oz whole milk (225)	12oz whole milk (225)
1/2 cup peaches (50)	Lemon yogurt, regular (150) with 4 oz granola (240)	2 blueberry muffins (360) with 2 pats butter (70)	1 cup grapes (60)	2 nature valley granola bars (180)	strawberry yogurt (120)	Cheerios, 2 boxes (150)
bagel (240) with 2 packs cream cheese (200)	1/2 cup honeydew (30)	1 cup grapes (60)	2 oz cubed cheddar cheese (220)	banana (110)	cinnamon raisin bagel (250) with 2 packs cream cheese (200)	banana (110)
			2 oz trail mix (320)	chocolate pudding (130)	2 oz raisins (130)	
**715**	**645**	**715**	**825**	**645**	**925**	**485**
**Lunch**						
12oz apple juice (180)	12 oz fruit punch (180)	12oz ginger ale (120)	12 oz orange juice (180)	12 oz grape juice (255)	12oz lemonade (180)	12oz cranberry juice (180)

Meals = 1050-1150 calories (5 cans Boost)
Snacks = 600-700 calories (3 cans Boost)

5200 kcal Malnutrition Pathway Meal Plan

Grilled Tilapia with Butter and Herbs, 3.5 oz (130)	12 oz spiral pasta (630) with 1 cup marinara sauce (100)	cheeseburger, 2 oz cheese (525) offered with ketchup, lettuce, tomato, pickle	Macaroni and Cheese, 10 oz (575)	grilled cheese (500)	tuna salad sandwich on wheat with 1 oz Swiss cheese (450)	turkey (3 oz) sandwich on wheat with mayo and 2 oz Swiss cheese (580)
1/2 cup pineapple (70)	3 meatballs (300)	1 cup raw carrots (70)	1/2 cup green beans with 1 pat butter (70)	garden salad (15) with ranch dressing packet (140)	garden side salad (15) with diced hard-boiled egg (75)	whole apple, sliced (80)
potato stars, 8 oz (720)	1/2 cup green beans (35) with 1 pat butter (35)	Sun Chips, 3 oz (420)	apple (80) with peanut butter packet (125)	fresh fruit cup (50)	Italian dressing (110)	1/2 cup broccoli (20) with pat butter (35)
side caesar salad with ceasar dressing (305)	1/2 cup peaches (50)	2 x ranch dressing packet (280)	2 sugar cookies (250)	tomato soup (100)	1/2 cup pineapple (70)	soft pretzel (180)
	Rice Krispie Treat (150)			goldfish crackers (130)	Snickers or Twix Ice Cream Bar (250)	1 oz cheese sauce (70)
**1405**	**1480**	**1415**	**1280**	**1190**	**1150**	**1145**
**Snack**						
12oz apple juice (180)	12oz orange juice (180)	12oz grape juice (255)	12 oz cranberry juice (180)	12 oz whole milk (225)	12 oz apple juice (180)	12oz fruit punch (180)
1 cup strawberries (60)	soft pretzel (180)	5 mozzarella sticks (400) with 4 oz marinara sauce (50)	4 oz hummus (300)	apple, sliced (80)	3 oz baked tortilla chips (270)	1/2 cup pineapple (70)
Tastykake© 3oz Koffee Kake (360)	1 oz cheese sauce (70)		Stacy's © Naked Pita chips, 1.5 oz (200)	Hershey © Incredible Cone (300)	3.8 oz salsa cup (30)	Vanilla yogurt (150)
			1/2 cup sliced cucmbers (25)	peanut butter packet (125)	1 oz cheese sauce (70)	Sweet Street © Pecan chocolate chunk bar (300)
**600**	**430**	**705**	**705**	**730**	**550**	**700**
**Dinner**						
12 oz whole milk (225)	12oz whole milk (225)	12 oz whole milk (225)	12 oz whole milk (225)	12 oz Wawa iced tea (130)	12oz whole milk (225)	12oz whole milk (225)
2 grilled chicken breasts, sliced (280)	6 oz roast turkey with 6 oz gravy (450)	chicken alfredo with 4.5 oz chicken, 8 oz pasta, 4 oz sauce (735)	beef fajitas (255) with sour cream packet (50), 0.5 oz shredded cheddar cheese (60), 2 oz avocado (90)	pepperoni pizza (560)	Chicken LoMein with veggies, add extra half chicken breast (420)	5 chicken tenders (500) offered with 3 packets ketchup

Meals = 1050-1150 calories (5 cans Boost)
Snacks = 600-700 calories (3 cans Boost)

5200 kcal Malnutrition Pathway Meal Plan

1/2 cup cantaloupe (40)	1 cup mashed potatoes (260)	1/2 cup broccoli (20) with 1 pat butter (35)	1/2 cup cooked carrots (35) with 1 pat butter (35)	1 cup grapes (60)	Brownie cupcake (185), vanilla ice cream cup (140), 2T chocolate syrup (100)	1 cup french fries (360) offered with 2 packets ketchup
1/2 cup raw carrot sticks (35) with ranch dressing packet (140)	1/2 cup broccoli with pat of butter (55)	6 oz Cookies & Cream Hershey © ice cream cup (250)	1/2 cup honeydew (30)	1/2 cup broccoli w/1 pat butter (55)	spring rolls, 2 (400)	1/2 cup green beans (35) with 1 pat butter (35)
Baked Potato (160) with 2 pats butter (70)	3 chocolate chip cookies (375)	orange (60)	Milkshake with 4oz chocolate ice cream, 4oz whole milk, 3T chocolate syrup (365)	Tastykake© Butter Kreme Snack (3 cupcakes) (400)		Ice cream sandwich (300)
2 Sweet Street © Caramel Apple bars (560)		breadstick (110)				
**1510**	**1365**	**1435**	**1145**	**1205**	**1470**	**1455**
**Total Calories: 5225**	**5210**	**5255**	**5220**	**5195**	**5270**	**5270**
**Total Fluid: 1800 mL**	**1800**	**1800**	**1800**	**1800**	**1800**	**1800**

Meals = 1050-1150 calories (5 cans Boost)
Snacks = 600-700 calories (3 cans Boost)

5400 kcal Malnutrition Pathway Meal Plan

MONDAY	TUESDAY	WEDNESDAY	THURSDAY	FRIDAY	SATURDAY	SUNDAY
**Breakfast**						
12oz whole milk (225)	12oz whole milk (225)	12oz whole milk (225)	12oz whole milk (225)	12oz whole milk (225)	12oz whole milk (225)	12oz whole milk (225)
1 C oatmeal with 2 pats of butter (215) with 3 oz raisins (195)	breakfast sandwich (2 oz egg on biscuit with 2.5 oz American cheese (495)	bagel (240) with 2 packets cream cheese (200)	6 French toast sticks (390) with 2 syrups (220)	6 oz scrambled eggs (270) with 4 oz cheese (300)	4 pancakes (400) with 2 syrups (220) and 4 pats butter (140)	4oz egg omelet with 3 oz American cheese (405)
peach yogurt, regular (150) with 2 oz granola (120)	banana (110)	blueberry yogurt (150) with 3 oz granola (180)	3 sausage patties (540)	1 cup strawberries (60)	turkey bacon, 4 oz (140)	1/2 cup honeydew (30)
2 hard boiled egg (150)	potato stars, 6 oz (540)	watermelon (50)	1/2 cup pineapple (70)	biscuit (220) with 2 pats butter (70)	1 cup watermelon (50)	3 slices wheat toast (210) with 3 pats butter (105) and 3 packs jelly (150)
				4 links turkey sausage (280)		2 sausage patties (360)
**1055**	**1370**	**1045**	**1445**	**1425**	**1175**	**1485**
**Snack**						
12oz whole milk (225)	12oz whole milk (225)	12oz whole milk (225)	12oz whole milk (225)	12oz whole milk (225)	12oz whole milk (225)	12oz whole milk (225)
1/2 cup peaches (50)	Lemon yogurt, regular (150) with 4 oz granola (240)	2 blueberry muffins (360) with 2 pats butter (70)	1 cup grapes (60)	3 nature valley granola bars (270)	strawberry yogurt (120)	Cheerios, 2 boxes (150)
bagel (240) with 2 packs cream cheese (200)	1/2 cup honeydew (30)	1 cup grapes (60)	2 oz cubed cheddar cheese (220)	banana (110)	cinnamon raisin bagel (250) with 2 packets cream cheese (200)	banana (110)
			2 oz trail mix (320)	chocolate pudding (130)	2 oz raisins (130)	
**715**	**645**	**715**	**825**	**735**	**925**	**485**
**Lunch**						
12oz apple juice (180)	12 oz fruit punch (180)	12oz ginger ale (120)	12 oz orange juice (180)	12 oz grape juice (255)	12oz lemonade (180)	12oz cranberry juice (180)

Meals = 1015-1485 calories (5 cans Boost)
Snacks = 500-800 calories (3 cans Boost)

5400 kcal Malnutrition Pathway Meal Plan

Grilled Tilapia with Butter and Herbs, 3.5 oz (130)	12 oz spiral pasta (630) with 1 cup marinara sauce (100)	cheeseburger, 2 oz cheese (525) offered with ketchup, lettuce, tomato, pickle	Macaroni and Cheese, 10 oz (575)	grilled cheese (500)	tuna salad sandwich on wheat with 1 oz Swiss cheese (450)	turkey (3 oz) sandwich on wheat with mayo and 2 oz Swiss cheese (580)
1 cup pineapple (140)	3 meatball (300)	1 cup raw carrots (70)	1/2 cup green beans with 1 pat butter (70)	garden salad (15) with ranch dressing packet (140)	garden side salad (15) with diced hard-boiled egg (75)	whole apple, sliced (80)
potato stars, 8 oz (720)	1/2 cup green beans (35) with 1 pat butter (35)	Sun Chips, 3 oz (420)	apple (80) with peanut butter packet (125)	fresh fruit cup (50)	Italian dressing (110)	1/2 cup broccoli (20) with pat butter (35)
side caesar salad with ceasar dressing (305)	1/2 cup peaches (50)	2 ranch dressing packets (280)	2 sugar cookies (250)	tomato soup (100)	1/2 cup pineapple (70)	soft pretzel (180)
	Rice Krispie Treat (150)			goldfish crackers (130)	Snickers or Twix Ice Cream Bar (250)	2 oz cheese sauce (140)
**1475**	**1480**	**1415**	**1280**	**1190**	**1150**	**1215**
**Snack**						
12oz apple juice (180)	12oz orange juice (180)	12oz grape juice (255)	12 oz cranberry juice (180)	12 oz whole milk (225)	12 oz apple juice (180)	12oz fruit punch (180)
1 cup strawberries (60)	soft pretzel (180)	5 mozzarella sticks (400) with 4 oz marinara sauce (50)	4 oz hummus (300)	apple, sliced (80)	3 oz baked tortilla chips (270)	1/2 cup pineapple (70)
Tastykake© 3 oz Koffee Kake (360)	2 oz cheese sauce (140)		Stacy's © Naked Pita chips, 1.5 oz (200)	Hershey 6oz Dulce de Leche ice cream cup (270), 2T chocolate syrup (100)	3.8 oz salsa cup (30)	Vanilla yogurt (150)
			1/2 cup sliced cucmbers (25)	peanut butter packet (125)	1 oz cheese sauce (70)	Sweet Street© Pecan Chocolate Chunk bar (300)
**640**	**500**	**705**	**705**	**800**	**550**	**700**
**Dinner**						
12 oz whole milk (225)	12oz whole milk (225)	12 oz whole milk (225)	12 oz whole milk (225)	12 oz Wawa iced tea (130)	12oz whole milk (225)	12oz whole milk (225)

Meals = 1015-1485 calories (5 cans Boost)
Snacks = 500-800 calories (3 cans Boost)

5400 kcal Malnutrition Pathway Meal Plan

2 grilled chicken breasts, sliced (280)	6 oz roast turkey with 6 oz gravy (450)	chicken alfredo with 4.5 oz chicken, 8 oz pasta, 4 oz sauce (735)	beef fajitas (255) with sour cream packet (50), 0.5 oz shredded cheddar cheese (60), 2 oz avocado (90)	pepperoni pizza (560)	Chicken LoMein with veggies, add extra half chicken breast (420)	6 chicken tenders (600) offered with 3 packets ketchup
1/2 cup cantaloupe (40)	1 cup mashed potatoes (260) with 1 pat butter (35)	1/2 cup broccoli (20) with 1 pat butter (35)	1/2 cup cooked carrots (35) with 1 pat butter (35)	1 cup grapes (60)	Brownie cupcake (185), vanilla ice cream cup (140), 2T chocolate syrup (100)	1 cup french fries (360) offered with 2 packets ketchup
1/2 cup raw carrot sticks (35) with ranch dressing packet (140)	1/2 cup broccoli with pat of butter (55)	6 oz Hershey© Cookies & Cream ice cream cup (250) with 2T chocolate syrup (100)	1/2 cup honeydew (30)	1/2 cup broccoli w/1 pat butter (55)	3 spring rolls (600)	1/2 cup green beans (35) with 1 pat butter (35)
Baked Potato (160) with 3 pats butter (105)	3 chocolate chip cookies (375)	orange (60)	Milkshake with 4 oz chocolate ice cream, 4 oz whole milk, 3T chocolate syrup	Tastkake© Butter Kreme snack (3 cupcakes) (400)		Ice cream sandwich (300)
2 Sweet Street © Caramel Apple bars (560)		breadstick (110)				
**1545**	**1400**	**1535**	**1145**	**1205**	**1670**	**1555**
**Total Calories: 5430**	**5395**	**5425**	**5400**	**5355**	**5470**	**5440**
**Total Fluid: 1800 mL**	**1800**	**1800**	**1800**	**1800**	**1800**	**1800**

Meals = 1015-1485 calories (5 cans Boost)
Snacks = 500-800 calories (3 cans Boost)

5600 kcal Malnutrition Pathway Meal Plan

MONDAY	TUESDAY	WEDNESDAY	THURSDAY	FRIDAY	SATURDAY	SUNDAY
**Breakfast**						
12oz whole milk (225)	12oz whole milk (225)	12oz whole milk (225)	12oz whole milk (225)	12oz whole milk (225)	12oz whole milk (225)	12oz whole milk (225)
1 C oatmeal with 3 pats of butter (250) with 4 oz raisins (260)	breakfast sandwich (2 oz egg on biscuit with 2.5 oz American cheese (495)	bagel (240) with 2 packets cream cheese (200)	6 French toast sticks (390) with 2 syrups (220)	6 oz scrambled eggs (270) with 4 oz cheese (300)	4 pancakes (400) with 2 syrups (220) and 4 pats butter (140)	4oz egg omelet with 4 oz American cheese (475)
peach yogurt, regular (150) with 3 oz granola (180)	banana (110)	blueberry yogurt (150) with 4 oz granola (240)	3 sausage patties (540)	1 cup strawberries (60)	turkey bacon, 4 oz (140)	1/2 cup honeydew (30)
2 hard boiled egg (150)	potato stars, 6 oz (540)	watermelon (50)	1/2 cup pineapple (70)	biscuit (220) with 2 pats butter (70)	1 cup watermelon (50)	3 slices wheat toast (210) with 3 pats butter (105) and 3 packs jelly (150)
				4 links turkey sausage (280)		2 sausage patties (360)
**1215**	**1370**	**1105**	**1445**	**1425**	**1175**	**1555**
**Snack**						
12oz whole milk (225)	12oz whole milk (225)	12oz whole milk (225)	12oz whole milk (225)	12oz whole milk (225)	12oz whole milk (225)	12oz whole milk (225)
1/2 cup peaches (50)	Lemon yogurt, regular (150) with 4 oz granola (240)	2 blueberry muffins (360) with 2 pats butter (70)	1 cup grapes (60)	3 nature valley granola bars (270)	strawberry yogurt (120)	Cheerios, 2 boxes (150)
bagel (240) with 2 packs cream cheese (200)	1/2 cup honeydew (30)	1 cup grapes (60)	3 oz cubed cheddar cheese (330)	banana (110)	cinnamon raisin bagel (250) with 2 packets cream cheese (200)	banana (110)
			2 oz trail mix (320)	chocolate pudding (130)	3 oz raisins (195)	
				2 packets peanut butter (250)		
**715**	**645**	**715**	**935**	**985**	**990**	**485**
**Lunch**						
12oz apple juice (180)	12 oz fruit punch (180)	12oz ginger ale (120)	12 oz orange juice (180)	12 oz grape juice (255)	12oz lemonade (180)	12oz cranberry juice (180)

Meals = 1050-1150 calories (5 cans Boost)
Snacks = 600-700 calories (3 cans Boost)

5600 kcal Malnutrition Pathway Meal Plan

Grilled Tilapia with Butter and Herbs, 3.5 oz (130)	12 oz spiral pasta (630) with 1 cup marinara sauce (100)	cheeseburger, 2 oz cheese (525) offered with ketchup, lettuce, tomato, pickle	Macaroni and Cheese, 10 oz (575)	grilled cheese (500)	tuna salad sandwich on wheat with 2 oz Swiss cheese (560)	turkey (3 oz) sandwich on wheat with mayo and 2 oz Swiss cheese (580)
1 cup pineapple (140)	5 meatballs (500)	1 cup raw carrots (70)	1/2 cup green beans with 1 pat butter (70)	garden salad (15) with ranch dressing packet (140)	garden side salad (15) with diced hard-boiled egg (75)	whole apple, sliced (80)
potato stars, 8 oz (720)	1/2 cup green beans (35) with 1 pat butter (35)	Sun Chips, 3 oz (420)	apple (80) with peanut butter packet (125)	fresh fruit cup (50)	Italian dressing (110)	1/2 cup broccoli (20) with pat butter (35)
side caesar salad with ceasar dressing (305)	1/2 cup peaches (50)	2 ranch dressing packets (280)	3 sugar cookies (375)	tomato soup (100)	1/2 cup pineapple (70)	soft pretzel (180)
	Rice Krispie Treat (150)			goldfish crackers (130)	Snickers or Twix Ice Cream Bar (250)	2 oz cheese sauce (140)
**1475**	**1680**	**1415**	**1405**	**1190**	**1260**	**1215**
**Snack**						
12oz apple juice (180)	12oz orange juice (180)	12oz grape juice (255)	12 oz cranberry juice (180)	12 oz whole milk (225)	12 oz apple juice (180)	12oz fruit punch (180)
1 cup strawberries (60)	soft pretzel (180)	5 mozzarella sticks (400) with 4 oz marinara sauce (50)	4 oz hummus (300)	apple, sliced (80)	3 oz baked tortilla chips (270)	Milkshake with 8 oz vanilla ice cream, 6 oz whole milk, 1T chocolate syrup (640)
Tastykake© 3oz Koffee Kake (360)	2 oz cheese sauce (140)		Stacy's © Naked Pita chips, 1.5 oz (200)	Hershey© 6oz Dulce de Leche ice cream cup (270), 2T chocolate syrup (100)	3.8 oz salsa cup (30)	
			1/2 cup sliced cucmbers (25)	peanut butter packet (125)	1 oz cheese sauce (70)	
**640**	**500**	**705**	**705**	**800**	**550**	**820**
**Dinner**						
12 oz whole milk (225)	12oz whole milk (225)	12 oz whole milk (225)	12 oz whole milk (225)	12 oz Wawa iced tea (130)	12oz whole milk (225)	12oz whole milk (225)

Meals = 1050-1150 calories (5 cans Boost)
Snacks = 600-700 calories (3 cans Boost)

5600 kcal Malnutrition Pathway Meal Plan

2 grilled chicken breasts, sliced (280)	6 oz roast turkey with 6 oz gravy (450)	chicken alfredo with 4.5 oz chicken, 8 oz pasta, 4 oz sauce (735)	beef fajitas (255) with sour cream packet (50), 0.5 oz shredded cheddar cheese (60), 2 oz avocado (90)	pepperoni pizza (560)	Chicken LoMein with veggies, add extra half chicken breast (420)	6 chicken tenders (600) offered with 3 packets ketchup
1/2 cup cantaloupe (40)	1 cup mashed potatoes (260) with 1 pat butter (35)	1/2 cup broccoli (20) with 1 pat butter (35)	1/2 cup cooked carrots (35) with 1 pat butter (35)	1 cup grapes (60)	Brownie cupcake (185), vanilla ice cream cup (140), 2T chocolate syrup (100)	1 cup french fries (360) offered with 2 packets ketchup
1/2 cup raw carrot sticks (35) with ranch dressing packet (140)	1/2 cup broccoli with pat of butter (55)	6 oz Hershey© Cookies & Cream ice cream cup (250), 2T chocolate syrup (100)	1/2 cup honeydew (30)	1/2 cup broccoli w/1 pat butter (55)	3 spring rolls (600)	1/2 cup green beans (35) with 1 pat butter (35)
Baked Potato (160) with 3 pats butter (105)	3 chocolate chip cookies (375)	orange (60)	chocolate ice cream, 4 oz whole milk, 3T chocolate syrup (365)	Tastykake© Butter Kreme Snack (3 cupcakes) (400)		Ice cream sandwich (300)
2 Sweet Street © Caramel Apple bars (560)		2 breadsticks (220)				
**1545**	**1400**	**1645**	**1145**	**1205**	**1670**	**1555**
**Total Calories: 5590**	**5595**	**5585**	**5635**	**5605**	**5645**	**5630**
**Total Fluid: 1800 mL**	**1800**	**1800**	**1800**	**1800**	**1800**	**1800**

Meals = 1050-1150 calories (5 cans Boost)
Snacks = 600-700 calories (3 cans Boost)

5800 kcal Malnutrition Pathway Meal Plan

MONDAY	TUESDAY	WEDNESDAY	THURSDAY	FRIDAY	SATURDAY	SUNDAY
**Breakfast**						
12oz whole milk (225)	12oz whole milk (225)	12oz whole milk (225)	12oz whole milk (225)	12oz whole milk (225)	12oz whole milk (225)	12oz whole milk (225)
1 C oatmeal with 3 pats of butter (250) with 4 oz raisins (260)	breakfast sandwich (2 oz egg on biscuit with 2.5 oz American cheese (495)	bagel (240) with 2 packets cream cheese (200)	6 French toast sticks (390) with 2 syrups (220)	6 oz scrambled eggs (270) with 4 oz cheese (300)	4 pancakes (400) with 2 syrups (220) and 4 pats butter (140)	4oz egg omelet with 4 oz American cheese (475)
peach yogurt, regular (150) with 3 oz granola (180)	banana (110)	blueberry yogurt (150) with 4 oz granola (240)	3 sausage patties (540)	1 cup strawberries (60)	turkey bacon, 4 oz (140)	1/2 cup honeydew (30)
2 hard boiled egg (150)	potato stars, 6 oz (540)	watermelon (50)	1/2 cup pineapple (70)	biscuit (220) with 2 pats butter (70)	1 cup watermelon (50)	3 slices wheat toast (210) with 3 pats butter (105) and 3 packs jelly (150)
				4 links turkey sausage (280)		2 sausage patties (360)
**1215**	**1370**	**1105**	**1445**	**1425**	**1175**	**1555**
**Snack**						
12oz whole milk (225)	12oz whole milk (225)	12oz whole milk (225)	12oz whole milk (225)	12oz whole milk (225)	12oz whole milk (225)	12oz whole milk (225)
1 cup peaches (100)	Lemon yogurt, regular (150) with 4 oz granola (240)	2 blueberry muffins (360) with 2 pats butter (70)	1 cup grapes (60)	3 nature valley granola bars (270)	strawberry yogurt (120)	Cheerios, 2 boxes (150)
bagel (240) with 2 packs cream cheese (200)	1/2 cup honeydew (30)	1 cup grapes (60)	3 oz cubed cheddar cheese (330)	banana (110)	cinnamon raisin bagel (250) with 2 packets cream cheese (200)	banana (110)
	2 sugar cookies (250)		2 oz trail mix (320)	chocolate pudding (130)	3 oz raisins (195)	Peanut butter packet (125)
				2 packets peanut butter (250)		
**765**	**895**	**715**	**935**	**985**	**990**	**610**
**Lunch**						
12oz apple juice (180)	12 oz fruit punch (180)	12oz ginger ale (120)	12 oz orange juice (180)	12 oz grape juice (255)	12oz lemonade (180)	12oz cranberry juice (180)

Meals = 1050-1150 calories (5 cans Boost)
Snacks = 600-700 calories (3 cans Boost)

5800 kcal Malnutrition Pathway Meal Plan

Grilled Tilapia with Butter and Herbs, 3.5 oz (130)	12 oz spiral pasta (630) with 1 cup marinara sauce (100)	cheeseburger, 3 oz cheese (600) offered with ketchup, lettuce, tomato, pickle	Macaroni and Cheese, 10 oz (575)	grilled cheese (500)	tuna salad sandwich on wheat with 2 oz Swiss cheese (560)	turkey (3 oz) sandwich on wheat with mayo and 2 oz Swiss cheese (580)
1 cup pineapple (140)	5 meatball (500)	1 cup raw carrots (70)	1/2 cup green beans with 1 pat butter (70)	garden salad (15) with ranch dressing packet (140)	garden side salad (15) with 2 diced hard-boiled eggs (150)	whole apple, sliced (80)
potato stars, 8 oz (720)	1/2 cup green beans (35) with 1 pat butter (35)	Sun Chips, 3 oz (420)	apple (80) with peanut butter packet (125)	fresh fruit cup (50)	Italian dressing (110)	1/2 cup broccoli (20) with pat butter (35)
side caesar salad with ceasar dressing (305)	1/2 cup peaches (50)	2 x ranch dressing packet (280)	3 sugar cookies (375)	tomato soup (100)	1/2 cup pineapple (70)	soft pretzel (180)
	Rice Krispie Treat (150)	Chocolate ice cream (140)		goldfish crackers (130)	Snickers or Twix Ice Cream Bar (250)	2 oz cheese sauce (140)
**1475**	**1680**	**1630**	**1405**	**1190**	**1335**	**1215**
**Snack**						
12oz grape juice (255)	12oz orange juice (180)	12oz grape juice (255)	12 oz cranberry juice (180)	12 oz whole milk (225)	12 oz apple juice (180)	12oz fruit punch (180)
1 cup strawberries (60)	soft pretzel (180)	5 mozzarella sticks (400) with 4 oz marinara sauce (50)	4 oz hummus (300)	apple, sliced (80)	3 oz baked tortilla chips (270)	Milkshake with 8 oz vanilla ice cream, 6 oz whole milk, 2T chocolate syrup (690)
Tastykake© 3 oz Koffee Kake (360)	2 oz cheese sauce (140)		Stacy's © Naked Pita chips, 1.5 oz (200)	Hershey© Dulce de Leche ice cream cup (270), 2T chocolate syrup (100)	3.8 oz salsa cup (30)	
			1/2 cup sliced cucmbers (25)	peanut butter packet (125)	2 oz cheese sauce (140)	
**715**	**500**	**705**	**705**	**800**	**620**	**870**
**Dinner**						
12 oz whole milk (225)	12oz whole milk (225)	12 oz whole milk (225)	12 oz whole milk (225)	12 oz whole milk (225)	12oz whole milk (225)	12oz whole milk (225)

Meals = 1050-1150 calories (5 cans Boost)
Snacks = 600-700 calories (3 cans Boost)

5800 kcal Malnutrition Pathway Meal Plan

2.5 grilled chicken breasts, sliced (350)	6 oz roast turkey with 6 oz gravy (450)	chicken alfredo with 4.5 oz chicken, 8 oz pasta, 4 oz sauce (735)	beef fajitas (255) with sour cream packet (50), 2 oz shredded cheddar cheese (220), 2 oz avocado (90)	pepperoni pizza (560)	Chicken LoMein with veggies, add extra chicken breast (490)	6 chicken tenders (600) offered with 3 packets ketchup
1/2 cup cantaloupe (40)	1 cup mashed potatoes (260) with 1 pat butter (35)	1/2 cup broccoli (20) with 1 pat butter (35)	1/2 cup cooked carrots (35) with 1 pat butter (35)	1 cup grapes (60)	Brownie cupcake (185), vanilla ice cream cup (140), 2T chocolate syrup (100)	1 cup french fries (360) offered with 2 packets ketchup
1/2 cup raw carrot sticks (35) with ranch dressing packet (140)	1/2 cup broccoli with pat of butter (55)	6 oz Hershey© Cookies & Cream Ice Cream Cup (250), 2T chocolate syrup (100)	1/2 cup honeydew (30)	1/2 cup broccoli w/1 pat butter (55)	3 spring rolls (600)	1/2 cup green beans (35) with 1 pat butter (35)
Baked Potato (160) with 3 pats butter (105), 1 oz sour cream (50)	3 chocolate chip cookies (375)	orange (60)	Milkshake with 4 oz chocolate ice cream, 4 oz whole milk, 3T chocolate syrup (365)	Tastykake© Butter Kreme Snack (3 cupcakes) (400)		Ice cream sandwich (300)
2 Sweet Street © Caramel Apple bars (560)		2 breadsticks (220)		1 breadstick (110)		
**1665**	**1400**	**1645**	**1305**	**1410**	**1740**	**1555**
**Total Calories: 5835**	**5845**	**5800**	**5795**	**5810**	**5860**	**5805**
**Total Fluid: 1800 mL**	**1800**	**1800**	**1800**	**1800**	**1800**	**1800**

Meals = 1050-1150 calories (5 cans Boost)
Snacks = 600-700 calories (3 cans Boost)

6000 kcal Malnutrition Pathway Meal Plan

MONDAY	TUESDAY	WEDNESDAY	THURSDAY	FRIDAY	SATURDAY	SUNDAY
**Breakfast**						
12oz whole milk (225)	12oz whole milk (225)	12oz whole milk (225)	12oz whole milk (225)	12oz whole milk (225)	12oz whole milk (225)	12oz whole milk (225)
1 C oatmeal with 3 pats of butter (250) with 4 oz raisins (260)	breakfast sandwich (2 oz egg on biscuit with 2.5 oz American cheese (495)	bagel (240) with 2 packets cream cheese (200)	8 French toast sticks (520) with 2 syrups (220)	6 oz scrambled eggs (270) with 4 oz cheese (300)	4 pancakes (400) with 2 syrups (220) and 4 pats butter (140)	4oz egg omelet with 4 oz American cheese (475)
peach yogurt, regular (150) with 3 oz granola (180)	banana (110)	blueberry yogurt (150) with 4 oz granola (240)	3 sausage patties (540)	1 cup strawberries (60)	turkey bacon, 4 oz (140)	1/2 cup honeydew (30)
2 hard boiled egg (150)	potato stars, 6 oz (540)	watermelon (50)	1/2 cup pineapple (70)	biscuit (220) with 2 pats butter (70)	1 cup watermelon (50)	3 slices wheat toast (210) with 3 pats butter (105) and 3 packs jelly (150)
				4 links turkey sausage (280)	Cinnamon bun (160)	3 sausage patties (540)
**1215**	**1370**	**1105**	**1575**	**1425**	**1335**	**1735**
**Snack**						
12oz whole milk (225)	12oz whole milk (225)	12oz whole milk (225)	12oz whole milk (225)	12oz whole milk (225)	12oz whole milk (225)	12oz whole milk (225)
1 cup peaches (100)	Lemon yogurt, regular (150) with 4 oz granola (240)	2 blueberry muffins (360) with 2 pats butter (70)	1 cup grapes (60)	3 nature valley granola bars (270)	strawberry yogurt (120)	Cheerios, 2 boxes (150)
bagel (240) with 2 packs cream cheese (200)	1/2 cup honeydew (30)	1 cup grapes (60)	3 oz cubed cheddar cheese (330)	banana (110)	cinnamon raisin bagel (250) with 2 packets cream cheese (200)	banana (110)
	2 sugar cookies (250)		2 oz trail mix (320)	chocolate pudding (130)	4 oz granola (240)	Peanut butter packet (125)
				2 packets peanut butter (250)		
**765**	**895**	**715**	**935**	**985**	**1035**	**610**
**Lunch**						
12oz apple juice (180)	12 oz fruit punch (180)	12oz ginger ale (120)	12 oz orange juice (180)	12 oz grape juice (255)	12oz lemonade (180)	12oz cranberry juice (180)

Meals = 1050-1150 calories (5 cans Boost)

Snacks = 600-700 calories (3 cans Boost)

6000 kcal Malnutrition Pathway Meal Plan

Grilled Tilapia with Butter and Herbs, 3.5 oz (130)	12 oz spiral pasta (630) with 1 cup marinara sauce (100)	cheeseburger, 3 oz cheese (600) offered with ketchup, lettuce, tomato, pickle	Macaroni and Cheese, 10 oz (575)	grilled cheese (500)	tuna salad sandwich on wheat with 2 oz Swiss cheese (560)	turkey (3 oz) sandwich on wheat with mayo and 2 oz Swiss cheese (580)
1 cup pineapple (140)	5 meatball (500)	1 cup raw carrots (70)	1/2 cup green beans with 1 pat butter (70)	garden salad (15) with ranch dressing packet (140)	garden side salad (15) with 2 diced hard-boiled eggs (150)	whole apple, sliced (80)
potato stars, 8 oz (720)	1/2 cup green beans (35) with 1 pat butter (35)	Sun Chips, 3 oz (420)	apple (80) with peanut butter packet (125)	fresh fruit cup (50)	Italian dressing (110)	1/2 cup broccoli (20) with pat butter (35)
side caesar salad with ceasar dressing (305)	1/2 cup peaches (50)	2 x ranch dressing packet (280)	3 sugar cookies (375)	tomato soup (100)	1/2 cup pineapple (70)	soft pretzel (180)
	Rice Krispie Treat (150)	Sweet Street© Lemonberry Jazz Bar (340)		2 bags goldfish crackers (260)	Snickers or Twix Ice Cream Bar (250)	2 oz cheese sauce (140)
**1475**	**1680**	**1830**	**1405**	**1320**	**1335**	**1215**
**Snack**						
12oz grape juice (255)	12oz orange juice (180)	12oz grape juice (255)	12 oz cranberry juice (180)	12 oz whole milk (225)	12 oz apple juice (180)	12oz fruit punch (180)
1 cup strawberries (60)	soft pretzel (180)	5 mozzarella sticks (400) with 4 oz marinara sauce (50)	4 oz hummus (300)	apple, sliced (80)	3 oz baked tortilla chips (270)	Milkshake with 8 oz vanilla ice cream, 6 oz whole milk, 2T chocolate syrup (690)
Tastykake© 3oz Koffee Kake (360)	2 oz cheese sauce (140)		Stacy's © Naked Pita chips, 1.5 oz (200)	Hershey© 6oz Dulce de Leche ice cream cup (270), 2T chocolate syrup (100)	3.8 oz salsa cup (30)	
	Vanilla pudding (130)		1/2 cup sliced cucmbers (25)	peanut butter packet (125)	2 oz cheese sauce (140)	
**715**	**630**	**705**	**705**	**800**	**620**	**870**
**Dinner**						
12 oz whole milk (225)	12oz whole milk (225)	12 oz whole milk (225)	12 oz whole milk (225)	12 oz whole milk (225)	12oz whole milk (225)	12oz whole milk (225)

Meals = 1050-1150 calories (5 cans Boost)
Snacks = 600-700 calories (3 cans Boost)

6000 kcal Malnutrition Pathway Meal Plan

2.5 grilled chicken breasts, sliced (350)	6 oz roast turkey with 6 oz gravy (450)	chicken alfredo with 4.5 oz chicken, 8 oz pasta, 4 oz sauce (735)	beef fajitas (255) with 2 sour cream packets (100), 2 oz shredded cheddar cheese (220), 2 oz avocado (90)	pepperoni pizza (560)	Chicken LoMein with veggies, add extra chicken breast (490)	6 chicken tenders (600) offered with 3 packets ketchup
1/2 cup cantaloupe (40)	1 cup mashed potatoes (260) with 3 pats butter (105)	1/2 cup broccoli (20) with 1 pat butter (35)	1/2 cup cooked carrots (35) with 1 pat butter (35)	1 cup grapes (60)	Brownie cupcake (185), vanilla ice cream cup (140), 2T chocolate syrup (100)	1 cup french fries (360) offered with 2 packets ketchup
1/2 cup raw carrot sticks (35) with ranch dressing packet (140)	1/2 cup broccoli with pat of butter (55)	6 oz Hershey Cookies & Cream ice cream cup (250) with 2T Hershey syrup (100)	1/2 cup honeydew (30)	1 cup broccoli w/2 pats butter (110)	3 spring rolls (600)	1/2 cup green beans (35) with 1 pat butter (35)
Baked Potato (160) with 3 pats butter (105), 2 oz sour cream (100), 1 oz shredded cheddar (110)	3 chocolate chip cookies (375)	orange (60)	Milkshake with 4oz chocolate ice cream, 4 oz whole milk, 3T chocolate syrup (365)	Tastykake© Butter Kreme Snack (3 cupcakes) (400)		Ice cream sandwich (300)
2 Sweet Street © Caramel Apple bars (560)		2 breadsticks (220)		1 breadstick (110)		
**1825**	**1470**	**1645**	**1355**	**1465**	**1740**	**1555**
**Total Calories: 5995**	**6045**	**6000**	**5975**	**5995**	**6065**	**5985**
**Total Fluid: 1800 mL**	**1800**	**1800**	**1800**	**1800**	**1800**	**1800**

Meals = 1050-1150 calories (5 cans Boost)
Snacks = 600-700 calories (3 cans Boost)

Malnutrition Pathway Menu Modifications

<u>Table of Contents</u>

1. NO Citrus
2. NO Dairy
3. NO Whole egg
4. NO Fish
5. Gluten-free
6. Halal
7. Kosher
8. NO milk/meat together in meal (non-kosher meats)
9. NO nuts
10. NO red meat
11. Vegetarian – Pescetarian
12. Vegetarian – Lacto-ovo Vegetarian

Malnutrition Pathway Menu Modifications

1. **Citrus**

No citrus foods, please.
Replace orange (60) with strawberries (60) or grapes (60).
Replace orange juice (120, 180) with apple juice (120, 180) or cranberry juice (120, 180).
Replace lemonade (120, 180) with fruit punch (120, 180)

Malnutrition Pathway Menu Modifications

2. **<u>Dairy</u>**

Please replace all milk with soy milk.
- Replace 8 oz 2% milk with 10 oz soy milk
- Replace 8 oz whole milk with 10 oz soy milk
- Replace 12 oz whole milk 16 oz soy milk

Replace 1 pat butter with 2 pats smart balance (30)

Mon:
B -
- replace light strawberry yogurt (90) with 2 oz bacon (70)
- replace peach yogurt (150) with sausage patty (180)
- replace peach yogurt (150) and 1 oz granola (60) with 3 turkey sausage links (210)
- replace peach yogurt (150) and 2 oz granola (120) with 4 turkey sausage links (280)
- replace peach yogurt (150) and 3 oz granola (180) with 5 pieces bacon (350)

10S – replace 2 packs cream cheese (200) with 2 peanut butter packets (150)
L –
- replace Caesar salad/half dressing packet (185) with 1 cup peas (200)
- replace Caesar salad/whole dressing packet (305) with and 1 cup peas (200) with 1 pat Smart Balance (20) and send 1 extra oz tater tots (90)

3S – replace vanilla pudding with chocolate chip cookie (125)
D –
- replace ranch dressing with Italian dressing (110)
- replace 1 oz sour cream (50) with additional ½ cup cantaloupe (40)

Tues:
B - no breakfast sandwich.
- Replace breakfast sandwich (165) with 1 blueberry pancake (110) and ½ packet syrup (55)
- Replace breakfast sandwich (240) with 1 blueberry pancake (110) and syrup (110) and pat butter (35)
- Replace breakfast sandwich (315) with 2 blueberry pancakes (220), syrup (110) and butter (35)
- Replace breakfast sandwich (415) with 3 blueberry pancakes (330), syrup (110) and butter (35)
- Replace breakfast sandwich (495) with 3 blueberry pancakes (330), 2 syrup (110) and 2 butter (70)

10S –
- Replace light strawberry yogurt (90) with one nature valley bar (90)
- Replace lemon yogurt (150) and 2 oz granola (120) with cinnamon bun (160) and banana (110)
- Replace lemon yogurt (150) and 3 oz granola (180) with Lemonberry Jazz bar (340)
- Replace lemon yogurt (150) and 4 oz granola (240) with Lemonberry Jazz bar (340)

3S –

Malnutrition Pathway Menu Modifications

- Replace soft pretzel (180) and 1 oz cheese sauce (70) with corn muffin (220)
- Replace soft pretzel (180) and 2 oz cheese sauce (140) with Tastykake © Chocolate Junior (330)
- Replace soft pretzel (180) and 2 oz cheese sauce (140) and vanilla pudding (130) with Tastykake © Apple Pie (470)

Wed:
B –

- Replace cream cheese with peanut butter (125)
- Replace 2 cream cheese packets (200) with 2 peanut butter packets (250)
- Replace blueberry yogurt (150) with 2 turkey sausage links (140)
- Replace blueberry yogurt (150) and 2 oz granola (120) with 4 turkey sausage links (280)

L –

- Replace 1 oz cheese with 1 oz bacon on burger
- Replace 2 oz cheese with 1 oz bacon on burger (70) and 1 chocolate chip cookie (125)
- Replace 3 oz cheese with 2 oz bacon on burger (140) and additional 8 oz ginger ale (80)
- Replace ranch dressing packet (140) with Italian dressing packet (110)
- Replace 2 ranch dressing packets (280) with 2 Italian dressing packets (220)

3S- Replace mozzarella sticks (400) and marinara sauce (50) with Tastykake © Lemon Pie (490)
D –

- Replace chicken alfredo (320) with 5 BBQ chicken wings (325)
- Replace chicken alfredo (560) with 6 BBQ chicken wings (395) and ½ cup French fries (190) offered with 1 packet ketchup
- Replace chicken alfredo (735) with 6 BBQ chicken wings (395) and 1 cup French fries (380) offered with 2 packets ketchup
- Replace Cookies & Cream ice cream (250) with Tastykake © Koffeekake Junior (280)
- Replace Cookies & Cream ice cream (250) and chocolate syrup (100) with Tastykake© Koffeekake cupcakes (crème filled) (360)
- Replace breadstick (110) with additional ¼ French fries (90)
- Replace 2 breadsticks (220) with additional ½ cup French fries (180)

Thurs:
10S –

- replace 2 oz cubed cheddar cheese (220) with 1.5 oz trail mix.
- Replace 3 oz cubed cheddar cheese (330) with Sweet Street© Lemonberry Jazz Bar (340)

L-

- Replace mac and cheese (230) with hot dog on bun (290) offered with 1 packet ketchup and 1 packet mustard
- Replace mac and cheese (340) with hot dog on bun (290) offered with 1 packet ketchup and 1 packet mustard
- Replace mac and cheese (460) with hot dog on bun (290) offered with 1 packet ketchup and 1 packet mustard and baked lays (140)
- Replace mac and cheese (575) with hot dog on bun (290) offered with 1 packet ketchup and 1 packet mustard, baked lays (140) and extra sugar cookie (125)

D –

Malnutrition Pathway Menu Modifications

- Replace 0.5 oz shredded cheddar (60) with 1 oz extra beef (60)
- Replace 1 sour cream packet with salsa (30)
- Replace 2 sour cream packets with salsa (30) and 2 oz additional avocado (90)
- Replace milkshake (365) with Tastykake © Chocolate Cups (340)
- 5800 and 6000 kcal Menus: Replace 2 oz shredded cheddar (220) and milkshake (365) with 2 packets Tastykake© Dreamies (580)

Fri:
B - No eggs/cheese
- Replace 2 oz scrambled eggs (90) with 2 oz grits (100)
- Replace 3 oz scrambled eggs (135) with 3 oz grits (150)
- Replace 3 oz scrambled eggs (135) with 1 oz cheese (75) with 3 oz grits (150) and 1 turkey sausage link (70)
- Replace 3 oz scrambled eggs (135) with 2 oz cheese (150) with 3 oz grits (150) and 2 turkey sausage links (140); 3 sausage links for 4000-4600 MP
- Replace 4 oz scrambled eggs (180) with 2 oz cheese (150) with 5 oz grits (250). Send 3 turkey sausage (210)
- Replace 6 oz scrambled eggs (270) with 3 oz cheese (225) with 6 oz grits (300). Send 3 turkey sausage (210)
- Replace 6 oz scrambled eggs (270) with 4 oz cheese (300) with 6 oz grits (300). Send 4 turkey sausage (280)

10S – replace chocolate pudding with peanut butter packet (125)
L –
- Replace ½ grilled cheese (250) with ham sandwich, 2 oz, on white bread (230), offer with lettuce, tomato and mustard
- Replace grilled cheese (500) with chicken salad, 4 oz, sandwich on white bread (505)
- Replace ranch dressing (140) with Italian dressing (110)

3S
- Replace Hershey Incredible Cone (300) with Tastykake© Cherry pie (290)
- 5400-6000 kcal Menus: Replace Hershey Dulce de Leche Ice Cream Cup (270) and chocolate syrup (100) with Tastykake© Chocolate Cupcakes (400)

D –
No pizza
- Replace ½ pepperoni pizza (280) with hummus and veggie wrap (300)
- Replace ¾ pepperoni pizza (420) with hummus and veggie wrap made with additional 2 oz hummus (420)
- Replace pepperoni pizza with meatball sandwich on 6” hoagie roll with 3 meatballs and ½ cup sauce (630). No cheese on sandwich.
- 5800 and 6000 kcal menus: No breadstick

Sat:
10S –
- Replace strawberry Greek yogurt (120) with Nature Valley chocolate chip granola bar (100)
- Replace 2 packets cream cheese (200) with 2 packets peanut butter (250)

Malnutrition Pathway Menu Modifications

- 6000 kcal Menu: Note to Nursing, mix granola with part of milk

L –

- NO swiss cheese on sandwich. Send hard pretzels (110)
- Replace Snickers or Twix Ice Cream bar (250) with Sweet Street Pecan Chocolate Chunk bar (300)

3S –

- Replace 1 oz cheese sauce (70) with extra 1 oz tortilla chips (70)
- Replace 2 oz cheese sauce with extra 1oz tortilla chips and extra 4 oz apple juice (60)

D-

- For 2000 and over kcal menus. Replace brownie cupcake/vanilla ice cream/chocolate sauce with Tastykake© Chocolate frosted mini donuts

Sun:

No egg omelet with cheese.

- 1600-2600 MP: Replace 2 oz omelet with 0.5 oz cheese (130) and toast (140) with butter (70) with 3 french toast sticks (225) and syrup (110)
- 2800-3000 MP: Replace 2 oz omelet with 1 oz cheese (165) and toast (140) with butter (70) with 4 french toast sticks (300) and syrup (110)
- 3200 MP: Replace 4 oz omelet with 1 oz cheese (255) and toast (140) with butter (70) with 4 french toast sticks (300) and syrup (110)
- 3400-3800 MP: Replace 4 oz omelet with 2 oz cheese (330) and toast (140), butter (70) with 2 syrups (220) and 5 french toast sticks (375)
- 4000-4200 MP: Replace 4 oz omelet with 2 oz cheese (330) and toast (140), butter (70) and jelly (100) with 2 syrups (220) and 6 french toast sticks (450)
- 4400-4800 MP: Replace 4 oz omelet with 2 oz cheese (330) and toast (210), butter (105) and jelly (150) with 2 syrups (220) and 6 french toast sticks (450), 1 oz bacon (70)
- 5000 MP: Replace 4 oz omelet with 3 oz cheese (405) and toast (210), butter (105) and jelly (150) with 2 syrups (220) and 6 french toast sticks (450), 1 oz bacon (70)

L –

- No 1 oz Swiss cheese on sandwich. Send cherry jello (100)
- No 2 oz Swiss cheese on sandwich. Send brownie cupcake (185)
- 5200 kcal Menu: replace pretzel and 1 oz cheese sauce (250) with Stacy's Pita Chips (200)
- 5400-6000 kcal Menus: replace soft pretzel and 2 oz cheese sauce (320) with Stacy's Pita Chips (200) and 2oz Hummus (150)

3S-

- 5200 and 5400 kcal menus: Replace yogurt (150) with extra ½ Sweet Street© Chocolate Chunk Bar (150)
- 5600-6000 kcal menus: Replace milkshake with 2 Tastykake© Chocolate Juniors (660)

D-

- Replace ice cream sandwich (300) with Tastykake© Dreamies (290)

Malnutrition Pathway Menu Modifications

3. **<u>Egg</u>**

Mon B: replace hardboiled egg (75) with 2 pieces bacon (70), replace 2 hardboiled eggs (150) with 4 pieces bacon (140)

Tues B: no breakfast sandwich.

- Replace breakfast sandwich (165) with 1 blueberry pancake (110) and ½ packet syrup (55)
- Replace breakfast sandwich (240) with 1 blueberry pancake (110) and syrup (110) and pat butter (35)
- Replace breakfast sandwich (315) with 2 blueberry pancakes (220), syrup (110) and butter (35)
- Replace breakfast sandwich (415) with 3 blueberry pancakes (330), syrup (110) and butter (35)
- Replace breakfast sandwich (495) with 3 blueberry pancakes (330), 2 syrup (110) and 2 butter (70)

Friday B:

- Replace 2 oz scrambled eggs (90) with 2 oz grits (100)
- Replace 3 oz scrambled eggs (135) with 3 oz grits (150)
- Replace 3 oz scrambled eggs/1 oz cheese (210) with 3 oz cheese grits using 1 oz American cheese (225)
- Replace 3 oz scrambled eggs/2 oz cheese (285) with 3 oz cheese grits using 2 oz American cheese (300)
- Replace 4 oz scrambled eggs/2 oz cheese (330) with 3 oz cheese grits using 2 oz American cheese (300)
- Replace 6 oz scrambled eggs/3 oz cheese (495) with 3 oz cheese grits using 2 oz American cheese (300). Replace turkey sausage (140) with 2 sausage patties (360)
- Replace 6 oz scrambled eggs/4 oz cheese (570) with 4 oz cheese grits using 3 oz American cheese (360). Replace turkey sausage (140) with 2 sausage patties (360)

Saturday:
L-

- replace diced hard-boiled (75) egg with ¾ oz shredded cheddar (80)
- replace 2 diced hard-boiled eggs (150) with 1.5 oz shredded cheddar (160)

Sunday B:

- Replace 2 oz egg omelet (90) with light strawberry yogurt
- Replace 2 oz omelet with 0.5 oz cheese (130) with regular strawberry yogurt (120)
- Replace 2 oz omelet with 1 oz cheese (165) with regular strawberry yogurt (120) and 1 oz granola (60)
- Replace 4 oz omelet with 1 oz cheese (255) with regular strawberry yogurt (120) and 1 oz granola (60) and banana (110)
- Replace 4 oz omelet with 2 oz cheese (330) with regular strawberry yogurt (120) and 2 oz granola (120) and banana (110)

Malnutrition Pathway Menu Modifications

- Replace 4 oz omelet with 3 oz cheese (405) with regular strawberry yogurt (150) and 3 oz granola (180) and banana (110)
- Replace 4oz omelet with 4 oz cheese (475) with regular strawberry yogurt (150) and 4 oz granola (240) and banana (110)

Malnutrition Pathway Menu Modifications

4. **<u>Fish</u>**
   No fish.
   Mon L: replace tilapia with 1 piece fried chicken (~195)
   Sat L: replace tuna salad with chicken salad

Malnutrition Pathway Menu Modifications

5. **<u>Gluten-Free</u>**

Monday:

- B – replace granola with banana (110)
- 10S –
  - Replace corn muffin (220) and peaches (50) with GF English muffin (210) and peanut butter (125)
  - Replace corn muffin (220) with butter (35) with ½ GF bagel (145) and cream cheese (100)
  - Replace bagel with GF bagel (290)
- L – Newman's Own Caesar dressing (150)
- 3S – replace Tastykake© Koffee Kake (360) with 8 GF Glutino chocolate covered vanilla wafers (320)
- D – Newman's Own Ranch dressing (220)
  - Replace caramel apple bar (280) with milkshake made with 8 oz whole milk, ½ cup chocolate ice cream, 1 T chocolate syrup (320)
  - Replace 2 Caramel Apple bars (560) with chocolate milkshake made with 10 oz whole milk, 1.5 T chocolate syrup, 3/4 cup chocolate ice cream (545)

Tuesday:

- B – GF english muffin for breakfast sandwich.
- 10S –
  - Replace 2 oz granola (120) with ¾ cup GF Honey Nut Chex (120)
  - Replace 3 oz granola (180) with 1 cup GF Honey Nut Chex (160)
  - Replace 4 oz granola (240) with 1.5 cups GF Honey Nut Chex (240)
  - Replace 2 sugar cookies (250) with banana (110) and peanut butter (125)
- L – NO meatballs. GF fusilli pasta.
  - 1400-1600 MP: no meatball. Replace marinara sauce with 4 oz meat sauce (150)
  - 1800-3200 MP: no 2 meatballs. Send 10 oz GF pasta (500) with 4 oz meat sauce (150)
  - 3400-4600 MP: no 2 meatballs. Send 10 oz GF pasta (500) with 6 oz meat sauce (225), GF bread (70) with butter (35)
  - 4800 MP: no 3 meatballs. Send 10 oz GF pasta (500) with 8 oz meat sauce (300), GF bread (70) with butter (35)
  - 5000-5400 MP: no 3 meatballs. Send 12 oz GF pasta (600) with 8 oz meat sauce (300), GF bread (70) with butter (35)
  - 5600-6000 MP: no 3 meatballs. Send 12 oz GF pasta (600) with 8 oz meat sauce (300), 2x GF bread (140) with 3 pats butter (105)
- 3S – GF hard pretzels (120). Send 2 bags (240) to replace soft pretzel.
- D –
- replace 1 chocolate chip cookie (125) with extra ½ cup mashed potatoes (130)
- replace 2 chocolate chip cookies (250) with GF chocolate chip cookie (210)
- replace 3 chocolate chip cookies (375) with 2 GF chocolate chip cookies (420)
- replace 3 oz gravy with 1 pat butter (35) melted on mashed potatoes
- replace 6 oz gravy with 2 pats butter melted on mashed potatoes (70).

Malnutrition Pathway Menu Modifications

Wednesday:

- B –
    - GF bagel (310)
    - Replace 2 oz granola (120) with 2 turkey sausage links (140)
    - Replace 3 oz granola (180) with 3 turkey sausage links (210)
    - Replace 4 oz granola (240) with 3 turkey sausage links (210)
- 10S –
    - replace fruit loops with ½ cup GF Honey Nut Chex (80)
    - replace blueberry muffin (180) and butter (35) with 8 GF glutino crackers (140) and 1 oz cubed cheddar cheese (110)
    - Replace 2 blueberry muffins (360) and 2 pats butter (70) with 1 ½ cups GF Honey Nut Corn Chex (240) and almond and coconut kind bar (GF) (210)
- L –
    - GF burger roll.
    - Replace sun chips with Doritos, same portions
    - Newman's Own Ranch dressing, replace same number of packets (220 per packet)
    - 6000 kcal menu: replace lemonberry jazz bar (340) with 8 GF Glutino chocolate covered vanilla wafers (320)
- 3S
    - GF bread for PBJ
    - Replace mozzarella sticks (400) and marinara sauce (50) with 2 Udi's GF Chocolate Chip cookies (420)
- D – using GF elbow pasta (same portions)
    - No Alfredo
        - Replace 2 oz alfredo (50) with 2 pats melted butter on pasta
        - Replace 4 oz alfredo (100) with 3 pats melted butter on pasta
    - Replace cookies & cream ice cream (250) with 1 cup (8 oz) chocolate ice cream (240)
    - 4800-5000 MP: No breadstick (110). Send 10 oz pasta.
    - 5600-6000 MP: No breadsticks (220). Send 10 oz pasta and add 1 oz melted cheese on pasta (70)

Thursday:

- B –
  No French toast.
    - Replace 3 French toast (195) and syrup (110) with 2 GF Van's Ancient Grains waffles (220) and syrup (110)
    - Replace 4 French toast (260) and syrup (110) with 2 GF Van's Ancient Grains waffles (220), syrup (110) and 1 pat butter (35)
    - Replace 4 French toast (260), syrup (110) and butter (35) with 2 GF Van's Ancient Grains waffles (220), syrup (110) and 2 pats butter (70)
    - Replace 5 French toast (325), syrup (110) and butter (35) with 3 GF Van's Ancient Grains waffles (330), syrup (110) and butter (35)
    - Replace 6 French toast (390) and 2 syrup (220) with 3 GF Van's Ancient Grains waffles (330), 2 syrup (220) and 2 butter (70)

Malnutrition Pathway Menu Modifications

    - Replace 8 French toast (520) and 2 syrup (220) with 4 GF Van's Ancient Grains waffles (440), 2 syrup (220) and 3 butter (105)
- L –
    - GF mac & cheese
        - Replace 4 oz mac and cheese with 5 oz GF Amy's Mac and Cheese
        - Replace 6 oz mac and cheese with 8 oz GF Amy's Mac and Cheese
        - Replace 8 oz mac and cheese with 10 oz GF Amy's Mac and Cheese
        - Replace 10 oz mac and cheese with 13 oz GF Amy's Mac and Cheese
    - Replace sugar cookie (125) with 3 GF chocolate covered vanilla wafers (120)
    - replace 2 sugar cookies (250) with GF chocolate chip cookie (210)
    - Replace 3 sugar cookies (375) with 2 GF chocolate chip cookies (420)
- 3S –
    - Replace peanut butter crackers (130) with 8 Glutino crackers (140)
    - Replace Ritz crackers, 5 (80) with 5 Glutino crackers (90)
    - Replace Ritz crackers, 7 (110) with 6 Glutino crackers (110)
    - Replace Ritz crackers, 10 (160) with 8 Glutino crackers (140)
    - Replace Stacy's pita chips (200) with 12 Glutino crackers (215)
- D –
    - Corn tortilla for fajitas
    - Replace brownie cupcake (185) with milkshake made with 4 oz chocolate ice cream and ½ cup whole milk (215)

Friday:

- B –
    - GF bread for toast
    - replace biscuit (220) with GF English muffin (210)
- 10S –
    - replace NV granola bar (90) with 2 Glutino chocolate covered vanilla wafers (80)
    - replace 2 NV granola bars (180) with 4 Glutino chocolate covered vanilla wafers (160)
    - replace 3 NV granola bars (270) with Sesame and Peanut Kind bar (GF) (240)
- L –
    - GF bread for grilled cheese.
    - Replace tomato soup with Glutino pretzels (120).
    - Replace goldfish with Glutino pretzels (120).
        - 4200-5800 MP will have 2 packs Glutino pretzels (240)
        - 6000 MP will have 2 packs Glutino pretzels (240) and peanut butter (125)
    - Newman's Own Ranch dressing (220)
- 3S –
    - Replace Nilla wafers (70 and 105) with extra peanut butter packet (125)
    - Replace Incredible Cone with 2 bags Cheetos (300)
- D –
    - Replace ½, ¾ and whole pizza with whole GF Cheese Pizza JD's Best (370)
    - No oatmeal raisin cookie bar (200)
    - No Butter Kreme Snack (400)
    - 1800-2400 MP: send ¾ cup orange sherbet (180)

        - 2600-3200 MP: send milkshake made with 1 cup orange sherbet , 4 oz orange juice, 4 oz whole milk, ½ cup vanilla ice cream (400)
        - 3400-6000 MP: send milkshake made with 1 cup orange sherbet, 6 oz orange juice, 6 oz whole milk, 1 cup vanilla ice cream (610)
    - 5800 and 6000 MP: Replace breadstick (110) with Cheetos (150)

Saturday:

- B –
    - Replace 1 pancake with ¼ cup dry mix Carbon's Golden Malted GF pancake mix
    - Replace 2 pancakes with ½ cup dry mix Carbon's Golden Malted GF pancake mix
    - Replace 3 pancakes with ¾ cup dry mix Carbon's Golden Malted GF pancake mix
    - Replace 4 pancakes with 1 cup dry mix Carbon's Golden Malted GF pancake mix
    - 6000 MP: Replace cinnamon bun (160) with additional (fifth) pancake made with ¼ cup dry mix Carbon's Golden Malted GF pancake mix
- 10S
    - GF bagel
    - 6000 MP: Replace 4 oz granola (240) with additional packet cream cheese (100) and 2 oz raisins (130)
- L –
    - GF bread for sandwich
    - GF Newman's Own Italian dressing,
    - Replace Snickers or Twix ice cream bar (250) with 1 cup (8 oz) vanilla ice cream (240)
- D –
    - No lomein.
        - Replace LoMein (370) with 3 oz oven baked chicken (150), ½ cup mashed potatoes (130), ½ cup corn (100)
        - Replace LoMein (420) with 3 oz oven baked chicken (150), ½ cup mashed potatoes (130), ½ cup corn (100) with 1 pat butter (35)
        - Replace LoMein (490) with 3 oz oven baked chicken (150), ¾ cup mashed potatoes (195), ½ cup corn (100) with 1 pat butter (35)
    - No spring rolls.
        - Replace 1 spring roll (200) with additional ½ cup mashed potatoes (130)
        - Replace 2 spring rolls (400) with additional 1 cup mashed potatoes (260) and additional 3 pats butter (105)
        - Replace 3 spring rolls (600) with additional 1 cup mashed potatoes (260), additional 1 cup corn (200) and additional 4 pats butter (140)
    - Replace brownie cupcake (185) with milkshake made with 4 oz vanilla ice cream and ½ cup whole milk (215)
    - Brownie cupcake/vanilla ice cream/syrup (420) with milkshake made with 1 cup strawberry ice cream and 8 oz whole milk (430)

Sunday:

- B – GF bread for toast

Malnutrition Pathway Menu Modifications

- 10S
  - replace 1 box cheerios (75) with ½ cup GF Honey Nut Chex (80)
  - replace 2 boxes cheerios (150) with 1 cup GF Honey Nut Chex (160)
- L –
  - GF bread for sandwich
  - Replace pretzels (110) with GF hard pretzels (120)
  - Replace soft pretzel (180) with 2 GF hard pretzels (240)
- 3S –
  - Replace 2 peanut butter crackers (65) with light vanilla yogurt (90)
  - Replace 4 peanut butter crackers (130) with regular vanilla yogurt (150)
  - Replace Sweet Street Pecan Chocolate Chunk Bar (300) with microwave popcorn, 2 cups (340)
- D –
  - No chicken tenders
    - Replace 2 chicken tenders (200) with 2 broccoli and cheddar potato skins (280)
    - Replace 3 chicken tenders (300) with 2 broccoli and cheddar potato skins (280)
    - Replace 4 chicken tenders (400) with 3 broccoli and cheddar potato skins (420)
    - Replace 5 or 6 chicken tenders (500 or 600) with 4 broccoli and cheddar potato skins (560)
  - Replace ice cream sandwich (300) with vanilla milkshake made with ½ cup vanilla ice cream and 8 oz whole milk (270)

Malnutrition Pathway Menu Modifications

6. <u>Halal</u>

Mon :
L – replace tilapia with 3 Halal fish nuggets (165)
D- replace grilled chicken (all portions) with same portion of Halal chicken

Tues :
L:
- replace 1 meatball (100) with 1 oz mozzarella cheese melted onto pasta (70)
- replace 2 meatballs (200) with 1 oz mozzarella cheese melted onto pasta (70) and breadstick (110)
- replace 3 meatballs (300) with 1 oz mozzarella cheese melted onto pasta (70) and breadstick (110), replace Rice Krispie Treat with Hershey Incredible Cone (300)
- replace 5 meatballs (500) with 3 oz mozzarella cheese melted onto pasta (210) and replace Rice Krispie Treat with Hershey Incredible Cone (300)

D:
- replace roast turkey (185), ½ cup mashed potatoes (130), broccoli (20) with 3 broccoli and cheddar potato skins (420)
- replace roast turkey and gravy (225), ½ cup mashed potatoes (130), broccoli with butter (35) with 3 broccoli and cheddar potato skins (420)
- replace roast turkey and gravy (225), ¾ cup mashed potatoes (195), broccoli with butter (35) with 3 broccoli and cheddar potato skins (420)
- replace roast turkey and gravy (225), 1 cup mashed potatoes (260), broccoli with butter (35) with 4 broccoli and cheddar potato skins (560)
- replace roast turkey and gravy (290), 1 cup mashed potatoes (260), broccoli with butter (35) with 4 broccoli and cheddar potato skins (560)
- replace roast turkey and gravy (450), 1 cup mashed potatoes (260), broccoli with butter (35) with 5 broccoli and cheddar potato skins (700)

Wed:
L: replace burger patty with Halal hamburger patty
D: replace chicken with Halal chicken in pasta alfredo

Thurs:
B:
- replace 1 link turkey sausage (70) with 2 oz scrambled eggs (90)
- replace 2 links turkey sausage (140) with 2 oz scrambled eggs with 1 oz American cheese (165)
- replace sausage patty (180) with 2 oz scrambled eggs with 1 oz American cheese (165)
- replace 2 sausage patties (360) with 4 oz scrambled eggs with 2 oz American cheese (330)
- replace 3 sausage patties (540) with 4 oz scrambled eggs with 3 oz American cheese (390), extra ½ cup pineapple (70), 3 pats butter (105)

D - replace beef (120) with 4 oz Halal chicken (145) in fajita

Malnutrition Pathway Menu Modifications

Fri:
B:
- No turkey sausage
- 4000-4600 MP: Send 4 oz scrambled eggs with 2 oz cheese (320)
- 4800 MP: Send 4 oz scrambled eggs with 3 oz cheese (390)
- 5000 MP: Send 6 oz scrambled eggs with 4 oz cheese (550)
- 5200-6000 MP: Send additional biscuit (220) and additional pat butter (35)

D - replace pepperoni pizza (560) with Halal beef pepperoni pizza

Sat:
B:
- replace 2 oz turkey bacon (70) with 2 oz scrambled eggs (90)
- replace 3 oz turkey bacon (105) with 2 oz scrambled eggs (90)
- replace 4 oz turkey bacon (140) with 2 oz scrambled eggs with 1 oz American cheese (165)

D - replace chicken in LoMein with 4 oz Halal chicken (144)

Sun:
B:
- Replace sausage patty (180) with vanilla yogurt (150)
- Replace 2 sausage patties (360) with vanilla yogurt (150), 2 oz raisins (130), 1 oz granola (60)
- Replace 3 sausage patties (540) with vanilla yogurt (150), 2 oz raisins (130), Tastykake© Koffee Kake Junior (280)

L:
- replace turkey sandwich (360) with hummus and veggie wrap with additional 1 oz of hummus added to wrap (375)
- replace turkey sandwich (470) with hummus and veggie wrap with additional 2 oz of hummus added to wrap (450)
- replace turkey sandwich (580) with hummus and veggie wrap with additional 2 oz of hummus added to wrap (450), replace broccoli and butter (55) with 1 cup cooked peas (100) and 1 pat butter (35)

D:
- replace 2 chicken tenders (200) with 1 Halal crispy chicken strip offered with 2 packets ketchup
- replace 3 chicken tenders (300) with 1.5 Halal crispy chicken strip offered with 2 packets ketchup
- replace 4 chicken tenders (400) with 2 Halal crispy chicken strip offered with 3 packets ketchup
- replace 5 chicken tenders (500) with 2.5 Halal crispy chicken strip offered with 4 packets ketchup
- replace 6 chicken tenders (600) with 3 Halal crispy chicken strip offered with 4 packets ketchup

Malnutrition Pathway Menu Modifications

7. **Kosher**

Do not serve dairy and meat in same meal, including butter, sour cream, etc. (1 pat butter = 2 Smart Balance)

Mon:
L: no tilapia or pineapple. Send K roasted turkey meal (290)
D:
- Replace milk with K grape juice
- No grilled chicken breast, cantaloupe or carrots. Send K roasted chicken meal with schliskes, carrots and beans (290)
- Replace each 1 pat butter (35) with 2 smart balance (30)
- 4600 kcal+, replace ranch with Italian dressing (110)
- 5800 kcal MP: Replace 1 oz sour cream (50) with additional ½ cup raw carrots (35)
- 6000 kcal MP: Replace baked potato/butter/sour cream/cheese with 5 oz tater tots (450)

Tues:
L:
- replace 1 meatball (100) with 1 oz mozzarella cheese melted onto pasta (70)
- replace 2 meatballs (200) with 1 oz mozzarella cheese melted onto pasta (70) and breadstick (110)
- replace 3 meatballs (300) with 1 oz mozzarella cheese melted onto pasta (70) and breadstick (110), replace Rice Krispie Treat with Hershey Incredible Cone (300)
- replace 5 meatballs (500) with 3 oz mozzarella cheese melted onto pasta (210) and replace Rice Krispie Treat with Hershey Incredible Cone (300)

D:
- replace 3 oz roast turkey (185) with grilled salmon (~200)
- replace 3 oz roast turkey with 3 oz gravy (225) with grilled salmon (~200)
- replace 4 oz roast turkey with 4 oz gravy (290) with 1.5 servings of grilled salmon (~300)
- replace 6 oz roast turkey with 6 oz gravy (450) with K filet of salmon meal with mixed veggies (417)

Wed:
B- send K bagel (290)
10S – send K blueberry muffin (260)
L:
- replace half hamburger (190) with half black bean veggie burger (140)
- replace hamburger (375) with black bean veggie burger (280) with 1 oz of American cheese (75)
- replace cheeseburger (450) with black bean veggie burger with 2 oz of American cheese (430)
- replace cheeseburger (525) with black bean veggie burger with 2 oz of American cheese (430) and 2 oz avocado on burger (90)

Malnutrition Pathway Menu Modifications

- replace cheeseburger (600) with black bean veggie burger with 3 oz of American cheese (500) and 2 oz avocado on burger (90)

D:
- No chicken in alfredo
- 1200-1600 kcal MP: Add one breadstick (110)
- 1800-4000 kcal MP: Replace cookies & cream ice cream (250) with Tastykake© Butter Kreme snack (3 small cupcakes) (400)
- 4200-5200 kcal MP: Replace cookies & cream ice cream (250) with Tastykake© chocolate frosted mini donuts (440)
- 5400-6000 kcal MP: Replace cookies & cream ice cream (250)/chocolate sauce (100) with Tastykake© Lemon Pie (490)

Thurs:

B:
- replace 1 link turkey sausage (70) with 2 oz scrambled eggs (90)
- replace 2 links turkey sausage (140) with 2 oz scrambled eggs with 1 oz American cheese (165)
- replace sausage patty (180) with 2 oz scrambled eggs with 1 oz American cheese (165)
- replace 2 sausage patties (360) with 4 oz scrambled eggs with 2 oz American cheese (330)
- replace 3 sausage patties (540) with 5 oz scrambled eggs with 3 oz American cheese (435) and 3 pats butter (105)

D:
- replace fajitas with K rib eye meal with carrots and potato kugel (420)
-1200-1400 kcal MP: send water in place of beverage
-1600 kcal MP: replace milk with K apple juice
-1800-2000 kcal MP: replace milk with K apple juice, no cheese
- 2200-3200 kcal MP: replace milk with K apple juice. No cheese. Replace milkshake (365) with Tastykake© Koffee Kake (360)
-3400-3600 kcal MP: Replace milk with K apple juice. No cheese or sour cream. Replace milkshake (365) with Tastykake© Koffee Kake (360)
- 5000-6000 kcal MP: replace milk with K apple juice. No sour cream, cheese or avocado. Replace butter with 2 smart balance (35) Replace milkshake with Tastykake© Apple Pie (470)

Fri:

B –
- 4000-4600 kcal MP: replace 1 link turkey sausage (70) with 2 oz scrambled eggs (90)
- 4800-5000 kcal MP: replace 2 links turkey sausage (140) with 2 oz scrambled eggs with 1 oz American cheese (165)
- 5200-6000 kcal MP: replace 4 links turkey sausage (280) with 3 oz scrambled eggs with 2 oz American cheese (275)

D:
- Replace ½ pizza with 10 oz (0.75 boxes) K stuffed shells (290)
- Replace ¾ pizza with 14 oz (1.25 boxes) K stuffed shells (420)
- Replace pizza with 18 oz (1.5 boxes) K stuffed shells (525)

Malnutrition Pathway Menu Modifications

Sat:
B:
- replace 2 oz turkey bacon (70) with 2 oz scrambled eggs (90)
- replace 3 oz turkey bacon (105) with 2 oz scrambled eggs (90)
- replace 4 oz turkey bacon (140) with 2 oz scrambled eggs with 1 oz American cheese (165)

10S – K bagel (290)
L - Use K tuna for sandwich
D:
- Replace 8oz milk with 8 oz K grape juice (135)
- Replace 12 oz milk with 12 oz K grape juice (200)
- Replace chicken LoMein (370 and 420) with K Salisbury steak meal with mashed potatoes and carrots (430)
- Replace brownie cupcake/ice cream/chocolate sauce (425) with K brownie (350) and ½ cup pineapple (70)

Sun:
B:
- Replace sausage patty (180) with vanilla yogurt (150)
- Replace 2 sausage patties (360) with vanilla yogurt (150), 2 oz raisins (130), 1 oz granola (60)
- Replace 3 sausage patties (540) with vanilla yogurt (150), 2 oz raisins (130), Tastykake© Koffee Kake Junior (280)

L:
- 1200-2400 kcal MP: replace turkey sandwich (360) with K meatballs and spaghetti (430)
- 2600-3200 kcal MP: replace turkey sandwich (470) with K meatballs and spaghetti (430)
- 3400-6000 kcal MP: replace turkey sandwich (580) with K meatballs and spaghetti (430) and K chocolate chip cookies, 4 oz (140)
- 3400-6000 kcal MP: replace 1 pat butter with 2 smart balance (30)

-5200-6000 kcal MP: replace cheese sauce (70 or 140) with peanut butter for apple (125)
D:
- replace whole milk with OJ
- 1800-6000 kcal MP: replace 1 pat butter with 2 smart balance
- 2000-6000 kcal MP: replace ice cream sandwich (300) with Tastykake© Koffeekake Juniors (280)
- replace 2 chicken tenders (200) with K beef brisket meal with grilled potatoes, peas and carrots (390), no french fries
- replace 3 chicken tenders (300) with K beef brisket with grilled potatoes, peas and carrots (390), reduce french fry portion by ¼ cup
- replace 4 chicken tenders (400) with K beef brisket with grilled potatoes, peas and carrots (390)
- replace 5 chicken tenders (500) with K beef brisket with grilled potatoes, peas and carrots (390), increase french fry portion by ¼ cup (90)

Malnutrition Pathway Menu Modifications

8. <u>NO milk/meat together in meal (non-kosher meats)</u>

Mon:
D:
- Replace milk with grape juice.

Tues:
L:
- replace 1 meatball (100) with 1 oz mozzarella cheese melted onto pasta (70)
- replace 2 meatballs (200) with 1 oz mozzarella cheese melted onto pasta (70) and breadstick (110)
- replace 3 meatballs (300) with 1 oz mozzarella cheese melted onto pasta (70) and breadstick (110), replace Rice Krispie Treat with Hershey Incredible Cone (300)
- replace 5 meatballs (500) with 3 oz mozzarella cheese melted onto pasta (210) and replace Rice Krispie Treat with Hershey Incredible Cone (300)

D:
-replace milk with grape juice
- replace 1 pat butter with 2 smart balance

Wed:
L:
-Replace 1 oz of cheese with 2 oz avocado on burger (90)
-Replace 2 oz of cheese with rice krispie treat (150)
-3600-6000 kcal MP: Replace ranch dressing packet(s) (140 each) with Italian dressing packet(s) (110 each)
-5800 kcal MP: Replace 3oz of cheese (210) and ice cream (140) with Tastykake© Koffee Kake cupcakes (3 small cupcakes) (360)
-6000 kcal MP: No cheese on burger (210). Replace ginger ale with 12 oz grape juice (255). Add 2 oz avocado on burger (90).

D:
- <u>No chicken</u> in alfredo
- 1200-1600 kcal MP: Add one breadstick (110)
- 1800-4000 kcal MP: Replace cookies & cream ice cream (250) with Tastykake© Butter Kreme snack (3 small cupcakes) (400)
- 4200-5200 kcal MP: Replace cookies & cream ice cream (250) with Tastykake© chocolate frosted mini donuts (440)
- 5400-6000 kcal MP: Replace cookies & cream ice cream (250)/chocolate sauce (100) with Tastykake© Lemon Pie (490)

Thurs:
B:
- replace 1 link turkey sausage (70) with 2 oz scrambled eggs (90)
- replace 2 links turkey sausage (140) with 2 oz scrambled eggs with 1 oz American cheese (165)
- replace sausage patty (180) with 2 oz scrambled eggs with 1 oz American cheese (165)
- replace 2 sausage patties (360) with 4 oz scrambled eggs with 2 oz American cheese (330)

Malnutrition Pathway Menu Modifications

- replace 3 sausage patties (540) with 5 oz scrambled eggs with 3 oz American cheese (435) and 3 pats butter (105)

D:

- Replace beef in fajitas with tofu

Fri:

B:

- 4000-4600 kcal MP: replace 1 link turkey sausage (70) with 2 oz scrambled eggs (90)
- 4800-5000 kcal MP: replace 2 links turkey sausage (140) with 2 oz scrambled eggs with 1 oz American cheese (165)
- 5200-6000 kcal MP: replace 4 links turkey sausage (280) with 3 oz scrambled eggs with 2 oz American cheese (275)

D:

- Replace ½ pepperoni pizza with 1/2 cheese pizza (280)
- Replace ¾ pepperoni pizza with 3/4 cheese pizza (420)
- Replace pepperoni pizza with cheese pizza (560)

Sat:

B:

- replace 2 oz turkey bacon (70) with 2 oz scrambled eggs (90)
- replace 3 oz turkey bacon (105) with 2 oz scrambled eggs (90)
- replace 4 oz turkey bacon (140) with 2 oz scrambled eggs with 1 oz American cheese (165)

D:

- Replace whole milk with grape juice

Sun:

B:

-Replace sausage patty (180) with vanilla yogurt (150)

-Replace 2 sausage patties (360) with vanilla yogurt (150), 2 oz raisins (130), 1 oz granola (60)

-Replace 3 sausage patties (540) with vanilla yogurt (150), 2 oz raisins (130), Tastykake© Koffee Kake Junior (280)

L:

- Replace 1 oz swiss (70) cheese with 2 oz avocado (90)
- Replace 2 oz swiss cheese (140) with 1 packet peanut butter (125)
- Replace 3 oz swiss cheese (210) with 1 packet peanut butter (125) and 2 oz avocado (90)

D:

- replace whole milk with OJ
- replace 1 pat butter with 2 pats smart balance
- replace ice cream sandwich with Sweet Street Pecan Chocolate chunk bar (300)

Malnutrition Pathway Menu Modifications

9. **Nuts**

Monday:

- B-
  - Replace 1 oz granola (60) with 2 slices turkey bacon (70)
  - Replace 2 oz granola (120) with 4 slices turkey bacon (140)
  - Replace 3 oz granola (180) with cinnamon bun (160)
- 10S
  - Replace peanut butter packet (125) with cream cheese (100)
- 3S
  - Replace Tastykake© Koffee Kake (360) with 3 sugar cookies (360)
- D –
  - Replace Caramel Apple bar (280) with Hershey Incredible Cone (300)
  - Replace 2 Caramel Apple bars (560) with chocolate milkshake made with 10 oz whole milk, 1.5 T chocolate syrup, 3/4 cup chocolate ice cream (545)

Tuesday:

- 10S
  - Replace 2 oz granola (120) with 2 oz raisins (130)
  - Replace 3 oz granola (180) with 3 oz raisins (195)
  - Replace 4 oz granola (240) with 3 oz raisins (195) and increase honeydew to 1 cup (60)
- D –
  - Replace chocolate chip cookie (125) with mango fruit bar (100)
  - Replace 2 chocolate chip cookies (250) with Good Humor chocolate éclair (220)
  - Replace 3 chocolate chip cookies with (375) with Good Humor chocolate éclair (220) and send additional ½ cup mashed potatoes

Wednesday:

- B-
  - Replace 2 oz granola (120) with 2 oz raisins (130)
  - Replace 3 oz granola (180) with 3 oz raisins (195)
  - Replace 4 oz granola (240) with 3 oz raisins (195) and increase watermelon to 1 cup (100)
- L-
  - 6000 kcal MP: Replace Sweet Street© Lemonberry Jazz bar (340) with 1 cup chocolate ice cream (280) and 1T chocolate syrup (50)
- 3S
  - Replace ½ PBJ with 2 oz hummus (120) and 4 Ritz crackers (65)
  - Replace whole PBJ (380) with 4 mozzarella sticks (380) served with 2 packets ketchup

Thursday:

- 10S-
  - Replace 1 oz trail mix (160) with 10 ritz crackers (160)

Malnutrition Pathway Menu Modifications

  - Replace 2 oz trail mix (320) with sun chips (210) and additional 1 oz cubed cheddar (110)
- L –
  - Replace peanut butter packet with extra sugar cookie (125)
- D
  - Replace brownie cupcake (185) with vanilla ice cream cup (140) and 1 T chocolate syrup (40)

Friday:

- 10S –
  - Replace 1 NV granola bar (90) with 5 ritz crackers (80)
  - Replace 2 NV granola bars (180) with blueberry muffin (180)
  - Replace 3 NV granola bars (270) with additional chocolate pudding (130), English muffin (120) and 1 pat butter (35)
- L
  - 4600-5800 kcal MP: Send 2 packs goldfish with lunch (260) (this replaces peanut butter at 3pm snack)
- 3S – no peanut butter packet (125)
  - For MP 4000, 4200 kcal MP: Send 14 Nilla wafers
  - For 4600-5800: Send 2 packs goldfish with lunch (260)
  - 6000 kcal MP: Send additional 2 T chocolate syrup (100)
- D –
  - 2600-3200 kcal MP: Replace Tastykake© Oatmeal Raisin Bar with Hershey Cookies and Cream ice cream (250)
  - 3400-6000 kcal MP: Replace Tastykake© Butter Kreme Snack (cupcakes) (400) with milkshake made with 1 cup orange sherbert , 4 oz orange juice, 4 oz whole milk, 1 cup vanilla ice cream (400)

Saturday:

- 10S
  - 6000 kcal MP: Replace 4 oz granola (240) with 2 oz raisins (130) and additional packet cream cheese (100)
- L –
  - Replace Snickers or Twix Ice Cream Bar (250) with ice cream sandwich (300)
- D –
  - 1800-3800 kcal MP: Replace brownie cupcake (185) with spring roll (200)
  - 4000-5200 kcal MP: Replace brownie cupcake (185) with additional spring roll (200)
  - 5400-6000 kcal MP: Replace brownie cupcake (185) with additional (second) vanilla ice cream cup (120) and additional 1T chocolate syrup (50)

Sunday:

- 10S
  - 5800 and 6000 kcal MP: Replace peanut butter packet (125) with 2 oz raisins (130)
- 3S –

Malnutrition Pathway Menu Modifications

- Replace 2 peanut butter crackers (65) with 5 ritz crackers (80)
- Replace 4 peanut butter crackers (130) with 8 ritz crackers (130)
- Replace Sweet Street© Pecan Chocolate Chunk bar (300) with BBQ Lays chips (160) and chocolate pudding (130)

Malnutrition Pathway Menu Modifications

10. <u>Red Meat</u>
No red meat, please. Please replace:
All bacon should be turkey bacon.

Tues L:

- replace 1 meatball (100) with 1 oz mozzarella cheese melted onto pasta (70)
- replace 2 meatballs (200) with 1 oz mozzarella cheese melted onto pasta (70) and breadstick (110)
- replace 3 meatballs (300) with 1 oz mozzarella cheese melted onto pasta (70) and breadstick (110), replace Rice Krispie Treat with Hershey Incredible Cone (300)
- replace 5 meatballs (500) with 2 oz mozzarella cheese melted onto pasta (140), replace Rice Krispie Treat with Hershey 6oz Dulce de Leche ice cream cup (270) with 2 T chocolate syrup (100)

Wed:
L:

- replace half hamburger (190) with half turkey burger (125)
- replace hamburger (375) with turkey burger with 1 oz of American cheese (325)
- replace cheeseburger (450) with turkey burger with 2 oz of American cheese (400)
- replace cheeseburger (525) with turkey burger with 2 oz of American cheese and 2 oz avocado on burger (490)
- replace cheeseburger (600) with turkey burger with 3 oz of American cheese and 2 oz avocado on burger (560)

Thurs:
B -

- Replace 1 sausage patty (180) with 1 turkey sausage link (130)
- Replace 2 sausage patties (360) with 3 turkey sausage links (390)
- Replace 3 sausage patties (540) with 4 turkey sausage links (520)

D – Replace beef fajitas with chicken fajitas

Fri:
D – Replace pepperoni pizza with cheese pizza

Sun:
B -

- Replace 1 sausage patty (180) with 1 turkey sausage link (130)
- Replace 2 sausage patties (360) with 3 turkey sausage links (390)
- Replace 3 sausage patties (540) with 4 turkey sausage links (520)

Malnutrition Pathway Menu Modifications

11. <u>Vegetarian - Pescetarian Modifications to MP</u>

Please make the following changes:
Mon :
D-

- Replace grilled chicken breast (140) with 4 oz grilled tofu (145)
- Replace 1.5 grilled chicken breasts (210) with 6 oz grilled tofu (210)
- Replace 2 grilled chicken breasts (280) with 8 oz grilled tofu (280)
- Replace 2.5 grilled chicken breasts (350) with 8 oz grilled tofu (180) and 2 packets BBW sauce for tofu (70)

Tues :
L:

- Replace 1 meatball (100) with 1 oz mozzarella cheese melted onto pasta (70)
- Replace 2 meatballs (200) with 1 oz mozzarella cheese melted onto pasta (70) and breadstick (110)
- Replace 3 meatballs (300) with 1 oz mozzarella cheese melted onto pasta (70) and breadstick (110), replace Rice Krispie Treat with Hershey Incredible Cone (300)
- Replace 5 meatballs (500) with 3 oz mozzarella cheese melted onto pasta (210) and replace Rice Krispie Treat with Hershey Incredible Cone (300)

D:

- Replace 3 oz roast turkey (185) with salmon burger (250)
- Replace 3 oz roast turkey and 3 oz gravy (225) with salmon burger (250)
- Replace 4 oz roast turkey and 3 oz gravy (290) with salmon burger (250) and one packet tartar sauce (60)
- Replace 6 oz roast turkey and 6 oz gravy (450) with salmon burger (250) on bun (150) and one packet tartar sauce (60)

Wed:
L:

- Replace half hamburger (190) with half black bean veggie burger (140)
- Replace hamburger (375) with black bean veggie burger (280) with 1 oz of American cheese (75)
- Replace cheeseburger (450) with black bean veggie burger with 2 oz of American cheese (430)
- Replace cheeseburger (525) with black bean veggie burger with 2 oz of American cheese (430) and 2 oz avocado on burger (90)
- Replace cheeseburger (600) with black bean veggie burger with 3 oz American cheese (500) and 2 oz avocado on burger (90)

D:

- Replace chicken with tofu in pasta alfredo

Thurs:
B:

- Replace 1 link turkey sausage (70) with 2 oz scrambled eggs (90)

Malnutrition Pathway Menu Modifications

- Replace 2 links turkey sausage (140) with 2 oz scrambled eggs with 1 oz American cheese (165)
- Replace sausage patty (180) with 2 oz scrambled eggs with 1 oz American cheese (165)
- Replace 2 sausage patties (360) with 4 oz scrambled eggs with 2 oz American cheese (330)
- Replace 3 sausage patties (540) with 5 oz scrambled eggs with 3 oz American cheese (435) and 3 pats butter (105)

D:

- Replace beef (120) with 4 oz tofu (145) in fajita

Fri:

B:

- 4000-4600 kcal MP: replace 1 link turkey sausage (70) with 2 oz scrambled eggs (90)
- 4800-5000 kcal MP: replace 2 links turkey sausage (140) with 2 oz scrambled eggs with 1 oz American cheese (165)
- 5200-6000 kcal MP: replace 4 links turkey sausage (280) with 3 oz scrambled eggs with 2 oz American cheese (275)

D:

- Replace half pepperoni pizza (310) with half veggie pizza (285)
- Replace ¾ pepperoni pizza (465) with ¾ veggie pizza (430)
- Replace pepperoni pizza (620) with veggie pizza (560)

Sat:

B:

- Replace 2 oz turkey bacon (70) with 2 oz scrambled eggs (90)
- Replace 3 oz turkey bacon (105) with 2 oz scrambled eggs (90)
- Replace 4 oz turkey bacon (140) with 2 oz scrambled eggs with 1 oz American cheese (165)

D:

- Replace chicken in LoMein with tofu

Sun:

B:

- Replace sausage patty (180) with vanilla yogurt (150)
- Replace 2 sausage patties (360) with vanilla yogurt (150), 2 oz raisins (130), 1 oz granola (60)
- Replace 3 sausage patties (540) with vanilla yogurt (150), 2 oz raisins (130), Tastykake© Koffee Kake Junior (280)

L:

- Replace turkey sandwich (360) with hummus and veggie wrap with additional 1 oz of hummus added to wrap (375)
- Replace turkey sandwich (470) with hummus and veggie wrap with additional 2 oz of hummus added to wrap (450)

Malnutrition Pathway Menu Modifications

- Replace turkey sandwich (580) with hummus and veggie wrap with additional 2 oz of hummus added to wrap (450), replace broccoli and butter (55) with 1 cup cooked peas (100) and 1 pat butter (35)

D:

- Replace 2 chicken tenders (200) with 4 fish sticks (220) offered with 2 packets ketchup
- Replace 3 chicken tenders (300) with 6 fish sticks (330) offered with 2 packets ketchup
- Replace 4 chicken tenders (400) with 6 fish sticks (330) offered with 3 packets ketchup and extra ¼ cup french fries (90)
- Replace 5 chicken tenders (500) with 6 fish sticks (330) offered with 3 packets ketchup and extra ½ cup french fries (180)
- Replace 6 chicken tenders (600) with 6 fish sticks (330) offered with 3 packets ketchup and extra ¾ cup fries (270)

Malnutrition Pathway Menu Modifications

12. <u>Vegetarian - Lacto-Ovo Vegetarian Modifications to MP</u>

Mon :

L:

- Replace tilapia (130) with ½ peanut butter and jelly sandwich on wheat (160)

D

- Replace grilled chicken breast (140) with 4 oz grilled tofu (145)
- Replace 1.5 grilled chicken breasts (210) with 6 oz grilled tofu (210)
- Replace 2 grilled chicken breasts (280) with 8 oz grilled tofu (280)
- Replace 2.5 grilled chicken breasts (350) with 8 oz grilled tofu (180) and 2 packets BBW sauce for tofu (70)

Tues :

L:

- Replace 1 meatball (100) with 1 oz mozzarella cheese melted onto pasta (70)
- Replace 2 meatballs (200) with 1 oz mozzarella cheese melted onto pasta (70) and breadstick (110)
- Replace 3 meatballs (300) with 1 oz mozzarella cheese melted onto pasta (70) and breadstick (110), replace Rice Krispie Treat with Hershey Incredible Cone (300)
- Replace 5 meatballs (500) with 3 oz mozzarella cheese melted onto pasta (210) and replace Rice Krispie Treat with Hershey Incredible Cone (300)

D:

- Replace roast turkey (185), ½ cup mashed potatoes (130), broccoli (20) with 3 broccoli and cheddar potato skins (420)
- Replace roast turkey and gravy (225), ½ cup mashed potatoes (130), broccoli with butter (35) with 3 broccoli and cheddar potato skins (420)
- Replace roast turkey and gravy (225), ¾ cup mashed potatoes (195), broccoli with butter (35) with 3 broccoli and cheddar potato skins (420)
- Replace roast turkey and gravy (225), 1 cup mashed potatoes (260), broccoli with butter (35) with 4 broccoli and cheddar potato skins (560)
- Replace roast turkey and gravy (290), 1 cup mashed potatoes (260), broccoli with butter (35) with 4 broccoli and cheddar potato skins (560)
- Replace roast turkey and gravy (450), 1 cup mashed potatoes (260), broccoli with butter (35) with 5 broccoli and cheddar potato skins (700)

Wed:

L:

- Replace half hamburger (190) with half black bean veggie burger (140)
- Replace hamburger (375) with black bean veggie burger (280) with 1 oz of American cheese (75)
- Replace cheeseburger (450) with black bean veggie burger with 2 oz of American cheese (430)
- Replace cheeseburger (525) with black bean veggie burger with 2 oz of American cheese (430) and 2 oz avocado on burger (90)
- Replace cheeseburger (600) with black bean veggie burger with 3 oz American cheese (500) and 2 oz avocado on burger (90)

Malnutrition Pathway Menu Modifications

D:
- Replace chicken with tofu in pasta alfredo

Thurs:
B:
- Replace 1 link turkey sausage (70) with 2 oz scrambled eggs (90)
- Replace 2 links turkey sausage (140) with 2 oz scrambled eggs with 1 oz American cheese (165)
- Replace sausage patty (180) with 2 oz scrambled eggs with 1 oz American cheese (165)
- Replace 2 sausage patties (360) with 4 oz scrambled eggs with 2 oz American cheese (330)
- Replace 3 sausage patties (540) with 5 oz scrambled eggs with 3 oz American cheese (435) and 3 pats butter (105)

D:
- Replace beef (120) with 4 oz tofu (145) in fajita

Fri:
B:
- 4000-4600 kcal MP: replace 1 link turkey sausage (70) with 2 oz scrambled eggs (90)
- 4800-5000 kcal MP: replace 2 links turkey sausage (140) with 2 oz scrambled eggs with 1 oz American cheese (165)
- 5200-6000 kcal MP: replace 4 links turkey sausage (280) with 3 oz scrambled eggs with 2 oz American cheese (275)

D:
- Replace half pepperoni pizza (310) with half veggie pizza (285)
- Replace ¾ pepperoni pizza (465) with ¾ veggie pizza (430)
- Replace pepperoni pizza (620) with veggie pizza (560)

Sat:
B:
- Replace 2 oz turkey bacon (70) with 2 oz scrambled eggs (90)
- Replace 3 oz turkey bacon (105) with 2 oz scrambled eggs (90)
- Replace 4 oz turkey bacon (140) with 2 oz scrambled eggs with 1 oz American cheese (165)

L:
- Replace tuna sandwich (340) with cheese hoagie made on 6" hoagie roll with 1 oz American cheese, lettuce, tomato and mustard (375)
- Replace tuna sandwich (450) with cheese hoagie made on 6" hoagie roll with 2 oz American cheese, lettuce, tomato and mustard (450)
- Replace tuna sandwich (560) with cheese hoagie on 6” hoagie roll with 4 oz American cheese, lettuce, tomato, mustard (590)

D
- Replace chicken in LoMein with tofu

Malnutrition Pathway Menu Modifications

Sun:
B:
- Replace sausage patty (180) with vanilla yogurt (150)
- Replace 2 sausage patties (360) with vanilla yogurt (150), 2 oz raisins (130), 1 oz granola (60)
- Replace 3 sausage patties (540) with vanilla yogurt (150), 2 oz raisins (130), Tastykake© Koffee Kake Junior (280)

L:
- Replace turkey sandwich (360) with hummus and veggie wrap with additional 1 oz of hummus added to wrap (375)
- Replace turkey sandwich (470) with hummus and veggie wrap with additional 2 oz of hummus added to wrap (450)
- Replace turkey sandwich (580) with hummus and veggie wrap with additional 2 oz of hummus added to wrap (450), replace broccoli and butter (55) with 1 cup cooked peas (100) and 1 pat butter (35)

D:
- Replace 2 chicken tenders and fries (390) with burrito made with flour tortilla, 1 oz refried beans, 2 oz avocado, 1/4 cup rice, 1 oz shredded cheddar, salsa (430)
- Replace 3 chicken tenders and fries (490) with burrito made with flour tortilla, 1 oz refried beans, 2 oz avocado, 1/4 cup rice, 1 oz shredded cheddar, salsa, 1 oz sour cream (480)
- Replace 3 chicken tenders and fries (585) with burrito made with flour tortilla, 2 oz refried beans, 2 oz avocado, 1/2 cup rice, 1 oz shredded cheddar, salsa, 1 oz sour cream (580)
- Replace 4 chicken tenders and fries (685) with burrito made with flour tortilla, 2 oz refried beans, 4 oz avocado, 1/2 cup rice, 1 oz shredded cheddar, salsa, 1 oz sour cream (670)
- Replace 5 chicken tenders and fries (860) with burrito made with flour tortilla, 2 oz refried beans, 4 oz avocado, 1/2 cup rice, 1 oz shredded cheddar, salsa, 1 oz sour cream (670) and 2 oz baked tortilla chips (180) with salsa cup (30)
- Replace 6 chicken tenders and fries (960) with burrito made with flour tortilla, 2 oz refried beans, 4 oz avocado, 1/2 cup rice, 1 oz shredded cheddar, salsa, 2 oz sour cream (670) and 2 oz baked tortilla chips (180) with salsa cup (30) and 1 oz cheese sauce for chips (70)
