# Supplementary material for: Outcomes of an inpatient medical nutritional rehabilitation protocol in children and adolescents with eating disorders
Source: J Eat Disord. 2017 Mar 1;5:7. doi: 10.1186/s40337-017-0134-6 (PMC5331684; doi:10.1186/s40337-017-0134-6)
Supplement: Additional file 2: Appendix B. — Nutrition education on inpatient meal plan and home hospitalization. (PDF 197 kb) [file 40337_2017_134_MOESM2_ESM.pdf]

40:B:66

Information for Caregivers
Malnutrition, Weight Loss, and Eating Disorders
Menu Selection on the CHOP Nutrition Support Plan

Many children and adolescents struggle with food and eating. Your child/adolescent has been admitted to the hospital because we are concerned about his/her nutrition. A balanced and complete diet will be important during recovery. We believe that process starts with menu plans that include a variety of different foods. The menus are designed to help rebuild muscle mass and other lost body stores. Some of the items listed may be foods that you serve at home and some may not. We may also re-introduce foods that you have had to stop serving over the past few months or years. It will be important to challenge your child/adolescent with new and different foods during this hospitalization. This will help to improve his/her ability to accept a similar variety of foods after returning home. We understand this may be difficult at first, but it's important to start developing healthy nutrition habits now.

One part of the nutrition support plan includes a fixed menu at meal and snack times. This can be frustrating for your child/adolescent at first, but over time it will help to reduce anxiety by encouraging a variety of foods without the stress of having to choose which ones to eat. Some important features are:

- Your child/adolescent will be served a 7-day rotating menu, with different meals each day of the week. These foods have been carefully chosen for the best variety of nutrients.
- If your child/adolescent has a true food allergy, or if your family has practiced a certain diet for religious or other beliefs since before any disordered eating began, please discuss that with your physicians so that we can discuss how best to consider possible substitutions.
- If your child/adolescent has true lactose intolerance, we may give him/her lactase enzymes before any foods and beverages that contain lactose (milk, cheese, yogurt, etc.). We will strongly encourage him/her to eat all of the food on the meal tray. Any items he does not finish will be replaced by Boost, a lactose free supplement.
- Condiments such as ketchup, mustard, etc (in reasonable quantities) are allowed, but must be asked for in advance.

This system of meal selection ensures that your child/adolescent receives a balanced, varied diet while in the hospital. There are many members on the health care team. The best people to ask about nutrition would be the nutrition team or the physicians during the day.

Welcome to CHOP! We truly hope to help your child/adolescent return to health.

Written 1/11
Revised 3/4/13

©The Children's Hospital *of* Philadelphia 2011. Not to be copied or distributed without permission. All rights reserved.
Patient family education materials provide educational information to help individuals and families. You should not rely on this information as professional medical advice or to replace any relationship with your physician or healthcare provider.

Caring for Your Child

Home Hospital Nutrition Education

You, as the caregiver, are now in charge of everything to do with your child's food. You will grocery shop, meal plan, prepare and plate the food and supervise all eating occasions. Your child's only job is to eat and rest!

Key Points:

- Your child should eat 3 meals and 2-3 snacks each day.
- Meals should be 30 minutes long with a 60 minute rest period. Snack should be 15 minutes long with a 15 minute rest period.
- For uneaten food, supplement with Boost as you were taught. Give your child 15 minutes to drink the Boost.
- During rest periods, your child should not use the bathroom.
- Stay with your child any time he is eating or resting.
- Meals should include foods from all the different food groups (meat, starch, dairy, fruits, vegetables, fats).
- Avoid diet or "health foods," caffeine and chewing gum.
- Include a calorie-containing beverage with all meals and snacks. Water is great in between meals and snacks.
- Any foods that your child used to eat but has recently been avoiding should be added back.
- Don't be surprised if your child does not feel hungry yet.
- Your child is malnourished and requires a lot of calories to recover. The rest of the family may not need as much food as your child does right now.
- Your child's only interaction with food should be eating it. He should not cook, bake, grocery shop, watch food-related shows, read recipes, etc.
- No activity until discussing with outpatient team.

Caregiver's Job:

- Plan all eating occasions at least one day ahead of time.
- Keep a food log and add up the total calories (Calorie King © and MyFitness Pal ©)
- Bring the completed food log to your outpatient RD visit.

Written 11/15

©The Children's Hospital *of* Philadelphia 2015. Not to be copied or distributed without permission. All rights reserved.
Patient family education materials provide educational information to help individuals and families. You should not rely on this information as professional medical advice or to replace any relationship with your physician or healthcare provider.
